# A Temporal -omic Study of *Propionibacterium freudenreichii* CIRM-BIA1^T^ Adaptation Strategies in Conditions Mimicking Cheese Ripening in the Cold

**DOI:** 10.1371/journal.pone.0029083

**Published:** 2012-01-13

**Authors:** Marion Dalmasso, Julie Aubert, Valérie Briard-Bion, Victoria Chuat, Stéphanie-Marie Deutsch, Sergine Even, Hélène Falentin, Gwénaël Jan, Julien Jardin, Marie-Bernadette Maillard, Sandrine Parayre, Michel Piot, Jarna Tanskanen, Anne Thierry

**Affiliations:** 1 French National Institute for Agricultural Research, UMR1253 Science et Technologie du Lait et de l'Œuf, Rennes, France; 2 AGROCAMPUS OUEST, UMR1253 Science et Technologie du Lait et de l'Œuf, Rennes, France; 3 French National Institute for Agricultural Research, CIRM-BIA, UMR1253 Science et Technologie du Lait et de l'Œuf, Rennes, France; 4 French National Institute for Agricultural Research, UMR518 Mathématiques et Informatique Appliquées, Paris, France; 5 AgroParisTech, UMR518 Mathématiques et Informatique Appliquées, Paris, France; 6 Valio Ltd, Helsinki, Finland; Argonne National Laboratory, United States of America

## Abstract

*Propionibacterium freudenreichii* is used as a ripening culture in Swiss cheese manufacture. It grows when cheeses are ripened in a warm room (about 24°C). Cheeses with an acceptable eye formation level are transferred to a cold room (about 4°C), inducing a marked slowdown of propionic fermentation, but *P. freudenreichii* remains active in the cold. To investigate the *P. freudenreichii* strategies of adaptation and survival in the cold, we performed the first global gene expression profile for this species. The time-course transcriptomic response of *P. freudenreichii* CIRM-BIA1^T^ strain was analyzed at five times of incubation, during growth at 30°C then for 9 days at 4°C, under conditions preventing nutrient starvation. Gene expression was also confirmed by RT-qPCR for 28 genes. In addition, proteomic experiments were carried out and the main metabolites were quantified. Microarray analysis revealed that 565 genes (25% of the protein-coding sequences of *P. freudenreichii* genome) were differentially expressed during transition from 30°C to 4°C (*P*<0.05 and |fold change|>1). At 4°C, a general slowing down was observed for genes implicated in the cell machinery. On the contrary, *P. freudenreichii* CIRM-BIA1^T^ strain over-expressed genes involved in lactate, alanine and serine conversion to pyruvate, in gluconeogenesis, and in glycogen synthesis. Interestingly, the expression of different genes involved in the formation of important cheese flavor compounds, remained unchanged at 4°C. This could explain the contribution of *P. freudenreichii* to cheese ripening even in the cold. In conclusion, *P. freudenreichii* remains metabolically active at 4°C and induces pathways to maintain its long-term survival.

## Introduction

Micro-organisms have a determining contribution to the formation of the typical flavor and texture characteristics of each cheese variety. The metabolism of lactic starters and of adjunct ripening cultures has mainly been studied in conditions mimicking their growth in milk and in cheese. More recently, studies have specifically focused on the metabolism of non-growing bacteria under the sub-optimal conditions prevailing in cheese. They showed that bacterial metabolism is markedly modified by cheese conditions, like starvation and low temperatures. Bacteria expressed genes associated with specific metabolic pathways that could also contribute to the ripening process [1]–[4].

*Propionibacterium freudenreichii* is widely used in Swiss cheese manufacture for its key contribution to the formation of the characteristic holes (or “eyes”) and flavor during the ripening of this cheese variety [5]. Different combinations of time and temperature are applied during the ripening of Swiss cheeses, according to the manufacturers' practices. Typically, cheeses are ripened for 2–3 weeks at 10–14°C, then in a warm room (20–24°C) until the cheeses have enough holes, and are finally transferred to a cold room (4–6°C). *P. freudenreichii* grows during the ripening in the warm room with populations reaching stable levels over 10^9^ colony-forming units (cfu)/g [1]. It converts the lactate produced by lactic acid bacteria into propionate, acetate and CO_2_. Propionic fermentation mainly occurs in the warm room and is markedly slowed down in the cold room. However, *P. freudenreichii* is known to remain active during the storage of cheese at 4°C, as recently shown by real time reverse transcription PCR [1]. In particular, it goes on producing some flavor compounds in the cold, like short branched-chain acids from branched-chain amino acids catabolism, and free fatty acids from milk fat hydrolysis [6]. The aim of the present study was to better understand how *P. freudenreichii* copes with cold-induced stress and remains active when cheeses are transferred to the cold room. This study was performed under conditions mimicking Swiss cheese ripening, by applying for the first time a whole genome transcriptomic approach to *P. freudenreichii*. Transcriptomics and other -omic approaches have increasingly been used over the last few years to provide a comprehensive view of microorganism physiology in various research areas. For example, the transcriptomic response of the starter bacterium *Lactococcus lactis* has recently been studied in conditions mimicking cheese manufacture [7].

The time-course transcriptomic response of *P. freudenreichii* CIRM-BIA1^T^ strain was analyzed during its growth at 30°C, then during a further 9 days at 4°C. In addition, temporal changes in the proteome and extracellular metabolome were monitored. Our results show that *P. freudenreichii* under-expresses many genes involved in cell machinery in the cold, but remains active, redirecting its carbon metabolism towards glycogen accumulation. Interestingly, some genes involved in the formation of important flavor compounds of cheese were not significantly affected in the cold. These results highlight the contribution of *P. freudenreichii* to the formation of flavor compounds even at low ripening temperatures. Thus, our data provides insights into the strategies used by *P. freudenreichii* to adapt to a low temperature and to maintain its long-term survival.

## Results and Discussion

### Experimental conditions mimicking cheese ripening

In cheeses stored at cold temperatures, *P. freudenreichii* stops growing, slows down propionic fermentation, but it remains active [1] and continues to produce some metabolites [6]. To better understand how *P. freudenreichii* CIRM-BIA1^T^ copes with cold-induced stress when cheeses are transferred to the cold room, an -omic approach was applied. The culture conditions were chosen to mimic the medium and the temperature-shift withstood by *P. freudenreichii* during Swiss cheese ripening.

**Figure 1:**
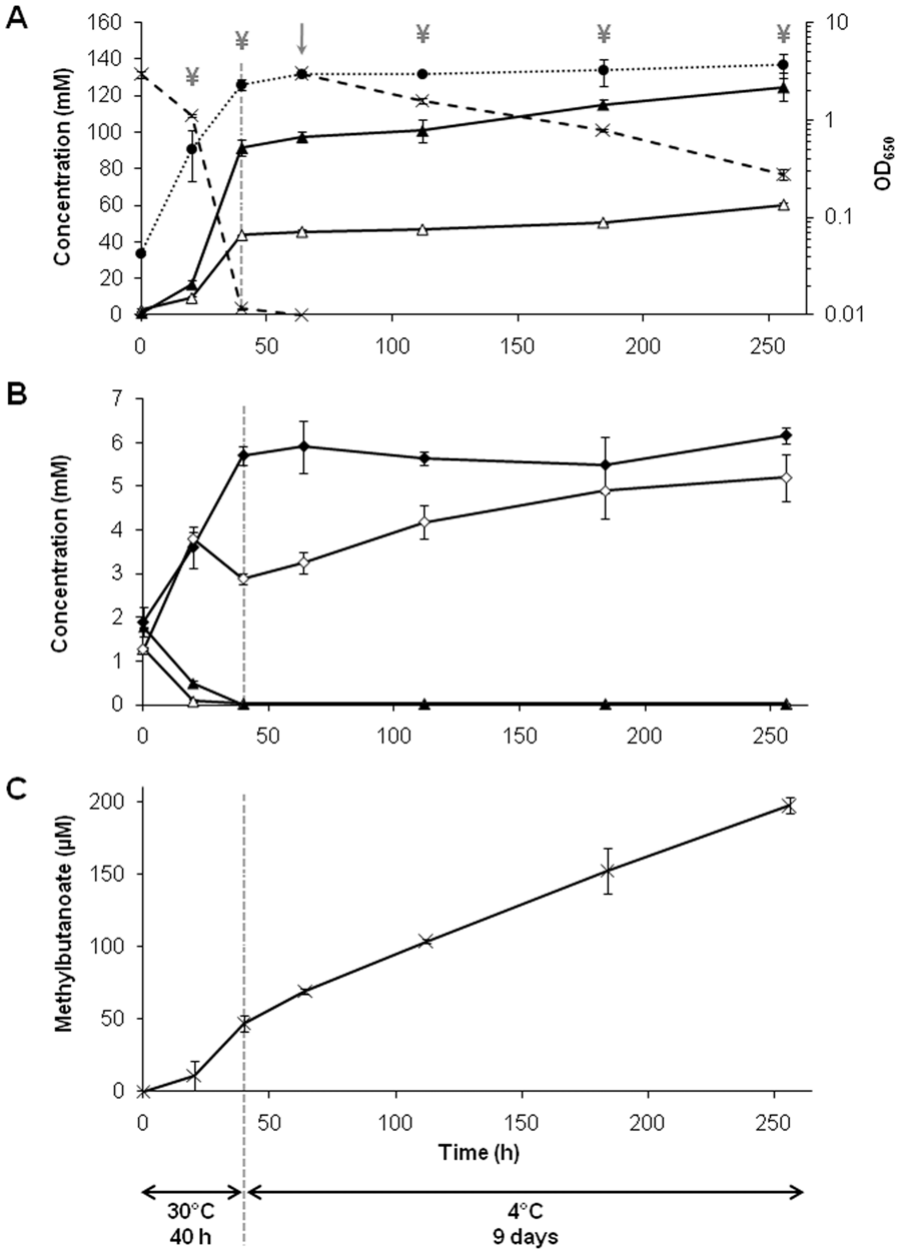
Time-course of *P. freudenreichii* CIRM-BIA1^T^ metabolic activity over a 40 h-incubation at 30°C followed by a further 9 days at 4°C. Lactate was added at 64 h (**↓**) to mimic cheese ripening conditions. **A**, growth monitored by optical density (650 nm) measurements (black circle), lactate consumption (cross), production of acetate (white triangle) and propionate (black triangle); **B**, production of pyruvate (white rhombus) and succinate (black rhombus), consumption of aspartate (white triangle) and asparagine (black triangle); **C**, production of methylbutanoate (sum of 2-methyl- and 3-methylbutanoate acids). ¥: sampling times for microarray experiments.

During Swiss cheese ripening, *P. freudenreichii* grows in the absence of carbohydrates since all the lactose of milk is converted to lactate by lactic starters within the first day of cheesemaking [5]. Lactate is used as the main carbon source by *P. freudenreichii* and is available until the end of ripening [5], [8], [9]. The Swiss cheese aqueous phase is a rich medium, in which the soluble nitrogen matter increases during ripening from about 20 g/kg to 50–150 g/kg, with a proportion of free amino acids reaching up to 50% [9]. To mimic cheese conditions, bacteria were grown in a rich medium (YEL [10]) containing lactate (130 mM) as carbon source and high amounts of soluble nitrogen matter (Table S1), and as follows: at 30°C for 40 h, lactate was then added to prevent its exhaustion, and the cultures were further incubated for 9 days at 4°C (Fig. 1A). Preliminary experiments showed that, in YEL medium, either containing twice as much initial lactate content (260 mM instead of 130 mM), or supplemented with 130 mM lactate during incubation, *P. freudenreichii* CIRM-BIA1^T^ continued, or started growing again at the same rate if the temperature was maintained at 30°C, and consumed all the available lactate (Fig.S1). These results showed that low temperature, and not nutrient starvation, was the main factor inducing changes in CIRM-BIA1^T^ metabolism under our experimental conditions, like in Swiss cheese.

The time-course of growth, lactate consumption and propionic fermentation are shown in Figure 1A. Cells grew at 30°C with a generation time of 5.4±0.2 h. They consumed lactate and concomitantly produced propionate and acetate, with a ratio of 2.2±0.1 moles of propionate per mole of acetate, in agreement with literature data [5], [11], [12]. Pyruvate was excreted during exponential growth when lactate was abundant, and then reused when the lactate concentration was low, at the end of incubation at 30°C (40 h) (Fig. 1B). As previously observed under similar conditions, *P. freudenreichii* can excrete pyruvate in order to maintain its concentration in the cell under its toxicity threshold [13].

Succinate and NH_3_ were also produced, concomitantly with aspartate (Asp) and asparagine (Asn) consumption (Fig. 1B, Table S1). The molar ratio of succinate, NH_3_, and Asp/Asn was close to the expected ratio of 1. *P. freudenreichii* is known to deaminate Asp to fumarate which is, in turn, converted to succinate, with a generation of additional energy. Previous studies performed under similar conditions showed that, when extra Asp was added to a complex medium containing lactate, up to 40 mM of Asp were converted to succinate concomitantly with lactate fermentation [11]. In Swiss cheese, Asp and Asn are also metabolized by *P. freudenreichii* and, consequently, their final content is much lower than in similar cheese varieties without propionic fermentation [8]. In the present study, the concentration of six other free amino acids, glutamate (Glu), glycine (Gly), serine (Ser), arginine (Arg), lysine (Lys) and alanine (Ala) also exhibited a decrease ranging from 0.22 to 1.06 mM during the incubation at 30°C (Table S1). Short chain acids (acetate, propionate and methylbutanoic acids) are considered as flavor compounds in Swiss cheese [6]. Beside acetate and propionate, CIRM-BIA1^T^ also produced 46.8±5.2 µM of methylbutanoic acids during its growth at 30°C (Fig. 1C).

All these results showed that the metabolites produced by *P. freudenreichii* CIRM-BIA1^T^ during growth in cultures at 30°C were in close agreement with that produced during its growth in Swiss cheese.

### Time-course of gene expression over the incubation period

A whole-genome transcriptomic approach was used to investigate the gene expression of *P. freudenreichii* CIRM-BIA1^T^ at five sampling times. Overall, 565 genes were detected as differentially expressed (DE genes) with *P*<0.05 and |(fold change)|>1, at least at one sampling time in comparison with the reference time 20 h. The DE genes represented 25% of the protein-coding genes of CIRM-BIA1^T^ genome targeted in the microarray. The complete microarray expression data for these genes is presented in Table S2.

To get information on the time-course of gene expression, the temporal expression profiles of the DE genes were classified using a quadratic regression method, convenient to analyze time-course microarray data [14]. A large majority (80%) of genes exhibited two patterns characterized by i) a marked increase or decrease in expression occurring between the incubation periods at 30°C and at 4°C, and ii) a limited change in expression over the period at 4°C (Fig.S2). In addition, 19% of genes exhibited a linear decrease or increase, and 1% showed a transient up- or down-regulation at 40 h that could not be fitted by this quadratic regression model at *P*<0.05.

**Figure 2:**
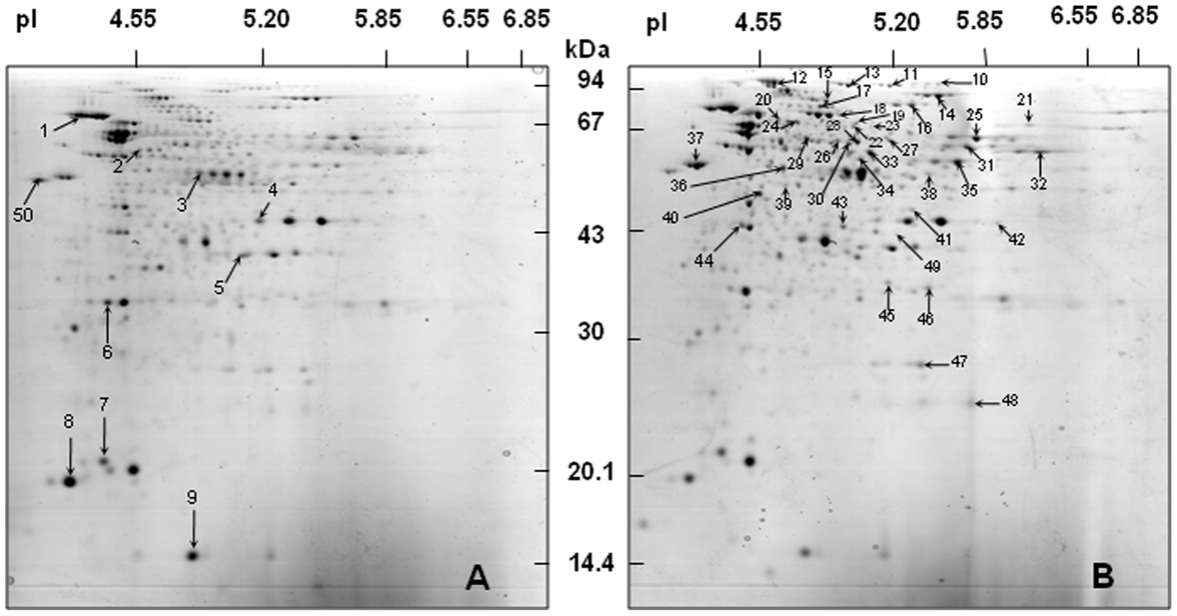
Two-dimensional analysis of proteins produced during *P. freudenreichii* CIRM-BIA1^T^ growth (**A**) at 30°C (reference time 20 h) and then (**B**) at 4°C (3 days). Numbers identify spots which volume decreased at 4°C (**A**), or increased at 4°C (**B**). The identification by MS/MS of each spot can be found in Table S4.

According to these temporal expression patterns, the results will be further discussed by comparing gene expression at two main sampling times, i.e. at the reference time 20 h, corresponding to the exponential phase of growth at 30°C, and after 3 days at 4°C. In agreement, RT-qPCR validation experiments were solely performed at these two sampling times. RT-qPCR was applied to 28 genes observed as differentially expressed in microarray experiments, preferentially chosen (19/28) among the up-regulated genes at 4°C. Overall, the confirmation of microarray data by RT-qPCR showed an excellent agreement between the fold-change values obtained from the two approaches (RT-qPCR fold change = 1.17×microarray fold change, R^2^ = 0.91), thus validating the results of microarray experiments (Tables 1, 2, 3 and 4). Moreover, the changes in protein expression between 30°C and 4°C were analyzed by using a proteomic approach combining 2D electrophoresis and protein identification by mass spectrometry (Fig. 2, Table S4).

**Table 1:** Time-course comparison of transcriptome and proteome changes in *P. freudenreichii* CIRM-BIA1^T^ strain, for genes involved in metabolic categories CD, CE, DNA, E, L, Mi, Nt, and P.

Gene				Fold change (log2)											
				Microarray					RT-qPCR		2D				
Name	Locus tag	Description	Category	40 h	3 days	6 days	9 days	*P*-value	3 days	*P*-value	3 days	6 days	9 days	*P*-value	Spot No.^a^
*ftsX*	PFREUD_09600	Cell division protein	CD	−0.9	−4.2	−3.7	−2.8	**1.80E-05**	−4.3	**0.046**					
*dpm*	PFREUD_10950	Dolichyl-phosphate β-D-mannosyltransferase	CE	0.1	−2.1	−2.2	−1.9	**1.10E-04**			0.8	1.1	0.9	0.001	45
*gyrB1*	PFREUD_12820	DNA gyrase subunit B	DNA	−0.4	−1.1	−0.3	−0.3	0.432	−1.7	**0.05**					
*nuoC*	PFREUD_05180	NADH-quinone oxidoreductase chain C	E	0	−3.6	−3.2	−3.2	**3.10E-04**			1	1.2	0.8	8.40E-05	46
*nuoD*	PFREUD_05190	NADH-quinone oxidoreductase chain D	E	0.3	−4	−3.3	−3.8	**0.002**			−0.8	−1	−0.9	0.003	2
*atpA*	PFREUD_10470	ATP synthase subunit α	E	−0.8	−3.6	−3.4	−3.9	**2.90E-04**			0.4	0.8	0.6	0.013	22
*sdhC2*	PFREUD_14320	Succinate dehydrogenase cytochrome B-558 subunit	E	0.8	−2.1	−2.1	−1.4	**0.002**	−3.3	**0.025**					
*acs*	PFREUD_23780	Fatty-acyl-CoA synthase	L	−0.3	−0.3	−0.3	−0.2	0.526			0.4	1.1	0.7	0.046	19
*sodA*	PFREUD_06110	Fe/Mn superoxide dismutase	Mi	0.6	1.4	0.3	−0.5	0.064			0.6	0.8	0.6	0.003	47
NC	PFREUD_01850	Dihydroorotate dehydrogenase	Nt	0.1	0.5	0.7	0.5	0.595			1.6	0.9	0.3	0.02	40
*guaB1*	PFREUD_06480	Inosine-5 -monophosphate dehydrogenase	Nt	0.1	−1.1	−1.3	−1.3	**0.002**			1.2	0.5	0.2	0.043	21
*guaB2*	PFREUD_06490	Inosine-5 -monophosphate dehydrogenase	Nt	0.1	0.1	0.1	0	0.899			0.8	0.7	0.6	0.044	42
*guaA*	PFREUD_06680	GMP synthase	Nt	−0.4	−1	−1.1	−1.5	**0.011**			0.5	0.9	0.8	0.006	24
*glmU*	PFREUD_17410	UDP-N-acetylglucosamine pyrophosphorylase	Nt	0	−0.3	−0.1	−0.4	0.79			0.6	1	0.4	0.013	23
*rplL*	PFREUD_05580	50S ribosomal protein L7/L12	P	−0.6	−0.5	−1	−1.8	0.225			−0.7	−1	−1.1	0.001	8
*tuf*	PFREUD_05650	Elongation factor Tu	P	−0.1	−2.3	−2.4	−2.3	**0.001**	−1.9	**0.066**	−1.7	−1.4	−1.3	4.70E-05	3
*metG*	PFREUD_06960	Methionyl-tRNA synthetase	P	−0.1	−1.4	−1.2	−1.2	**0.011**			0.9	0.3	0.9	0.034	20
*alaS*	PFREUD_11560	Alanyl-tRNA synthetase	P	−0.6	−1.3	−1	−1.1	**0.013**			0.4	0.4	−0.9	0.035	10

a Spot number (see Table S4 and Fig. 2).

Bold characters indicate genes that are differentially expressed according to microarray experiments (*P*<0.05 and |fold change|>1 for at least one sampling time) and RT-qPCR (*P*<0.05) experiments.

**Table 2:** Time-course comparison of transcriptome and proteome changes in *P. freudenreichii* CIRM-BIA1^T^ strain, for genes involved in metabolic categories A, AA and C.

Gene				Fold change (log2)											
				Microarray					RT-qPCR		2D				
Name	Locus tag	Description	Category	40 h	3 days	6 days	9 days	*P*-value	3 days	*P*-value	3 days	6 days	9 days	*P*-value	Spot No.^a^
*dps*	PFREUD_02870	Starvation-inducible DNA-binding protein	A	0.3	3.3	2.3	1.2	**0.005**	2.7	**0.002**					
*pspC*	PFREUD_06710	Stress-response transcriptional regulator	A	0.5	3.2	2.6	2.2	**0.006**	3.8	**0.006**					
*cstA*	PFREUD_16500	Carbon starvation protein	A	2.3	−1.4	−1.4	−0.6	**2.60E-04**	−1.3	**0.045**					
*cspB*	PFREUD_18210	Cold shock protein	A	0.1	2.6	1.9	1	**0.038**	0.1	0.854					
*katA*	PFREUD_23800	Catalase	A	1	1.6	0.8	0.1	0.163			1.5	1.7	1.4	1.10E-04	28
*ald*	PFREUD_00370	Alanine dehydrogenase	AA	4.3	7.3	6.8	6.6	**3.00E-06**	9.5	**0.042**	0.8	0.8	1.4	5.70E-05	36
*argG*	PFREUD_01460	Argininosuccinate synthase	AA	−0.4	2.9	2.3	0.6	**0.003**	4.1	**0.019**					
*bkdA2*	PFREUD_02200	Branched-chain α-keto acid dehydrogenase	AA	−0.9	−3.2	−3	−2.5	**0.011**	−4.2	**0.024**					
*tyrB*	PFREUD_09460	Aspartate transaminase	AA	−0.9	−2.4	−2.3	−2.3	**3.00E-06**	−4.8	**0.022**					
*livG*	PFREUD_10850	ABC transporter of branched-chain amino acid	AA	−0.4	−3.3	−2.9	−3.1	**0.002**	−2	0.084					
*ilvE*	PFREUD_13350	Branched-chain amino acid aminotransferase	AA	0.1	0.3	0.2	−1	0.254			0.1	0.3	0.8	0.001	43
*argJ*	PFREUD_13980	Arginine biosynthesis bifunctional protein	AA	−0.2	3.4	2.8	1.2	**0.001**	3.6	**0.003**					
*aspA2*	PFREUD_16330	Aspartate ammonia-lyase	AA	1.5	2.8	2.5	2	**0.001**	3.2	**4.90E-04**	0.9	0.9	0.9	0.002	33
*cys2*	PFREUD_16420	Cysteine synthase 2	AA	0	0.1	0.1	0.6	0.516			−0.8	−0.9	−0.9	2.40E-04	5
*sdaA*	PFREUD_18570	L-serine dehydratase	AA	0.2	2	1.6	0.9	**0.003**	3.5	**0.005**					
*asd*	PFREUD_20100	Aspartate-semialdehyde dehydrogenase	AA	0.1	1	0.5	0.2	0.235			0.4	0.6	0.4	0.048	41
*bluB*	PFREUD_06370	Phosphoribosyltransferase	C	0	−0.8	−1.1	−1.6	**0.029**			0.2	0.7	0.9	0.004	17

a Spot number (see Table S4 and Fig. 2).

Bold characters indicate genes that are differentially expressed according to microarray experiments (*P*<0.05 and |fold change|>1 for at least one sampling time) and RT-qPCR (*P*<0.05) experiments.

**Table 3:** Time-course comparison of transcriptome and proteome changes in *P. freudenreichii* CIRM-BIA1^T^ strain, for genes involved in metabolic category Ph and PM.

Gene				Fold change (log2)											
				Microarray					RT-qPCR		2D				
Name	Locus tag	Description	Category	40 h	3 days	6 days	9 days	*P*-value	3 days	*P*-value	3 days	6 days	9 days	*P*-value	Spot No.^a^
*NC*	PFREUD_18300	NUDIX hydrolase	Ph	0.3	1.7	0.6	−0.3	0.077	2.9	**0.006**					
*ppa*	PFREUD_23500	Inorganic pyrophosphatase	Ph	0.6	3.1	1.7	1.3	**0.003**	3.1	**2.40E-04**					
*dnaK2*	PFREUD_04630	Chaperone protein	PM	−0.1	−2.4	−2.5	−3	**0.004**			−0.8	−0.6	−0.5	1.70E-04	1
*groS1*	PFREUD_06460	10 kDa chaperonin 1	PM	−1.2	−5	−4.9	−5.2	**1.10E-04**			−0.8	−1.1	−0.9	5.80E-05	9
*groL1*	PFREUD_06470	60 kDa chaperonin 1	PM	−0.8	−3.4	−3.7	−3.9	**0.001**	−4.4	**0.036**					
*clpB 2*	PFREUD_17920	Chaperone	PM	−0.5	−2.9	−2.8	−2.7	**4.80E-04**			−0.3	0.4	0.3	0.012	14
*clpB 1*	PFREUD_19250	Chaperone clpB 1	PM	−0.1	0.8	0.7	0	0.185			0.5	1.1	1.3	0.009	13
*clpC*	PFREUD_20250	chaperone clpC	PM	−0.2	−0.5	−0.4	−1	0.598			0.5	1	0.2	0.035	11
*hsp20 1*	PFREUD_22780	Heat shock protein	PM	0.8	2.8	2.7	2.5	**0.001**			−0.6	−0.9	−0.5	0.049	7
NC	PFREUD_03120	Hypothetical protein		−0.2	−2.3	−2.3	−2.6	**4.30E-04**			0.7	0.6	0.8	0.027	39
NC	PFREUD_04430	Thiamine pyrophosphate enzyme		0.1	−0.8	−0.9	−0.6	**0.026**			0.8	0.6	0.7	0.007	26
NC	PFREUD_15430	Hypothetical protein		0.5	−1.3	−0.9	−1.1	0.088			−0.8	−0.7	−0.3	0.025	44
NC	PFREUD_18630	Hypothetical protein		0	0.1	0	−0.8	0.698			−1	−1.3	−0.9	0.003	6
NC	PFREUD_11600	Hypothetical protein		1.3	1.2	1.0	0.3	0.14			1.3	1.5	0.1	3.32E-04	49
*rpo*A	PRREUD_06070	DNA-directed RNA polymerase α-chain		−1.2	−4.0	−3.4	−3.7	**0.022**			0.4	0.4	0.0	0.01	50

a Spot number (see Table S4 and Fig. 2).

Bold characters indicate genes that are differentially expressed according to microarray experiments (*P*<0.05).

**Table 4:** Time-course comparison of transcriptome and proteome changes in *P. freudenreichii* CIRM-BIA1^T^ strain, for genes involved in metabolic category CH.

Gene				Fold change (log2)											
				Microarray					RT-qPCR		2D				
Name	Locus tag	Description	Category	40 h	3 days	6 days	9 days	*P*-value	3 days	*P*-value	3 days	6 days	9 days	*P*-value	Spot No.^a^
*cat*	PFREUD_03110	yme A transferase	CH	0.5	−0.1	−0.4	−1	0.124			1.2	0.9	0.8	3.80E-05	32
*ppdk*	PFREUD_03230	yruvate phosphate dikinase	CH	1.6	1.9	2.2	1.4	**0.007**	2.5	**0.004**	1.4	1.2	1.9	0.021	12
*pgi*	PFREUD_04290	ose-6-P isomerase	CH	1	1.6	1.1	1.2	**0.001**	1.9	**0.003**	1.1	1	0.9	1.00E-04	27
*mutB*	PFREUD_07650	ylmalonyl-CoA mutase large subunit	CH	1.3	1.3	1.2	1	0.176			0.7	1.3	1	4.30E-04	16
*mutA*	PFREUD_07660	ylmalonyl-CoA mutase small subunit	CH	1.3	1.2	1.2	0.6	0.525			1	1	0.8	0.005	18
NC	PFREUD_10590	ylmalonyl-CoA epimerase	CH	1	2.3	1.3	−0.1	0.146			0.8	1.1	0.8	0.027	48
*pgm1*	PFREUD_10610	oglucomutase	CH	2.1	3.4	3.4	3.4	**5.10E-05**	3.9	**0.01**	0.8	0.7	1.1	1.30E-04	29
*lpd*	PFREUD_10890	ihydrolipoyl dehydrogenase	CH	0.5	−1.5	−1.4	−2.1	**0.018**			0.3	0.7	0.5	0.002	34
*ldh2*	PFREUD_12840	tate dehydrogenase	CH	0.2	1.2	1.6	1	0.099	1.9	**0.012**					
*glpB*	PFREUD_12980	aerobic glycerol-3-phosphate drogenase subunit B	CH	0.2	−1.4	−0.7	−1	0.221			0.7	0.9	0.8	0.031	38
*gap*	PFREUD_15130	yceraldehyde-3-P dehydrogenase	CH	0.2	2.3	1.6	0.5	0.075			−0.9	−0.7	−0.9	0.004	4
*glgC*	PFREUD_16180	ose-1-P adenylyltransferase	CH	0.8	2.6	2	1.1	**0.011**	3	**1.60E-04**					
*fumC*	PFREUD_16300	umarate hydratase	CH	1	2	1.4	0.1	0.145	2.6	**0.014**	0.8	1	1.2	0.007	35
*eno1*	PFREUD_17320	se 1	CH	0.7	2.7	1.7	0.9	0.053	1.5	**0.008**	0.7	0.4	0.8	0.001	37
*mmdA*	PFREUD_18860	ylmalonyl-CoA carboxytransferase 12S	CH	0.3	−0.1	−0.1	−0.4	0.593			0.6	0.4	0.7	0.013	25
NC	PFREUD_18870	ylmalonyl-CoA carboxytransferase 5S t	CH	0.2	0.1	0	0	0.995			0	0.6	0.9	0.02	31
*iolA*	PFREUD_19100	yo-inositol catabolism protein	CH	0.2	0.5	0	−0.1	0.163			0.9	1	1	3.90E-05	30
*ptsI*	PFREUD_19470	nzyme I	CH	0.9	3.5	2.7	2.3	**4.50E-04**	5.5	**0.001**					
*tkt*	PFREUD_22360	olase	CH	0.1	2.9	2.2	0.9	**0.005**			0.6	0.5	0.6	0.031	15
*fba2*	PFREUD_23890	ose-bisphosphate aldolase	CH	2.3	3.8	3.4	3	**3.00E-05**	4.9	**2.30E-04**					

a Spot number (see Table S4 and Fig. 2).

Bold characters indicate genes that are differentially expressed according to microarray experiments (*P*<0.05 and |fold change|>1 for at least one sampling time) and RT-qPCR (*P*<0.05) experiments.

### Changes in expression according to gene functional category

**Figure 3:**
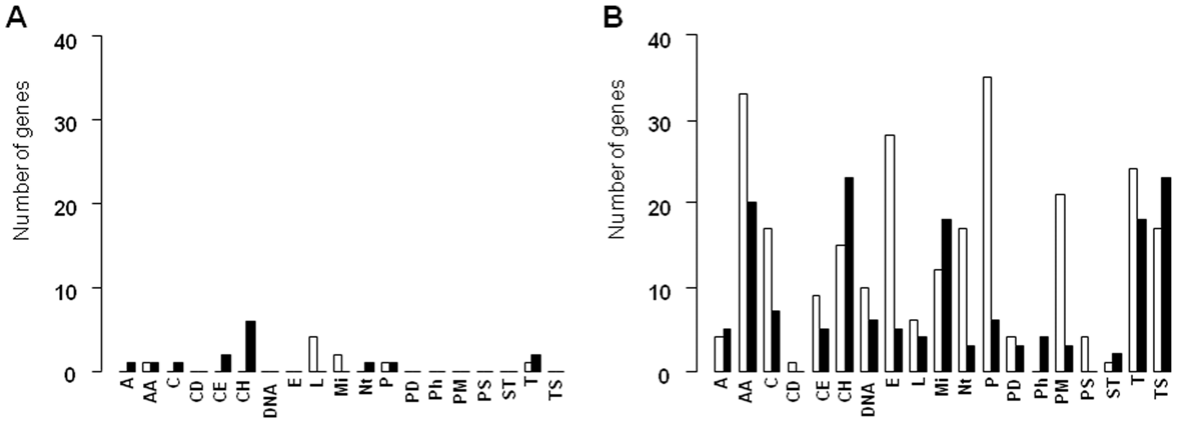
Number of differentially expressed genes (*P*<0.05 and |fold change|>1) after 40 h at 30°C (**A**, 24 genes) and after 3 days at 4°C (**B**, 413 genes), in comparison with genes expressed at the reference time 20 h. Down-regulated (white histogram) or up-regulated (black histogram) genes with known functions are presented according to their metabolic category: A, adaptation to atypical conditions; AA, transport and metabolism of amino acids; C, metabolism of coenzymes and prosthetic groups; CD, cell division; CE, cell envelop; CH, transport and metabolism of carbohydrates; DNA, DNA metabolism; E, energy metabolism; L, lipid metabolism; Mi, miscellaneous; Nt, transport and metabolism of nucleotides; P, protein synthesis; PD, protein degradation; Ph, metabolism of phosphate; PM, protein modification and folding; PS, protein secretion; ST, signal transduction; T, transport of peptides and inorganic ions; TS, transcription.

Genes were classified in 19 functional categories (Table S3). The number of up- or down-regulated DE genes in each category is illustrated in Figure 3. Only 24 genes (1% of the whole genome) were differentially expressed at the end of incubation at 30°C (40 h), in comparison to the reference time (Fig. 3A), whereas 413 genes were differentially expressed at 4°C (3 days) (Fig. 3B).

At 4°C, most of the DE genes implicated in functions related to the general cell machinery were down-regulated, including those related to the metabolism of nucleotides (Nt), co-enzymes and prosthetic groups (C), energy (E), and proteins (PD, P, PM) (Fig. 3B). In contrast, similar numbers of down and up-regulated genes were observed in some gene categories, like adaptation to atypical conditions (A), DNA metabolism (D), transcription (TS) and transport and metabolism of amino acids (AA), carbohydrates (CH), and inorganic ions (T). Lastly, the category dedicated to phosphate metabolism exhibited exclusively up-regulated genes at 4°C (Fig. 3B). These general trends summarize the strategies of adaptation and survival of *P. freudenreichii* CIRM-BIA1^T^ at 4°C, which are discussed in detail in the following sections.

### General cell machinery slowing down

At 4°C, CIRM-BIA1^T^ growth and general metabolism slowed down. The growth curve flattened out at an OD of 3, i.e. 1.9±0.5.10^9^ colony forming unit/ml (cfu/ml). Accordingly, expression of the *fts*X gene involved in cell-division was reduced at 4°C, as revealed by both microarray and RT-qPCR results (fold change of −4.2 and −4.3, respectively) (Table 1). Lactate consumption and acetate and propionate production rates also decreased by a factor of 19, 29 and 25, respectively (Table 5). However, cells did not show any sign of carbon starvation since *cst*A, coding for a carbon-starvation protein which was transiently up-regulated at 40 h in response to the low lactate content, was then down-regulated after lactate addition at 4°C (Table 2).

**Table 5:** Indicators of *P. freudenreichii* CIRM-BIA1^T^ strain metabolism during a 40 h-incubation at 30°C followed by 9 days at 4°C.

Indicators of metabolism	At 30°C (from 20 to 40 h)	At 4°C	Ratio 30°C/4°C
**Metabolite consumption and production rate (mM/day)**			
Lactate consumption	126.8±0.4	6.7±0.4	19
Propionate production	89.8±5.3	3.6±0.8	25
Acetate production	41.1±1.6	1.4±0.2	29
Succinate production	2.51±0.31	ND^b^	-
Methylbutanoate production^a^	0. 043±0.007	0.016±0.0002	3
**Molar ratio**			
Propionate produced/acetate produced	2.2±0.2	2.0±0.1	1.1
Propionate produced/lactate consumed (mol propionate/3 mol lactate)	2.12±0.12	1.61±0.37^c^	1.32
Acetate produced/lactate consumed (mol acetate/3 mol lactate)	0.97±0.03	0.81±0.14^d^	1.2

a Methylbutanoate is sum of 2-methylbutanoate and 3-methylbutanoate.

b Not detected.

c Values at 30°C and 4°C significantly differed at *P* = 0.09.

d Values at 30°C and 4°C significantly differed at P = 0.12.

Values are means and standard deviations of three independent experiments.

More than 60% of the DE genes were down-regulated at 4°C. At cold temperatures, many bacteria also exhibit a general slowing down, like for example *Lc. lactis* in model cheeses when placed at 12°C for seven days [7]. In particular, in the present study, *P. freudenreichii* CIRM-BIA1^T^ down-regulated most of the DE genes involved in energetic metabolism (29/34). This included genes encoding ATP synthases (*atp*ABCDEFGH) and several oxidoreductases of the electron transport chain (*nuo*ABCDEFG, *nuo*JKL, *nuo*M, *cyd*AB, and *sdh*AC, Table S2). Genes encoding the elongation factor (*tuf*) and ribosomal proteins were also down-regulated at 4°C, as previously observed in *L. lactis* [7]. Proteomics also partly confirmed CIRM-BIA1^T^ metabolism slowing down. For example, NuoD and the elongation factor were some of the identified proteins which volume decreased at 4°C (Table 1, Table S4 and Fig. 2). Cells also diminished protein synthesis at 4°C, since 87% (33/38) of the DE genes for this category were down-regulated (Table S2).

Concerning amino acid synthesis pathways, all the DE genes were down-regulated at 4°C, except for Arg synthesis (Table S2). For example, genes coding for proteins of the branched-chain amino acid ABC transporter (*liv*FG, *brae*, *brad*, *yda*O, fold change ranging from −1.3 to −3.3), and for Met, Glu, Trp, Lys Ile, and Val synthesis were down-regulated at 4°C (Table S2).

Concerning carbohydrate metabolism, genes coding for enzymes of the TCA cycle (*icd* and *acn*) and components of the pyruvate dehydrogenase (*ace*E and *lpd*) were also down-regulated at 4°C.

### General cold stress response

When cultures were placed at 4°C, *P. freudenreichii* CIRM-BIA1^T^ had to face a cold stress and significantly up- or down-regulated stress protein-coding genes. Cells up-regulated genes coding for one stress-response transcriptional regulator protein and two cold shock proteins (*psp*C, *csp*A and *csp*B, fold change of +2.6, +2.2 and +2.6, respectively) (Table 2, Table S2). Cold shock proteins are essential for the cells to resume growth in the cold and are synthesized in many bacteria [15]. These proteins, usually transiently expressed and detected during the acclimation phase, act as chaperones [16] and could constitute an asset for *P. freudenreichii* to maintain its metabolic activity in the cold. *P. freudenreichii* CIRM-BIA1^T^ also down-regulated several chaperone and heat shock protein-coding genes (*gro*SL and *dna*KJ operons, *hsp*20, *clp*B, *grp*B), with fold change values ranging from −1.3 to −5.2 over the whole period at 4°C (Table 3, Table S2). The spot volumes of DnaK2, GroS1, ClpB2 and Hsp20 1 proteins also decreased at 4°C (Table 3, Table S4 and Fig. 2). These proteins are known to be induced by several other stresses in *P. freudenreichii* [17], [18], and, in contrast, to be repressed upon cold shock in different bacterial species [19], [20], as observed for CIRM-BIA1^T^.

The up-regulation of two genes encoding DEAD-box RNA helicases, PFREUD_04260 and PFREUD_13460, with fold change values of +1.0 and +1.9 respectively (Table S2), were other indicators of the cold stress response in *P. freudenreichii* CIRM-BIA1^T^. The up-regulation of these helicases has previously been observed in *L. monocytogenes* [16] and many other microorganisms in the cold [21]. They help to relieve secondary structures formed by RNA at low temperatures, thus facilitating translation, and could contribute to the ability of *P. freudenreichii* cells to withstand cheese ripening conditions [21].

The maintenance of membrane fluidity is another absolute priority for bacterial survival in the cold [22]. *P. freudenreichii* membrane contains a large majority of odd numbered, iso- and anteiso-form branched-chain fatty acids [23], [24]. In the cold, *P. freudenreichii* changes its membrane fatty acid profiles in reducing the presence of iso-form fatty acids in favor of anteiso-form fatty acids [24]. Branched-chain fatty acids are synthesized thanks to the activity of a branched-chain alpha-keto acid dehydrogenase, encoded by the *bkd* operon. In the present study, the genes of the *bkd* operon were either non-differentially expressed or were down-regulated (*bkd*A2, fold change = −3.2, Table 2). Similarly, in *L. monocytogenes*, another bacterial species containing branched-chain fatty acids, the transcription of the *bkd* operon is not affected at 4°C [16]. This suggests that the major cold regulation point in CIRM-BIA1^T^ is found downstream of the *bkd* operon in the branched-chain fatty acid synthesis pathway, as in *L. monocytogenes*.

In addition, the genes involved in Arg transport and synthesis (*arg*BCDEFG, *arg*JK) were up-regulated at 4°C (Table 2, Table S2). This result was unexpected because more than 2 mM of free Arg were still present in the culture medium at the end of incubation (Table S1). Whether the up-regulation of these genes resulted or not in the accumulation of intracellular Arg, and its role for *P. freudenreichii* remains hypothetic. In many bacteria, Arg can be used as an alternative energy source and to control intracellular pH [25].

### Long-term survival mechanism and rerouting of carbon metabolism

*P. freudenreichii* CIRM-BIA1^T^ kept a high viability over the whole period at 4°C, with 90% of the viable cells enumerated at 3 days being still cultivable at 9 days (1.7±0.3.10^9^ cfu/ml). Cells had to find other ways to get energetic supplies, since genes of the electron transport chain and ATP synthesis were down-regulated at 4°C. *P. freudenreichii* is known to accumulate inorganic polyphosphates (polyP) as an energy reserve during its growth on lactate [26], which are degraded by inorganic pyrophosphatases [27]. In the present study, an inorganic pyrophosphatase-coding gene, *ppa*, was especially up-regulated at 4°C (RT-qPCR fold change = +3.3, Table 3). PolyP could thus be used to regenerate ATP from ADP in CIRM-BIA1^T^, as previously described in other bacteria [28].

**Figure 4:**
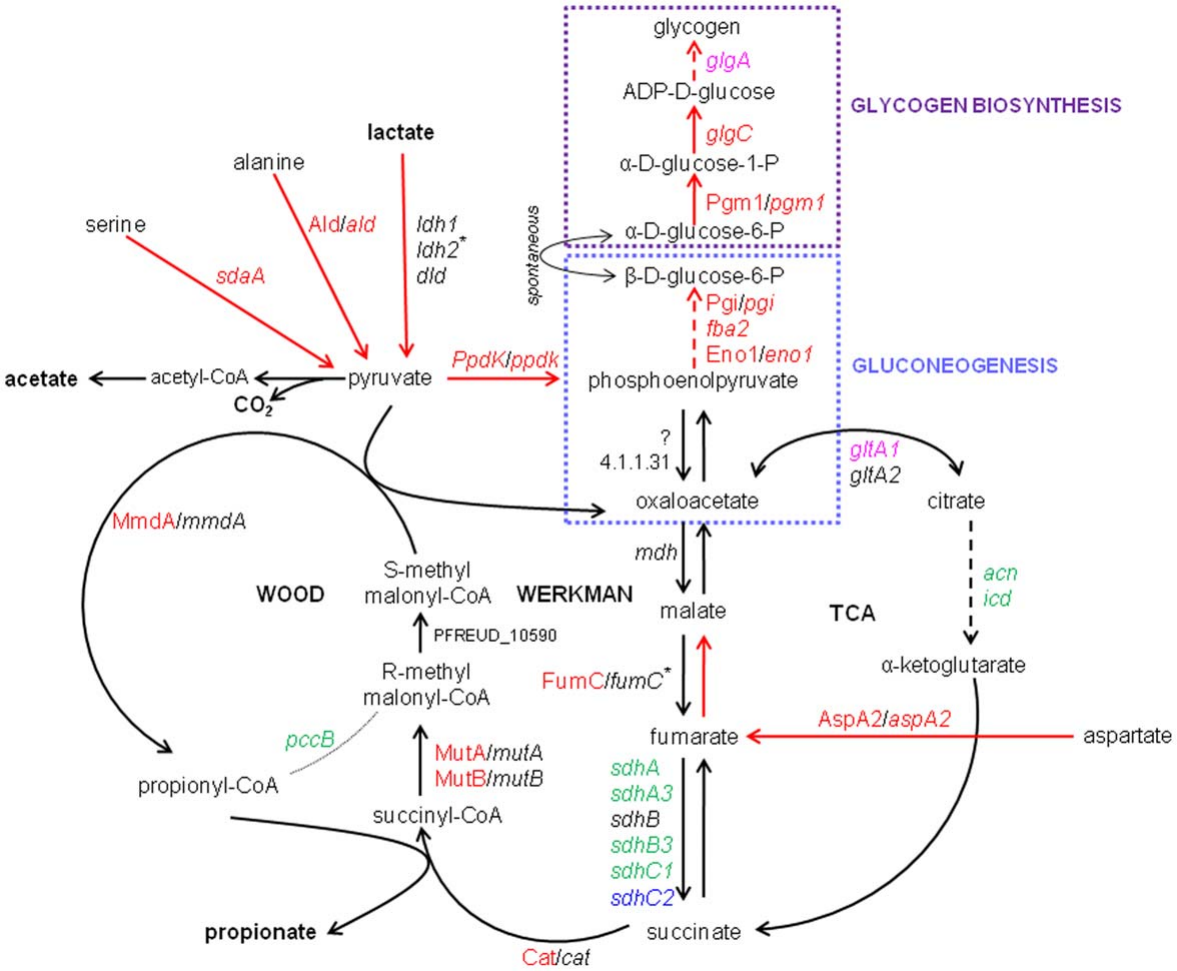
Schematic representation of carbon metabolism in *P. freudenreichii* CIRM-BIA1^T^ during storage at 4°C and relevant for this study. Protein and gene names are written in plain and in italic fonts, respectively. The color indicates the change in expression at 4°C, compared to reference time (exponential growth at 30°C, 20 h): in red, genes up-regulated at 4°C using microarray and RT-qPCR data and protein present at larger amounts; in pink, genes up-regulated at 4°C using microarray results only; in blue, genes down-regulated at 4°C using microarray and RT-qPCR data; in green, genes up-regulated at 4°C using microarray data only; in black, genes not being differentially expressed at 4°C using microarray data. * genes up-regulated using RT-qPCR data, but not significantly affected using microarray experiments. Red lines emphasize the metabolic pathways that seem to be favored at 4°C in *P. freudenreichii* considering the proteomic and transcriptomic results. Plain lines are used to symbolize one reaction, dotted lines several reactions (intermediary products not detailed). Gene and protein abbreviations are as follow: *acn* (aconitase), Ald/*ald* (alanine dehydrogenase), AspA2/*aspA2* (aspartate ammonia-lyase), Cat/*cat* (coenzyme A transferase), *dld* (D-lactate dehydrogenase), Eno1/e*no1* (enolase 1), *fba2* (fructose-bisphosphate aldolase class I), FumC/*fumC* (fumarate hydratase class-II), *glgA* (glycosyltransferase), *glgC* (glucose-1-P adenylyltransferase), *gltA1,2* (citrate synthases), *icd* (putative isocitrate/isopropylmalate dehydrogenase), *ldh* (L-lactate dehydrogenase), *mdh* (malate dehydrogenase), MmdA/*mmdA* (methylmalonyl-CoA carboxytransferase 12S subunit), MutA/*mutA* (methylmalonyl-CoA mutase small subunit), MutB/*mutB* (methylmalonyl-CoA mutase large subunit), *pccB* (propionyl-CoA carboxylase β-chain), Pgi/*pgi* (glucose-6-P isomerase), Pgm1/*pgm1* (phosphoglucomutase), PpdK/*ppdk* (pyruvate phosphate dikinase), *ptsI* (PTS enzyme I), *sdaA* (L-serine dehydratase), *sdhA* (succinate dehydrogenase, subunit A), *sdhA3* (succinate dehydrogenase, flavoprotein subunit), *sdhB* (succinate dehydrogenase, subunit B), *sdhB3* (succinate dehydrogenase), *sdhC1* (succinate dehydrogenase, subunit C), *sdhC2* (succinate dehydrogenase, cytochrome B-558 subunit), ?: undefined protein (probable phosphoenolpyruvic carboxylase).

A rerouting of carbon metabolism in *P. freudenreichii* CIRM-BIA1^T^ in the cold was also observed, as summarized in Figure 4 and discussed below. The formation of pyruvate from various sources was actively up-regulated. The expression of lactate dehydrogenases, catalyzing the oxidation of lactate into pyruvate, was either maintained at 4°C (*ldh*1 and *dld* genes), or significantly up-regulated (*ldh*2 gene, RT-qPCR fold change = +1.9) (Table 4). In agreement, lactate was constantly consumed at 4°C, with a total of 55.3±3.8 mM consumed over the period at 4°C (Fig. 1A). Moreover, the reactions of Ala and Ser conversion into pyruvate were up-regulated. Indeed, the transport of Ala and Ser into the cell, encoded by *cyc*A2, was up-regulated, as well as the conversion of Ala and Ser into pyruvate, encoded by *ald* and *sda*A genes respectively (fold change of +7.3 and +3.5, respectively) (Table 2, Table S2, Fig. 4). The Ald protein was also induced at 4°C (Table 2). However, the concentration of free Ala and Ser in culture supernatant decreased by only 0.15±0.06 and 0.18±0.04 mM, respectively, over the incubation period in the cold (Table S1). Intracellular Ala and Ser stocks may have also been converted into pyruvate. Propionate and acetate continued to be produced with the same propionate/acetate ratio compared to that observed at 30°C (Fig. 1A, Table 5). However, the yield of propionate and acetate produced per mole of lactate consumed tended to decrease at 4°C, compared to 30°C (Table 5). This result suggests that a fraction of the pyruvate produced from lactate was redirected towards other metabolic pathways at 4°C.

Our transcriptomic and proteomic data suggest that *P. freudenreichii* CIRM-BIA1^T^ metabolism was redirected towards glycogen synthesis through gluconeogenesis (Fig. 4). Hence, pyruvate phosphate dikinase, catalyzing the conversion of pyruvate into phosphoenolpyruvate (PEP), was over-expressed at 4°C (Table 4). PEP could have been converted to α-D-glucose-6-P via gluconeogenesis, as suggested by the up-regulation at 4°C of *eno*1, *fba*2, *pgi* genes and some of the corresponding proteins (Eno1 and Pgi) (Fig. 4, Table 4). Gluconeogenesis was previously reported in resting cells of the same *P. freudenreichii* strain incubated in the presence of labeled pyruvate [29]. In our study, the final product of gluconeogenesis, α-D-glucose-6-P, could have been converted into glycogen, as suggested by the up-regulation of *glg*C (fold change = +2.6) encoding ADP-glucose synthase, the main regulation enzyme of glycogen synthesis [30], and of two other enzymes of glycogen synthesis pathway, at gene and/or protein level (Fig. 4, Table 4). Glycogen is an α-1,4 linked, α-1,6 branched glucose polymer used for long-term energy storage. Its metabolism is highly interconnected with a wide range of cellular processes and tightly adjusted to the nutritional and energetic status of the cell [31]. *P. freudenreichii* is able to synthesize and accumulate intracellular glycogen, although it is not able to degrade extracellular glycogen [32]. Storing glycogen should then be an asset for *P. freudenreichii* contributing to its aptitude to adapt, resist and survive to stressful conditions such as the low temperature during cheese ripening.

In the present study, Asp may also have been used as an additional carbon source for glycogen synthesis. The aspartate ammonia-lyase (AspA2), catalyzing the conversion of aspartate into fumarate, was over-expressed at 4°C at both transcriptomic and proteomic levels (Table 2). Succinate was no more excreted at 4°C (Fig. 1B), and, in agreement, the two copies of the succinate dehydrogenase-coding genes were down-regulated. In contrast, fumarate hydratase was over-expressed at gene (*fum*C, RT-qPCR fold change of +2.6) and protein levels (Table 4, Fig. 4). Altogether, these results suggest that Asp could have been converted to fumarate, malate, and then to pyruvate and/or PEP to be used as an additional substrate for gluconeogenesis. Only intracellular Asp would have been used for glycogen synthesis, since the culture medium was depleted in free Asp and Asn at 4°C and that the total Asp content in the medium did not significantly decrease during incubation at 4°C (Fig. 1B, Table S1).

### Aroma compound production in the cold

*P. freudenreichii* has a key role in the formation of Swiss cheese flavor by producing a variety of flavor compounds from three main origins: propionate from lactate fermentation, methylbutanoic acids from the conversion of Ile and Leu, and free fatty acids from milk fat hydrolysis by lipolytic carboxylic ester hydrolases [32].

Most of the genes involved in Wood-Werkman cycle converting pyruvate to propionate were not differentially expressed in conditions mimicking cold room ripening of cheeses. The Wood-Werkman cycle seemed to be maintained at 4°C, as indicated by the increase in spot volume at 4°C of FumC, Cat, MmdA, MutA and MutB proteins (Table 4, Fig. 4 and Table S4). Accordingly, propionate continued to be produced, as stated above, but its formation rate decreased by a factor of 25 (Table 5).

The production of methylbutanoic acids, in contrast, decreased by a factor of only 3 at 4°C (Fig. 1C, Table 5). The formation of methylbutanoic acids from Leu and Ile is catalyzed by genes encoding branched-chain amino acid aminotransferases (*pab*C and *ilv*E) and genes of the *bkd* operon [12]. As already mentioned, these genes were not differentially expressed at 4°C, except for the *bkd*A2 gene.

Ten genes coding for (putative) carboxylic ester hydrolases were previously identified in CIRM-BIA1^T^ genome [33] and could be involved in fat hydrolysis or ester synthesis. Interestingly, nine of these 10 genes were not differently expressed at 4°C, whereas the remaining one (*pf*379) was significantly up-regulated (fold change = +1, Table S2). These results coincide with the observation that milk fat hydrolysis and ester synthesis are maintained in cheese at cold temperatures [6].

### Conclusions

This study brought new insights into the metabolism of *P. freudenreichii* CIRM-BIA1^T^ in conditions mimicking cheese transfer from the warm room to the cold room. It highlights in particular how this strain re-directs some metabolic pathways to accumulate carbon storage compounds and to use alternative energy storage compounds like polyP, contributing to its long-term survival. Further work is in progress to determine if other *P. freudenreichii* strains exhibit similar metabolic adaptations in the cold, compared to CIRM-BIA1^T^, or if they use additional adaptive strategies. Since some strains of *P. freudenreichii* are now considered for their probiotic properties [34], our results could also be of interest to develop dairy products containing probiotic strains keeping high viable populations during their storage at a low temperature.

## Materials and Methods

### Strain and culture conditions

The sequenced *P. freudenreichii* CIRM-BIA1^T^ strain [35] was grown in YEL broth [10] under air atmosphere without agitation. Growth was monitored by optical density measurements at 650 nm (OD_650_). Cultures were incubated at 30°C until they reached an OD_650_ of about 2±0.11 (at 40 h), i.e. before the exhaustion of the main carbon source (lactate). Then, cultures were incubated for a further 9 days at 4°C. Lactate (132 mM/ml, Panreac, Lyon, France) was added after 24 h incubation at 4°C in order to mimic the lactate availability during cheese ripening, thus preventing carbon starvation. Cultures were harvested at five stages: in the middle of the exponential growth phase at 30°C (20 h, OD_650_≈0.5), at the end of exponential growth phase at 30°C (40 h, OD_650_≈2), and after 3, 6 and 9 days of incubation at 4°C. After 3 and 9 days at 4°C, the bacterial concentrations were determined by CFU counting on YEL agar incubated at 30°C for 5 days, in anaerobic conditions.

Experiments were performed in triplicate as independent cultures.

### RNA extraction

Total RNA extraction was adapted from Deutsch et al [36]. Briefly, at each sampling time, cells were harvested (8000×g, 10 min, room temperature or 4°C regarding the sampling time). Cell pellets were suspended in 100 µl of TE buffer 1× (Qiagen, Courtaboeuf, France) containing lysozyme (20 mg/ml), and incubated for 15 min at 24 °C before addition of 350 µl RLT buffer (from the Qiagen RNeasy minikit). 50 mg of zirconium beads and 100 µl of sodium dodecyl sulfate (SDS, 20%) were then added and cells were disrupted for 2×90 seconds at 30 Hz by using a Retsch MM301 high-speed mixer mill (Grosseron, France). RNA was extracted according to the instructions of the RNeasy kit. Then DNase treatments of the RNA samples were performed using a DNA-free kit (Ambion, Texas, USA) according to the manufacturer's instructions. RNA quantification and contamination by proteins were assessed using a NanoDrop ND-1000 spectrophotometer (NanoDrop Technologies, Inc., Rockland, DE). RNA quality was evaluated using an Agilent 2100 bioanalyzer (Agilent Technologies, Santa Clara, CA) and the RIN value was calculated as previously described [37]. All of the RNA samples had a RIN value greater than 8.4, indicating a good RNA integrity. The absence of genomic DNA was confirmed by quantitative PCR (qPCR).

### Transcriptomic analyses and statistical analysis

Microarray design, manufacture, synthesis and image scanning were provided by Source BioScience imaGenes (Berlin, Germany). Briefly, *P. freudenreichii* CIRM-BIA1^T^ microarrays were designed as 8×15K (i.e. 8 samples per slide with 15000 oligos targeting each sample) custom microarrays including all ORFs and intergenic regions from the whole genome of *P. freudenreichii* CIRM-BIA1^T^ strain. So, the number of oligos targeting CIRM-BIA1^T^ strain genome ranged from 1 to 5, with a mean of 4 oligos per gene or intergenic region. The complete microarray design is available in NCBIs GEO platform under accession number GPL13959. At each sampling time, hybridizations consisted in three biological replicates. A technical replicate was also performed for the reference time at 20 h. Cyanine-3 (Cy3) labeled cRNA was prepared from (50–100 ng) RNA using the Low Input Quick Amp Labeling Kit (Agilent Technologies) according to the manufacturer's instructions, followed by RNAeasy column purification (Qiagen). Dye incorporation and cRNA yield were checked with the NanoDrop ND-1000 Spectrophotometer. 600 ng of Cy3-labelled cRNA (specific activity >10.0 pmol Cy3/ug cRNA) was fragmented (60°C, 30 min) in a reaction volume of 25 µl containing 1× Agilent fragmentation buffer and 2× Agilent blocking agent following the manufacturer's instructions. On completion of the fragmentation reaction, 25 µl of 2× Hi-RPM hybridization buffer (Agilent Technologies) was added to the fragmentation mixture and hybridized to Agilent specific 8×15 K microarray for 17 h at 65°C in a rotating Agilent hybridization oven. After hybridization, microarrays were washed for 1 min at room temperature with GE Wash Buffer 1 (Agilent Technologies), then for 1 min with 37°C GE Wash buffer 2 (Agilent Technologies), and finally for 1 min at room temperature with acetonitrile. Slides were scanned immediately after washing on the Agilent DNA Microarray Scanner (G2565CA) using one color scan setting for 8×15 K array slides (Scan Area 61×21.6 mm, Scan resolution 5 µM, Dye channel set to Green and Green PMT set to 100%). The scanned images were analyzed with Feature Extraction Software 9.1 (Agilent Technologies) using default parameters. Normalization and microarray analysis were performed using limma package [38] of the R software (www.R-project.org). All intensities were expressed as log_2_ of the normalized signal intensities. Differential analysis was performed using empirical Bayes fitting in limma. Raw *P*-values were adjusted for multiple comparisons by the Benjamini-Hochberg procedure [39] which controls the False Discovery Rate. We considered genes with an adjusted *P*-value<0.05 to be DE. Moreover, expression ratios, indicating the fold change in expression between two samples, were calculated using 20 h sample as a reference. Only genes with a **|**fold change (log_2_)**|** greater than 1 (standing for an increase or decrease in expression of at least 2 times) were considered as biologically significant. Genes fitting both *P*-value and fold change criteria were retained. The data presented in this publication have been deposited in NCBIs Gene Expression Omnibus (GEO, http://www.ncbi.nlm.nih.gov/geo/) and are accessible though GEO Series accession number GSE30841.

The DE genes were also classified according to their temporal expression profiles using the quadratic regression method proposed by Liu et al. [14].

### Reverse transcription quantitative PCR

In order to confirm the results from the transcriptomic analyses, reverse transcription quantitative PCR (RT-qPCR) experiments were carried out. Transcripts were quantified at two specific times, at the exponential phase of growth (reference time 20 h) and after a 3-day incubation at 4°C. Twenty-eight genes observed as DE using microarray experiments were chosen. Primers were designed using Primer3Plus software [40] with default parameters except for the difference of melting temperature between the forward and reverse primers set to less than 1°C. The primer sequences for the tested genes are detailed in Table 6. cDNA synthesis, quantitative PCR, and cycle thresholds (Ct) were performed according to Falentin et al. [1]. Briefly, cDNA was synthesized using a qScript™ cDNA synthesis kit (Quanta BioSciences, Maryland, USA). Amplification by qPCR was performed with a 15 µl final volume mixture containing 5 µl of a cDNA template dilution of 1 in 40, 0.5 µM of each primer and 1× IQ™ SYBR Green Supermix (BioRad, California, USA), in an Opticon 2 real-time PCR detector (Biorad). Amplification cycles consisted in an initial step at 95°C for 5 min followed by 40 cycles of 95°C for 30 s, 60°C for 30 s, and 72°C for 1 min. Amplicon denaturation step of 0.5°C/min from 65°C to 90°C was performed to verify amplification specificity and determine amplicon melting temperature. Five genes that were not DE in microarray experiments (PFREUD_18870, *gyr*B1, 16S, *pf*279, *gtf*F) were chosen as possible internal standards for RT-qPCR normalization. The stability of mRNA expression of these genes was checked by using the geNorm VBA applet for Microsoft Excel [41]. Thus, PFREUD_18870, *pf*279 and *gtf*F genes were chosen as internal standard for normalization. The delta-delta-Ct method of geNorm was used to determine the normalized expression level of genes of interest (http://medgen.ugent.be/genorm/). An ANOVA was performed and changes in gene expression between the two conditions with a *P*-value<0.05 were considered as significant. To facilitate comparison with transcriptomic data, the results of RT-qPCR were expressed as fold change in log_2_.

**Table 6:** Primers used for reverse transcription quantitative PCR (RT-qPCR).

Gene		Primer sequences	
Name	Locus tag	Forward	Reverse
*ald*	PFREUD_00370	TGTTCACCTACCTGCACCTG	GAGCTCAACCGTCTCGTAGG
*argG*	PFREUD_01460	GACCATCAACGGCAAGAAGT	TCGTAGGCGATGAACAACAG
*argJ*	PFREUD_13980	GCATGTCCACGAACGACTC	AACTGGTGTCCCAGGTTGAG
*aspA2*	PFREUD_16330	TCCACGCGTACAGAAGAAGA	CCGGACATCTGGAAGTTCTC
*bkdA2*	PFREUD_02200	TCGCATTTCGTACACACTCC	TTCGGCTCGAAGAAGATCAC
*cspB*	PFREUD_18210	GCGATGATGGAGGATTTGAT	CGATCTTTGAGGCCTGACC
*cstA*	PFREUD_16500	CCTACACACCGGAGGAAGAG	GGACCGTCCATTTGTTATGC
*dps*	PFREUD_02870	GACCTGGTCGTGAAGTCGAT	GCTTGTTGATCAGCTCATGG
*eno1*	PFREUD_17320	TACGAGTTCGAGGGCAAGTC	AAACCAGCGGGTAGTCATTG
*fba2*	PFREUD_23890	GATCAGAAGGACCGGATGG	CATTGTCGTAGGCATCATCG
*feoA*	PFREUD_19660	AGACCGTGGGTTCCAAGAT	GACTCGTCAGCCTCTTCGTC
*feoB*	PFREUD_19650	GCGGTGATGTTCCTGTTCTT	TAGCCGAAGAAGGTGTCGAT
*ftsX*	PFREUD_09600	AGAACTACAAGGGCGTGGTG	CCGTTGAGGACCTTGAAGAA
*fumC*	PFREUD_16300	CCCAACCTCGAGAAGATCAA	ATCTTCGAGGCCTTGTCGTA
*glgC*	PFREUD_16180	CGTGCTCACGCAGTACAAGT	GGTACGGGCGTCACATAGTT
*groeS1*	PFREUD_06470	CAATGCGGTGCTTCTTCTC	GGCAAGGACTCCGATGTG
*gtfF*	PFREUD_19370	TGGTGACGCCGAAGGACTC	GCAGCACGAGCAGGAACAC
*gyrB1*	PFREUD_12820	TTGCAGGGCAGCGACCACTT	GACCAGCGCACGGCAACAT
*ldh2*	PFREUD_12840	GATGCCTCCACCAATGAGAT	CATAGTTCGTGGAGCCCTTG
*livG*	PFREUD_10850	TCTGCGAGCGTATCTACGTG	GGTATGCCTCGATCACCTGT
*mraZ*	PFREUD_15590	ATCAATCGGGTTGAGGTGTG	GTTCATCTGGGCGAAGACAT
PFREUD_18300	PFREUD_18300	CACGGAGGAGGAGAAGATCA	GTACTTCCACAGCTGCACGA
PFREUD_20370	PFREUD_20370	ACCTGACCTTCGACATGACC	GGGGTACTGAGTGGAGGTGA
*pgi*	PFREUD_04290	GAGGCACTCAAGCCCTACAA	AGGTCGTGGGTCTTCTCGTA
*pgm1*	PFREUD_10610	CCGAGGTGGAGTTCTTCAGT	GACCAGCTTGGACGACAAC
*ppa*	PFREUD_23500	TGAAGTCGGTTGATGGTGAG	GTTCCAGCTCCTGCTTCATC
*ppdk*	PFREUD_03230	ATGCCTCGATGAAGTCCTTG	GTGGTGACCGTAAAGCCATC
*pf279-F1*	PFREUD_04340	CGACTCCTACCAGCAGAAGC	CATTGTTTGACAAGGCCTGA
*pspC*	PFREUD_06710	GATCTGCTGGCTGTTGATCC	TCGTAGGGGTTGAAGTCCTG
*ptsI*	PFREUD_19470	CGAGATCAAGAACGACACCA	GGTTGAAATAGGCGTTCGAC
*sdaA*	PFREUD_18570	CGCGGAACTGCTCAATATCT	CACCTCGTCCTTCGAGTAGC
*sdhC2*	PFREUD_14320	CTGATGGCTCTCACCGGTAT	TCGTTCATGAGCAGCTTCAG
ND	PFREUD_18870	CATGGCTGAAAACAACAATTTG	TCTGCTTACCCTGGGAATG
*tuf*	PFREUD_05650	CGAACGAGTTCCACTGCGGGT	GCAACATCGGCACCATCGGAC
*tyrB*	PFREUD_09460	CGACATGGACTTCATCAACG	ATCGGTGCCATAGACATGGT

### Protein extraction

Protein extraction was performed as described by Jan et al. [42], with modifications. In brief, cells were harvested at four sampling times (20 h and 3, 6 and 9 days) by centrifugating 50 ml of culture (7000×g, 10 min, room temperature or 4°C regarding the sampling time) and washed in a wash buffer (50 mM Tris-HCl [pH 7.5], 150 mM NaCl, Panreac). Washed cells were then recovered in a protoplastization solution (25 mM Tris-HCl [pH 7.5], 0.5 M sucrose, 0.1 mg of lysozyme per ml, 1 mg of chloramphenicol per ml, 0.4 mM phenylmethylsulfonyl fluoride [PMSF]) and incubated for 30 min at 37°C prior to centrifugation (7000×g, room temperature, 10 min). The cell pellet was recovered in a lysis solution (50 mM Tris-HCl [pH 7.5], 0.3% SDS, 200 mM dithiothreitol (DTT), 0.4 mM PMSF, 1 mg/ml chloramphenicol) and sonicated on ice with a Vibra Cell sonicator (Bioblock Scientific, Illkirch, France) equipped with a tapered microtip (three bursts of 1 min at 1-min intervals, output of 2.5). The lysates were centrifuged (10000×g, 10 min, room temperature) and the supernatants were stored at −20°C until electrophoresis.

### Two-dimensional electrophoresis (2D electrophoresis) and image analysis

Proteins were precipitated (2D clean up kit, GE Healthcare) and rehydrated in 100 µl of Destreak rehydratation solution (GE Healthcare) containing 20% IPG buffer (pH 4/7, GE Healthcare). Isoelectric focusing was carried out using pH 4 to 7 Immobiline Dry Strips (18 cm, GE Healthcare) on a Multiphor II electrophoresis system (Amersham Biosciences). Second dimension SDS PAGE (14% monomers) was performed on an Ethan Dalt Twelve (GE Healthcare) according to a procedure previously described [42]. Analysis of the gel images was undertaken using Progenesis SameSpots software version 3.3 (Nonlinear Dynamics, Newcastle upon Tyne, United Kingdom). The gel images were aligned by automated calculation of alignment vectors after assigning 30–35 landmark vectors [43]. An ANOVA was performed and a threshold of *P*<0.05 was chosen to identify significant changes in spot volumes. Differentially expressed proteins also have to match a fold change criterion, expressed in log_2_, of at least 0.6.

### Nano-LC/ESI-MS-MS analysis

Protein identification was performed using tandem mass spectrometry (MS/MS) as previously described [44]. All mass spectrometry data were submitted to MASCOT v.2.2 software for search into two concatenated databases: (i) Swiss-Prot database (http://www.expasy.org) and (ii) a portion of the UniProtKB database corresponding to the *P. freudenreichii* taxonomic group. Fifty protein spots were excised and submitted to an in-gel tryptic digestion as previously described [18]. The alkylation of cysteine by iodoacetamide was specified as a fixed modification while the two following modifications were set as variable modifications: oxidation of methionine and deamidation of asparagine and glutamine residues. The peptide mass tolerance was set to 0.2 Da for MS 0.15 Da for MS/MS. For each peptide identified, a minimum of four peptides with MASCOT score corresponding to a *P*-value<0.05 or an Exponentially Modified Protein Abundance Index (emPAI) [45] greater than 0.4 were necessary for validation with a high degree of confidence. For the validation of peptides from the MASCOT search results, the 1.19.2 version of the IRMa software was used [46].

### Analysis of metabolites in culture supernatants

Free and total amino acids were analyzed with an amino acid analyzer (AlphaPlus series 2; Pharmacia, Uppsala, Sweden). Lactic, propionic, acetic, succinic, and pyruvic acids were quantified in culture supernatants by HPLC on an Aminex A-6 ion exchange column (Bio-Rad, Hercules, CA) at 55°C with 0.005 M H_2_SO_4_ as eluent at a flow rate of 0.4 ml.min^−1^. Both UV (210 nm) and refractometric detectors were used. Methylbutyric acid (sum of 2-methylbutyric and 3-methylbutyric acids) concentration was determined in culture supernatants after acidification by oxalic acid (final concentration: 0.03 mM) by gas chromatography, as previously described [12]. The rate of metabolites consumption or production was calculated i) at 30°C, between 20 h and 40 h, ii) at 4°C, by linear regression of values at lactate addition time point (64 h), at 3, 6 and 9 days. The differences between means from independent triplicate cultures for concentrations, ratios, and rates of metabolite consumption or production at the different sampling times were assessed by Student tests.

## Supporting Information

Figure S1 **Growth of** ***P. freudenreichii*** **CIRM-BIA1^T^ strain at 30°C monitored by optical density measurements (650 nm) in YEL medium containing 130 mM (plain line) or 260 mM of lactate (dotted line).**
(TIF)

Figure S2 **An illustration of the two main temporal expression patterns of** ***P. freudenreichii*** **CIRM-BIA1^T^ strain genes identified by the quadratic regression method proposed by Liu et al.** [**15**] **.** The black dots are the hybridization signals. The curve is the fitted regression pattern. Pattern and genes given as an example of the pattern are as follows **A**: example of a quadratic linear concave up regulated regression pattern (for PFREUD_06720 gene) displayed by 178 differentially expressed genes; **B**: example of a quadratic linear convex down regulated regression pattern (for *gro*EL1 gene) displayed by 272 differentially expressed genes.
(TIF)

Table S1 **Concentrations of total and free amino acids, and NH_3_ in** ***P. freudenreichii*** **CIRM-BIA1^T^ strain supernatant samples over time.**
(DOC)

Table S2 ***P. freudenreichii*** **CIRM-BIA1^T^ strain 565 differentially expressed genes according to microarray experiments (** ***P*** **<0.05, |fold-change|>1).**
(DOC)

Table S3 **Description of the metabolic function of genes grouped in each category.**
(DOC)

Table S4 **Proteins from** ***P. freudenreichii*** **CIRM-BIA1^T^ strain identified by tandem mass spectrometry.**
(DOC)
