# Correction: A Temporal -omic Study of *Propionibacterium freudenreichii* CIRM-BIA1^T^ Adaptation Strategies in Conditions Mimicking Cheese Ripening in the Cold

**DOI:** 10.1371/annotation/e0ff065d-a52d-44f2-8727-328393ed60b6

**Published:** 2012-04-05

**Authors:** Marion Dalmasso, Julie Aubert, Valérie Briard-Bion, Victoria Chuat, Stéphanie-Marie Deutsch, Sergine Even, Hélène Falentin, Gwénaël Jan, Julien Jardin, Marie-Bernadette Maillard, Sandrine Parayre, Michel Piot, Jarna Tanskanen, Anne Thierry

There was an error in affiliations 1, 3, and 4. The correct affiliations are:

1 INRA (French National Institute for Agricultural Research), UMR1253 Science et Technologie du Lait et de l'OEuf, Rennes, France,

3 INRA (French National Institute for Agricultural Research), CIRM-BIA, UMR1253 Science et Technologie du Lait et de l'OEuf, Rennes, France,

4 INRA (French National Institute for Agricultural Research), UMR518 Mathématiques et Informatique Appliquées, Paris, France 

