# Supplementary figures and images for: A Temporal -omic Study of Propionibacterium freudenreichii CIRM-BIA1T Adaptation Strategies in Conditions Mimicking Cheese Ripening in the Cold
Source: PLoS One. 2012 Jan 13;7(1):e29083. doi: 10.1371/journal.pone.0029083 (PMC3258244; doi:10.1371/journal.pone.0029083)

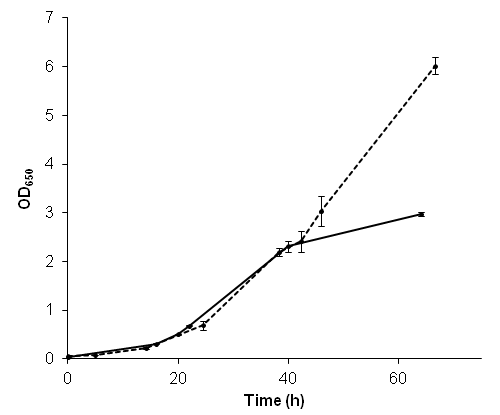

Supplement: Figure S1 — Growth of P. freudenreichii CIRM-BIA1T strain at 30°C monitored by optical density measurements (650 nm) in YEL medium containing 130 mM (plain line) or 260 mM of lactate (dotted line). (TIF) [file pone.0029083.s001.tif]

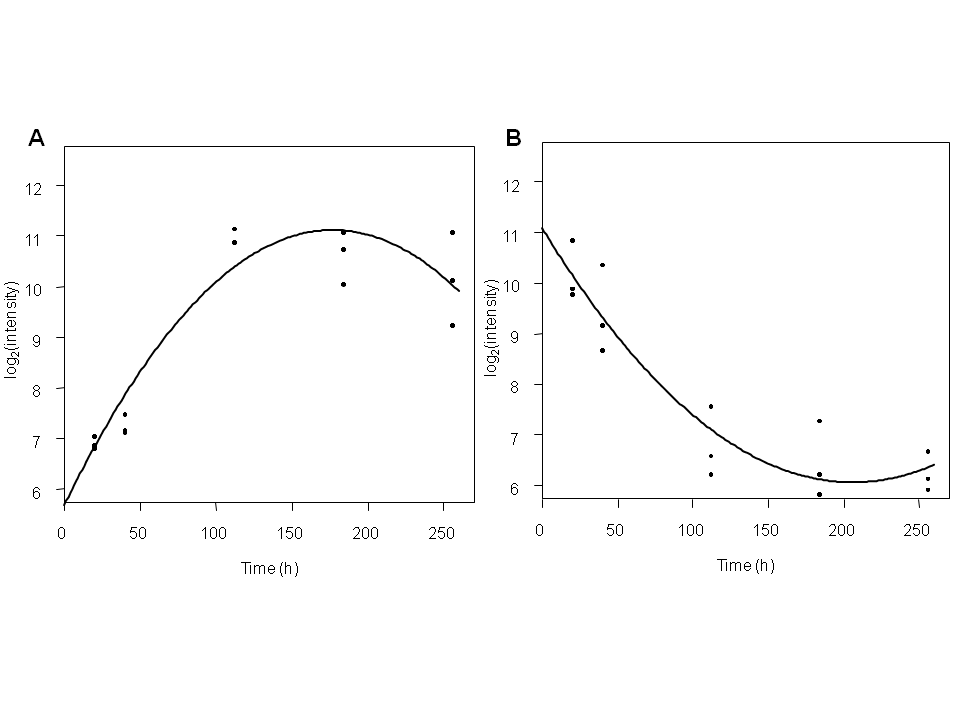

Supplement: Figure S2 — An illustration of the two main temporal expression patterns of P. freudenreichii CIRM-BIA1T strain genes identified by the quadratic regression method proposed by Liu et al. [15] . The black dots are the hybridization signals. The curve is the fitted regression pattern. Pattern and genes given as an example of the pattern are as follows A: example of a quadratic linear concave up regulated regression pattern (for PFREUD_06720 gene) displayed by 178 differentially expressed genes; B: example of a quadratic linear convex down regulated regression pattern (for groEL1 gene) displayed by 272 differentially expressed genes. (TIF) [file pone.0029083.s002.tif]
