# Supplementary material for: A Temporal -omic Study of Propionibacterium freudenreichii CIRM-BIA1T Adaptation Strategies in Conditions Mimicking Cheese Ripening in the Cold
Source: PLoS One. 2012 Jan 13;7(1):e29083. doi: 10.1371/journal.pone.0029083 (PMC3258244; doi:10.1371/journal.pone.0029083)
Supplement: Table S1 — Concentrations of total and free amino acids, and NH3 in P. freudenreichii CIRM-BIA1T strain supernatant samples over time. (DOC) [file pone.0029083.s003.doc]

**Table S1**: Concentrations of total and free amino acids, and NH_3_ in *P. freudenreichii* CIRM-BIA1^T^ strain supernatant samples over time.

| **Amino acid** | **Average concentration (mM) ± standard deviation^a^** | | | | | | | | | |
| --- | --- | --- | --- | --- | --- | --- | --- | --- | --- | --- |
|  | **Free amino acids** | | | | | | **Total amino acids** | | | |
|  | **0** | **20 h** | **40 h** | **3 days** | **6 days** | **9 days** | **0** | **20 h** | **40 h** | **9 days** |
| **Pser** | 0.33 ± 0.074 | 0.41 ± 0.058 | 0.36 ± 0.049 | 0.39 ± 0.061 | 0.38 ± 0.124 | 0.35 ± 0.022 | ND | ND | ND | ND |
| **Asp** | 1.28 ± 0.03 | 0.08 ± 0.03 | 0.03 ± 0.02 | 0.03 ± 0.02 | 0.02 ± 0.02 | 0.03 ± 0 | 8.71 ± 0.54 | 6.47 ± 0.64 | 5.69 ± 0.064 | 5.51 ± 0.54 |
| **Thr** | 2.12 ± 0.027 | 1.89 ± 0.026 | 2 ± 0.025 | 1.95 ± 0.008 | 1.86 ± 0.192 | 1.92 ± 0.021 | 5.85 ± 0.87 | 5.4 ± 0.47 | 6.04 ± 0.41 | 5.18 ± 0.86 |
| **Ser** | 2.64 ± 0.041 | 2.36 ± 0.015 | 2.12 ± 0.029 | 2.03 ± 0.02 | 1.91 ± 0.171 | 1.94 ± 0.011 | 6.92 ± 0.93 | 6.52 ± 0.87 | 6.81 ± 0.2 | 6.18 ± 0.76 |
| **Asn** | 1.76 ± 0.01 | 0.49 ± 0.04 | ND | ND | ND | ND | ND | ND | ND | ND |
| **Glu** | 5.12 ± 0.094 | 4.34 ± 0.025 | 4.07 ± 0.029 | 3.93 ± 0.048 | 3.74 ± 0.324 | 3.95 ± 0.08 | 19.42 ± 1.25 | 18.57 ± 1.9 | 18 ± 0.17 | 17.35 ± 1.82 |
| **Pro** | 1.03 ± 0.036 | 0.74 ± 0.017 | 1.11 ± 0.03 | 1.14 ± 0.063 | 1.14 ± 0.027 | 1.21 ± 0.022 | 9.23 ± 0.58 | 8.91 ± 0.89 | 8.88 ± 0.1 | 8.71 ± 0.89 |
| **Gly** | 2.11 ± 0.013 | 1.66 ± 0.064 | 1.36 ± 0.01 | 1.24 ± 0.016 | 1.1 ± 0.147 | 0.98 ± 0.029 | 6.1 ± 0.38 | 5.29 ± 0.3 | 5 ± 0.07 | 4.73 ± 0.51 |
| **Ala** | 4.7 ± 0.042 | 4.42 ± 0.095 | 4.48 ± 0.023 | 4.31 ± 0.048 | 4.14 ± 0.432 | 4.33 ± 0.041 | 7.77 ± 0.48 | 7.54 ± 0.75 | 7.26 ± 0.08 | 7.15 ± 0.73 |
| **Cit** | 0.05 ± 0.032 | 0.07 ± 0.038 | 0.05 ± 0.022 | 0.05 ± 0.026 | 0.05 ± 0.03 | 0.04 ± 0.0005 | ND | ND | ND | ND |
| **Val** | 3.12 ± 0.068 | 2.98 ± 0.052 | 3.28 ± 0.029 | 3.19 ± 0.022 | 3.08 ± 0.356 | 3.2 ± 0.028 | 7.82 ± 0.55 | 7.78 ± 0.8 | 7.3 ± 0.08 | 7.21 ± 0.58 |
| **Cys** | 0.23 ± 0.026 | 0.25 ± 0.053 | 0.18 ± 0.049 | 0.17 ± 0.034 | 0.2 ± 0.092 | 0.07 ± 0.004 | 0.67 ± 0.03 | 0.65 ± 0.03 | 0.63 ± 0.02 | 0.69 ± 0.13 |
| **Met** | 1.05 ± 0.016 | 0.99 ± 0.024 | 1.05 ± 0.024 | 1.02 ± 0.004 | 0.96 ± 0.113 | 0.99 ± 0.013 | 2.04 ± 0.03 | 2.03 ± 0.06 | 2.08 ± 0.04 | 1.88 ± 0.09 |
| **Ile** | 2.23 ± 0.039 | 2.03 ± 0.023 | 2.23 ± 0.012 | 2.18 ± 0.024 | 2.06 ± 0.222 | 2.16 ± 0.015 | 5.73 ± 0.41 | 5.64 ± 0.56 | 5.23 ± 0.08 | 5.16 ± 0.47 |
| **Leu** | 5.31 ± 0.053 | 5.09 ± 0.063 | 5.29 ± 0.059 | 5.17 ± 0.05 | 4.92 ± 0.492 | 5.26 ± 0.026 | 8.78 ± 0.55 | 8.73 ± 0.9 | 8.24 ± 0.06 | 8.25 ± 0.73 |
| **Tyr** | 0.46 ± 0.031 | 0.49 ± 0.007 | 0.56 ± 0.041 | 0.55 ± 0.037 | 0.53 ± 0.088 | 0.52 ± 0.006 | 1.47 ± 0.09 | 1.58 ± 0.14 | 1.51 ± 0.02 | 1.45 ± 0.14 |
| **Phe** | 2.62 ± 0.078 | 2.43 ± 0.036 | 2.51 ± 0.041 | 2.42 ± 0.035 | 2.29 ± 0.239 | 2.41 ± 0.04 | 3.88 ± 0.21 | 3.86 ± 0.37 | 3.74 ± 0.03 | 3.65 ± 0.34 |
| **Orn** | 0.14 ± 0.002 | 0.16 ± 0.01 | 0.16 ± 0.009 | 0.15 ± 0.006 | 0.15 ± 0.01 | 0.14 ± 0.002 | ND | ND | ND | ND |
| **Lys** | 4.7 ± 0.069 | 4.36 ± 0.029 | 4.46 ± 0.038 | 4.3 ± 0.032 | 4.32 ± 0.04 | 4.24 ± 0.066 | 7.67 ± 0.5 | 7.59 ± 0.74 | 7.37 ± 0.07 | 7.27 ± 0.73 |
| **His** | 0.73 ± 0.015 | 0.66 ± 0.01 | 0.74 ± 0.007 | 0.71 ± 0.007 | 0.72 ± 0.019 | 0.66 ± 0.043 | 2.1 ± 0.12 | 2.06 ± 0.19 | 2 ± 0.02 | 1.96 ± 0.2 |
| **Trp** | 0.77 ± 0.025 | 0.7 ± 0.017 | 0.65 ± 0.01 | 0.64 ± 0.013 | 0.64 ± 0.019 | 0.6 ± 0.034 | 0.87 ± 0.1 | 0.84 ± 0.06 | 0.83 ± 0.05 | 0.78 ± 0.08 |
| **Arg** | 2.68 ± 0.041 | 2.45 ± 0.031 | 2.35 ± 0.01 | 2.28 ± 0.025 | 2.32 ± 0.098 | 2.24 ± 0.036 | 3.37 ± 0.2 | 3.27 ± 0.3 | 3.1 ± 0.05 | 3.02 ± 0.29 |
| **NH_3_** | 2.66 ± 0.03 | 4.47 ± 0.07 | 5.73 ± 0.04 | 5.72 ± 0.06 | 5.63 ± 0.28 | 6.01 ± 0.1 | 16.12 ± 1.263 | 17.81 ± 1.99 | 18 ± 0.31 | 18.57 ± 2.2 |

^a^ Mean and standard deviation of 3 biological replicates.

ND, not detected
