# Supplementary material for: A Temporal -omic Study of Propionibacterium freudenreichii CIRM-BIA1T Adaptation Strategies in Conditions Mimicking Cheese Ripening in the Cold
Source: PLoS One. 2012 Jan 13;7(1):e29083. doi: 10.1371/journal.pone.0029083 (PMC3258244; doi:10.1371/journal.pone.0029083)
Supplement: Table S2 — P. freudenreichii CIRM-BIA1T strain 565 differentially expressed genes according to microarray experiments ( P <0.05, |fold-change|>1). (DOC) [file pone.0029083.s004.doc]

**Table S2**: *P*. *freudenreichii* CIRM-BIA1^T^ strain 565 differentially expressed genes according to microarray experiments (*P* < 0.05, |fold-change| > 1).

| **Protein function, locus tag and gene name** | | |  | **Protein description** |  |  | | **Microarray** | | | | | |
| --- | --- | --- | --- | --- | --- | --- | --- | --- | --- | --- | --- | --- | --- |
|  |  |  |  |  |  | **Category** |  | **Fold-change (log_2_)** | | | |  | ***P* value** |
|  |  |  |  |  |  |  |  | **40 h** | **3 days** | **6 days** | **9 days** |  |  |
| **Adaptation to atypical conditions** | | |  |  |  |  |  |  |  |  |  |  |  |
| PFREUD_01410 |  |  |  | Histidine kinase A-like precursor |  | A |  | 0.39 | 1.61 | 1.34 | 1.35 |  | 0.001 |
| PFREUD_02870 |  | *dps* |  | Starvation-inducible DNA-binding protein |  | A |  | 0.34 | 3.28 | 2.31 | 1.22 |  | 0.005 |
| PFREUD_06100 |  |  |  | Resuscitation-promoting factor RpfB |  | A |  | -0.15 | -1.98 | -2.31 | -2.36 |  | 0.000 |
| PFREUD_06710 |  | *pspC* |  | Possible stress-response transcriptional regulator |  | A |  | 0.48 | 3.19 | 2.55 | 2.17 |  | 0.006 |
| PFREUD_09800 |  | *cspA* |  | Cold shock-like protein |  | A |  | 0.50 | 2.16 | 1.32 | 0.98 |  | 0.028 |
| PFREUD_16140 |  | *PF47* |  | Protein mrp homolog |  | A |  | 0.30 | -1.88 | -2.03 | -2.49 |  | 0.000 |
| PFREUD_16500 |  | *cstA* |  | Carbon starvation protein |  | A |  | 2.32 | -1.45 | -1.40 | -0.64 |  | 0.000 |
| PFREUD_18020 |  |  |  | Type I antifreeze protein |  | A |  | -0.42 | -1.31 | -1.08 | -1.15 |  | 0.003 |
| PFREUD_18210 |  | *cspB* |  | Cold shock protein |  | A |  | 0.12 | 2.56 | 1.86 | 0.97 |  | 0.038 |
| **Transport, biding and metabolism of amino acids and related molecules** | | |  |  |  |  |  |  |  |  |  |  |  |
| PFREUD_00380 |  | *cycA2* |  | D-serine/D-alanine/glycine transporter |  | AA |  | 1.21 | 3.19 | 2.29 | 1.78 |  | 0.002 |
| PFREUD_03090 |  | *serA* |  | Phosphoglycerate dehydrogenase |  | AA |  | 0.16 | 0.97 | 0.95 | 1.11 |  | 0.006 |
| PFREUD_03280 |  |  |  | ABC-type transport system |  | AA |  | -0.49 | -1.17 | -0.84 | -0.76 |  | 0.006 |
| PFREUD_03290 |  | *metN* |  | Methionine import ATP-binding protein metN |  | AA |  | -0.25 | -1.12 | -0.99 | -0.90 |  | 0.006 |
| PFREUD_06420 |  | *opuCB* |  | ABC transporter of choline |  | AA |  | -0.12 | -1.33 | -1.13 | -1.21 |  | 0.000 |
| PFREUD_06430 |  | *opuCA* |  | ABC transporter of Glycine betaine, carnitine, choline |  | AA |  | -0.24 | -1.63 | -1.48 | -1.53 |  | 0.002 |
| PFREUD_06440 |  | *opuBB* |  | ABC transporter of choline |  | AA |  | -0.28 | -1.19 | -1.03 | -0.80 |  | 0.002 |
| PFREUD_07640 |  | *argK* |  | Kinase ArgK |  | AA |  | 0.80 | 2.10 | 2.27 | 1.97 |  | 0.018 |
| PFREUD_09510 |  |  |  | Amino acid permease |  | AA |  | 0.34 | -1.25 | -1.04 | -0.96 |  | 0.003 |
| PFREUD_10030 |  |  |  | Binding protein of oligopeptide ABC transporter |  | AA |  | 0.73 | -2.95 | -2.29 | -2.79 |  | 0.004 |
| PFREUD_10840 |  | *livF* |  | ABC protein of branched-chain amino acid ABC transporter |  | AA |  | -0.58 | -3.11 | -2.56 | -2.35 |  | 0.000 |
| PFREUD_10850 |  | *livG* |  | ABC protein of branched-chain amino acid ABC transporter |  | AA |  | -0.43 | -3.34 | -2.87 | -3.09 |  | 0.002 |
| PFREUD_10860 |  | *braE (livM, livE)* |  | IM protein of branched-chain amino acid ABC transporter |  | AA |  | -0.31 | -1.27 | -1.12 | -0.90 |  | 0.003 |
| PFREUD_10870 |  | *braD (livH)* |  | IM protein of branched-chain amino acid ABC transporter |  | AA |  | -0.23 | -1.37 | -1.29 | -0.87 |  | 0.002 |
| PFREUD_10880 |  | *braC* |  | BP protein of branched-chain amino acid ABC transporter |  | AA |  | 0.21 | -2.36 | -2.12 | -2.25 |  | 0.000 |
| PFREUD_12690 |  | *ydaO* |  | IM protein of branched-chain amino acids ABC transporter |  | AA |  | -0.19 | -1.84 | -1.76 | -1.12 |  | 0.000 |
| PFREUD_18720 |  |  |  | Amino acid permease |  | AA |  | 0.34 | -1.34 | -1.67 | -1.50 |  | 0.005 |
| PFREUD_22930 |  |  |  | Polar amino acid ABC transporter, binding protein |  | AA |  | -0.35 | -1.27 | -1.55 | -2.03 |  | 0.021 |
| PFREUD_23230 |  | *gadB* |  | Glutamate decarboxylase |  | AA |  | 0.11 | 0.63 | 0.82 | 1.24 |  | 0.007 |
| PFREUD_23240 |  |  |  | Glutamate/gamma-aminobutyrate anti-porter |  | AA |  | 0.73 | 1.52 | 1.62 | 2.61 |  | 0.037 |
| PFREUD_00370 |  | *ald* |  | Alanine dehydrogenase |  | AA |  | 4.30 | 7.33 | 6.78 | 6.65 |  | 0.000 |
| PFREUD_00830 |  |  |  | Peptidase E-like |  | AA |  | 0.03 | 1.16 | 0.96 | 0.29 |  | 0.001 |
| PFREUD_00860 |  | *argF* |  | Ornithine carbamoyltransferase |  | AA |  | -0.07 | 1.04 | 0.61 | 0.54 |  | 0.005 |
| PFREUD_01460 |  | *argG* |  | Argininosuccinate synthase |  | AA |  | -0.44 | 2.87 | 2.30 | 0.64 |  | 0.003 |
| PFREUD_01580 |  | *aroE* |  | Shikimate 5-dehydrogenase |  | AA |  | 0.03 | 2.19 | 1.49 | 0.96 |  | 0.000 |
| PFREUD_01750 |  | *argE/dapE1* |  | Acetylornithine deacetylase/Succinyl-diaminopimelate desuccinylase related deacylase |  | AA |  | 0.24 | 1.88 | 1.30 | 1.13 |  | 0.002 |
| PFREUD_02200 |  | *bkdA2* |  | 2-oxoisovalerate dehydrogenase β subunit/Pyruvate dehydrogenase E1 component β subunit |  | AA |  | -0.89 | -3.16 | -3.02 | -2.50 |  | 0.011 |
| PFREUD_02770 |  | *dcm* |  | DNA (cytosine-5-)-methyltransferase |  | AA |  | 0.12 | -1.20 | -1.78 | -1.79 |  | 0.000 |
| PFREUD_08510 |  | *proA* |  | Gamma-glutamyl phosphate reductase |  | AA |  | -0.02 | 1.31 | 1.06 | 0.82 |  | 0.022 |
| PFREUD_09460 |  | *tyrB* |  | Aspartate transaminase |  | AA |  | -0.90 | -2.37 | -2.25 | -2.29 |  | 0.000 |
| PFREUD_09540 |  | *gabT* |  | 4-aminobutyrate aminotransferase |  | AA |  | 0.02 | -1.53 | -1.18 | -1.38 |  | 0.001 |
| PFREUD_11330 |  | *sppA* |  | Putative signal peptide peptidase |  | AA |  | 0.95 | -1.65 | -1.81 | -1.63 |  | 0.000 |
| PFREUD_11340 |  | *metH* |  | Methionine synthase |  | AA |  | -0.37 | -1.63 | -1.56 | -1.41 |  | 0.000 |
| PFREUD_11360 |  | *pepC* |  | Aminopeptidase C |  | AA |  | -0.12 | -1.91 | -1.86 | -1.66 |  | 0.002 |
| PFREUD_11470 |  | *carB* |  | Carbamoyl-phosphate synthase large chain |  | AA |  | -0.37 | -1.96 | -1.84 | -2.01 |  | 0.003 |
| PFREUD_11850 |  | *gltD* |  | Glutamate synthase small subunit |  | AA |  | -1.16 | -1.81 | -1.68 | -1.55 |  | 0.000 |
| PFREUD_11860 |  | *gltB* |  | Glutamate synthase large subunit |  | AA |  | -0.42 | -1.99 | -1.43 | -0.75 |  | 0.001 |
| PFREUD_13280 |  | *leuC* |  | 3-isopropylmalate dehydratase large subunit |  | AA |  | -0.32 | 0.39 | 1.31 | 0.62 |  | 0.013 |
| PFREUD_13960 |  | *argD* |  | Acetylornithine and succinylornithine aminotransferase |  | AA |  | -0.26 | 1.49 | 1.09 | 0.55 |  | 0.019 |
| PFREUD_13970 |  | *argB* |  | Acetylglutamate kinase |  | AA |  | -0.09 | 3.03 | 2.23 | 1.04 |  | 0.004 |
| PFREUD_13980 |  | *ArgJ* |  | Arginine biosynthesis bifunctional protein ArgJ |  | AA |  | -0.23 | 3.41 | 2.75 | 1.24 |  | 0.001 |
| PFREUD_13990 |  | *argC* |  | N-acetyl-gamma-glutamyl-phosphate reductase |  | AA |  | 0.06 | 2.45 | 2.10 | 0.88 |  | 0.011 |
| PFREUD_14220 |  | *trpB2* |  | Tryptophan synthase beta subunit |  | AA |  | 0.15 | -1.13 | -0.86 | -0.87 |  | 0.003 |
| PFREUD_15830 |  | *trpD* |  | Anthranilate phosphoribosyltransferase |  | AA |  | 0.32 | -1.70 | -1.71 | -1.82 |  | 0.001 |
| PFREUD_16250 |  | *dapD* |  | 2,3,4,5-tetrahydropyridine-2,6-dicarboxylate N-succinyltransferase |  | AA |  | -0.54 | -1.56 | -1.67 | -2.10 |  | 0.010 |
| PFREUD_16270 |  |  |  | Aminotransferase |  | AA |  | 0.41 | -1.90 | -1.65 | -1.82 |  | 0.007 |
| PFREUD_16320 |  | *aspA1* |  | Aspartate ammonia-lyase |  | AA |  | 1.23 | 2.16 | 1.73 | 1.65 |  | 0.002 |
| PFREUD_16330 |  | *aspA2* |  | Aspartate ammonia-lyase |  | AA |  | 1.50 | 2.85 | 2.52 | 2.01 |  | 0.001 |
| PFREUD_18570 |  | *sdaA* |  | L-serine dehydratase |  | AA |  | 0.20 | 1.97 | 1.61 | 0.90 |  | 0.003 |
| PFREUD_18740 |  | *glyA* |  | Glycine hydroxymethyltransferase precursor |  | AA |  | -0.20 | -1.03 | -0.98 | -1.41 |  | 0.031 |
| PFREUD_18990 |  | *ilvD* |  | Dihydroxy-acid dehydratase |  | AA |  | -0.40 | -1.08 | -0.93 | -0.83 |  | 0.002 |
| PFREUD_19090 |  | *iolD* |  | Myo-inositol catabolism iolD protein |  | AA |  | -0.23 | -1.60 | -1.34 | -1.41 |  | 0.021 |
| PFREUD_20440 |  | *mao* |  | Flavin-containing amine oxidase |  | AA |  | 0.37 | 1.15 | 1.09 | 0.99 |  | 0.002 |
| PFREUD_21690 |  | *nadB2* |  | L-aspartate oxidase |  | AA |  | -0.41 | -0.36 | -0.77 | -1.02 |  | 0.005 |
| **Metabolism of coenzymes and prosthetic groups** | | |  |  |  |  |  |  |  |  |  |  |  |
| PFREUD_01600 |  | *dkgA* |  | 2,5-diketo-D-gluconate reductase A |  | C |  | -0.49 | -1.30 | -1.35 | -1.12 |  | 0.019 |
| PFREUD_04940 |  | *ApbA* |  | Putative 2-dehydropantoate 2-reductase |  | C |  | 0.03 | 1.29 | 1.20 | 0.98 |  | 0.016 |
| PFREUD_05300 |  | *idsA* |  | Heptaprenyl diphosphate synthase component II |  | C |  | -0.82 | -1.21 | -1.49 | -1.89 |  | 0.018 |
| PFREUD_06370 |  | *bluB/cobT2* |  | Phosphoribosyltransferase/nitroreductase |  | C |  | 0.02 | -0.78 | -1.05 | -1.62 |  | 0.029 |
| PFREUD_06840 |  | *folD* |  | Methylenetetrahydrofolate dehydrogenase |  | C |  | 0.02 | -1.01 | -0.93 | -1.11 |  | 0.001 |
| PFREUD_07680 |  | *cbiL* |  | CbiL Precorrin-2 C20-methyltransferase |  | C |  | -0.12 | -0.89 | -0.98 | -1.29 |  | 0.016 |
| PFREUD_09200 |  | *nadC* |  | Nicotinate-nucleotide pyrophosphorylase |  | C |  | -0.77 | -1.09 | -1.18 | -1.38 |  | 0.001 |
| PFREUD_09360 |  | *chlD* |  | Mg-chelatase subunit ChlD |  | C |  | -0.77 | -2.98 | -2.43 | -2.18 |  | 0.002 |
| PFREUD_10080 |  |  |  | D-3-phosphoglycerate dehydrogenase |  | C |  | -0.74 | -2.27 | -2.03 | -1.89 |  | 0.000 |
| PFREUD_10520 |  | *lplA* |  | Lipoate-protein ligase A |  | C |  | -0.68 | -1.78 | -1.76 | -1.78 |  | 0.006 |
| PFREUD_12200 |  | *cbiP/cobQ* |  | Cobyric acid synthase CbiP/CobQ |  | C |  | -0.53 | -1.05 | -1.21 | -1.15 |  | 0.004 |
| PFREUD_12230 |  | *cobU* |  | CobU Bifunctional cobalamin biosynthesis pyrophosphate enzyme |  | C |  | -0.56 | -1.42 | -1.45 | -1.56 |  | 0.001 |
| PFREUD_12810 |  | *panE* |  | 2-dehydropantoate 2-reductase |  | C |  | 0.55 | 2.31 | 1.67 | 0.73 |  | 0.019 |
| PFREUD_14480 |  | *coaD* |  | Phosphopantetheine adenylyltransferase |  | C |  | 0.26 | -0.69 | -0.71 | -1.09 |  | 0.028 |
| PFREUD_15040 |  | *pdxS* |  | Pyridoxal biosynthesis lyase pdxS |  | C |  | -0.40 | -2.10 | -2.16 | -2.11 |  | 0.000 |
| PFREUD_15310 |  | *coaE* |  | Dephospho-CoA kinase |  | C |  | -0.60 | -1.16 | -1.02 | -1.02 |  | 0.005 |
| PFREUD_16550 |  | *thiG* |  | Thiazole biosynthesis family protein |  | C |  | 0.05 | -1.65 | -1.38 | -0.55 |  | 0.014 |
| PFREUD_18930 |  | *hemY* |  | Protoporphyrinogen oxidase |  | C |  | -1.05 | -2.36 | -2.25 | -2.77 |  | 0.001 |
| PFREUD_19260 |  | *hemL2* |  | Glutamate-1-semialdehyde 2,1-aminomutase |  | C |  | -0.69 | -2.06 | -1.83 | -2.38 |  | 0.013 |
| PFREUD_20270 |  | *folK* |  | Putative hydroxymethyldihydropteridine pyrophosphokinase |  | C |  | -0.05 | 1.32 | 0.72 | 0.31 |  | 0.003 |
| PFREUD_21470 |  | *ribD* |  | Riboflavin-specific deaminase |  | C |  | 1.71 | 2.68 | 3.12 | 2.53 |  | 0.001 |
| PFREUD_21530 |  | *pntB* |  | NADH dehydrogenase |  | C |  | -0.51 | -2.54 | -2.19 | -1.79 |  | 0.001 |
| PFREUD_21540 |  | *pntA* |  | NAD(P)(+) transhydrogenase |  | C |  | -0.48 | -1.75 | -1.94 | -1.82 |  | 0.001 |
| PFREUD_22860 |  | *pdxA2* |  | 4-hydroxythreonine-4-phosphate dehydrogenase |  | C |  | 0.64 | 1.03 | 1.02 | 1.62 |  | 0.005 |
| PFREUD_23530 |  |  |  | Isochorismatase hydrolase |  | C |  | -0.14 | 1.03 | 0.81 | 0.65 |  | 0.001 |
| **Cell division** | | |  |  |  |  |  |  |  |  |  |  |  |
| PFREUD_09600 |  | *ftsX* |  | Cell division protein |  | CD |  | -0.85 | -4.21 | -3.75 | -2.78 |  | 0.000 |
| **Cell wall, cell envelope and cellular processes** | | |  |  |  |  |  |  |  |  |  |  |  |
| PFREUD_00390 |  |  |  | ErfK/YbiS/YcfS/YnhG precursor |  | CE |  | -0.41 | -1.03 | -1.14 | -0.83 |  | 0.019 |
| PFREUD_00630 |  | *gtfD* |  | Glycosyl transferase, family 2 |  | CE |  | 0.41 | 1.16 | 0.71 | 0.56 |  | 0.003 |
| PFREUD_01080 |  |  |  | Secreted transglycosydase |  | CE |  | -0.57 | -1.17 | -1.77 | -1.61 |  | 0.029 |
| PFREUD_02380 |  | *murQ* |  | N-acetylmuramic acid 6-phosphate |  | CE |  | 1.13 | -0.30 | -0.51 | -0.27 |  | 0.001 |
| PFREUD_02920 |  |  |  | Peptidoglycan binding domain protein |  | CE |  | 1.70 | 3.87 | 3.34 | 2.38 |  | 0.000 |
| PFREUD_10950 |  | *dpm* |  | Dolichyl-phosphate beta-D-mannosyltransferase |  | CE |  | 0.13 | -2.10 | -2.25 | -1.94 |  | 0.000 |
| PFREUD_12410 |  |  |  | Acyltransferase |  | CE |  | -1.21 | -3.48 | -2.86 | -2.85 |  | 0.003 |
| PFREUD_13250 |  | *ddlA* |  | D-alanine-D-alanine ligase |  | CE |  | -0.32 | -1.13 | -0.98 | -0.84 |  | 0.000 |
| PFREUD_15590 |  | *mraZ* |  | Protein mraZ |  | CE |  | -0.51 | 3.93 | 4.33 | 5.16 |  | 0.000 |
| PFREUD_19600 |  | *pbpA* |  | Penicillin-binding protein |  | CE |  | -0.40 | -1.08 | -0.77 | 0.08 |  | 0.001 |
| PFREUD_19740 |  | *srt* |  | Sortase family protein |  | CE |  | 0.30 | -1.27 | -1.11 | -0.49 |  | 0.001 |
| PFREUD_23030 |  | *slh2* |  | S-layer protein precursor |  | CE |  | 0.33 | 1.33 | 1.21 | 1.09 |  | 0.029 |
| PFREUD_23640 |  | *mrc* |  | Penicillin-binding protein |  | CE |  | -0.42 | -1.59 | -1.38 | -1.72 |  | 0.001 |
| PFREUD_24190 |  | *oxaA* |  | Conserved membrane protein |  | CE |  | 0.44 | -2.96 | -2.79 | -1.92 |  | 0.000 |
| **Carbohydrates and related molecules: transport, biding, metabolism, glycolytic pathway, TCA cycle** | | |  |  |  |  |  |  |  |  |  |  |  |
| PFREUD_02360 |  | *galP* |  | Sodium:galactoside symporter |  | CH |  | 1.28 | -0.76 | -0.73 | -0.33 |  | 0.001 |
| PFREUD_02650 |  |  |  | Sugar transporter of a major facilitator superfamily 2.A.1.1.15 |  | CH |  | -0.38 | -2.48 | -2.18 | -2.10 |  | 0.000 |
| PFREUD_06650 |  | *ytfT (rbsC)* |  | Ribose transport system permease protein RbsC, putative ABC transporter permease protein |  | CH |  | 0.38 | 0.75 | 1.10 | 1.01 |  | 0.032 |
| PFREUD_16340 |  | *dcuA* |  | C4-dicarboxylate transporter |  | CH |  | 1.27 | 2.34 | 1.73 | 2.48 |  | 0.010 |
| PFREUD_18660 |  | *lldP* |  | L-lactate permease |  | CH |  | 0.43 | -1.50 | -1.37 | -1.43 |  | 0.018 |
| PFREUD_20910 |  | *slgT* |  | Sodium/glucose co-transporter |  | CH |  | 0.35 | -1.99 | -1.84 | -1.36 |  | 0.001 |
| PFREUD_22990 |  | *iolT1* |  | Major myo-inositol transporter iolT1 |  | CH |  | 0.24 | 1.75 | 1.65 | 2.52 |  | 0.027 |
| PFREUD_00470 |  | *rmlA* |  | Glucose-1-phosphate thymidylyltransferase |  | CH |  | -0.11 | -1.10 | -1.05 | -0.67 |  | 0.036 |
| PFREUD_02370 |  | *lacZ* |  | Beta-galactosidase LacZ |  | CH |  | 1.59 | -1.46 | -1.23 | -1.04 |  | 0.011 |
| PFREUD_07170 |  | *pccB* |  | Propionyl-CoA carboxylase beta chain |  | CH |  | 0.26 | -1.50 | -1.36 | -1.41 |  | 0.001 |
| PFREUD_07400 |  | *pmm* |  | Phosphomannomutase |  | CH |  | -0.35 | -1.32 | -1.49 | -1.35 |  | 0.001 |
| PFREUD_10610 |  | *pgm1* |  | Phosphoglucomutase |  | CH |  | 2.09 | 3.39 | 3.43 | 3.37 |  | 0.000 |
| PFREUD_14600 |  | *glpK* |  | Glycerol kinase |  | CH |  | 1.76 | -0.44 | 0.13 | 0.20 |  | 0.010 |
| PFREUD_16180 |  | *glgC* |  | Glucose-1-phosphate adenylyltransferase |  | CH |  | 0.80 | 2.55 | 1.96 | 1.12 |  | 0.011 |
| PFREUD_16190 |  | *glgA* |  | Glycosyltransferase (glycogen synthase) |  | CH |  | 0.30 | 1.98 | 1.75 | 0.92 |  | 0.004 |
| PFREUD_18490 |  | *manA* |  | Phosphomannose isomerase |  | CH |  | 0.46 | 1.79 | 1.56 | 1.36 |  | 0.004 |
| PFREUD_19300 |  | *aldB* |  | Lactaldehyde dehydrogenase |  | CH |  | 1.21 | -1.06 | -0.85 | -0.34 |  | 0.000 |
| PFREUD_19880 |  |  |  | Inositol-1-phosphate synthase |  | CH |  | 0.95 | -1.28 | -1.32 | -1.72 |  | 0.004 |
| PFREUD_20040 |  |  |  | Secreted glycosyl hydrolase |  | CH |  | -0.02 | 1.40 | 1.00 | 0.46 |  | 0.034 |
| PFREUD_22380 |  | *araM* |  | L-arabinose utilization protein |  | CH |  | 0.15 | -1.08 | -1.16 | -1.32 |  | 0.000 |
| PFREUD_01040 |  | *gntK* |  | Gluconate kinase |  | CH |  | 1.52 | 2.29 | 1.70 | 1.56 |  | 0.001 |
| PFREUD_04290 |  | *pgi* |  | Glucose-6-phosphate isomerase |  | CH |  | 0.97 | 1.56 | 1.06 | 1.20 |  | 0.001 |
| PFREUD_08550 |  | *gmpB* |  | Phosphoglycerate mutase |  | CH |  | 0.36 | -1.04 | -1.00 | -1.09 |  | 0.002 |
| PFREUD_09470 |  | *aceE* |  | Pyruvate dehydrogenase E1 component |  | CH |  | 0.43 | -1.68 | -1.72 | -2.02 |  | 0.001 |
| PFREUD_12930 |  | *phk* |  | Phosphoketolase pyrophosphate |  | CH |  | 0.63 | 1.30 | 1.71 | 1.54 |  | 0.001 |
| PFREUD_15060 |  | *zwf* |  | Glucose-6-phosphate 1-dehydrogenase |  | CH |  | -0.79 | -1.30 | -1.47 | -1.26 |  | 0.000 |
| PFREUD_22360 |  | *tkt* |  | Transketolase |  | CH |  | 0.14 | 2.89 | 2.15 | 0.91 |  | 0.005 |
| PFREUD_22370 |  | *araB* |  | L-ribulokinase |  | CH |  | 0.76 | -0.31 | -0.98 | -1.02 |  | 0.002 |
| PFREUD_23890 |  | *fba2* |  | Fructose-bisphosphate aldolase class I |  | CH |  | 2.29 | 3.82 | 3.39 | 2.98 |  | 0.000 |
| PFREUD_06870 |  | *icd* |  | Putative isocitrate/isopropylmalate dehydrogenase |  | CH |  | 0.21 | -3.13 | -2.97 | -3.35 |  | 0.002 |
| PFREUD_12590 |  | *acn* |  | Aconitase, Aconitate hydratase |  | CH |  | 0.63 | -1.01 | -0.73 | -0.76 |  | 0.026 |
| PFREUD_23910 |  | *gltA1* |  | Citrate synthase |  | CH |  | 0.88 | 1.41 | 0.55 | 0.27 |  | 0.005 |
| PFREUD_19470 |  | *ptsI* |  | PTS enzyme I |  | CH |  | 0.90 | 3.45 | 2.71 | 2.28 |  | 0.000 |
| PFREUD_03230 |  | *ppdk* |  | Pyruvate phosphate dikinase |  | CH |  | 1.60 | 1.91 | 2.19 | 1.36 |  | 0.007 |
| PFREUD_06280 |  | *glmS* |  | Glucosamine-fructose-6-phosphate aminotransferase |  | CH |  | -0.46 | -0.92 | -1.17 | -1.19 |  | 0.032 |
| PFREUD_10890 |  | *lpd* |  | Dihydrolipoyl dehydrogenase |  | CH |  | 0.48 | -1.50 | -1.40 | -2.11 |  | 0.018 |
| PFREUD_16410 |  |  |  | Diacylglycerol kinase, catalytic region precursor |  | CH |  | 0.41 | 1.30 | 1.26 | 1.93 |  | 0.030 |
| PFREUD_11970 |  |  |  | IM protein of monosaccharide ABC transporter |  | CH |  | -0.69 | -4.45 | -4.02 | -4.04 |  | 0.000 |
| PFREUD_11980 |  |  |  | ABC protein of ABC transporter |  | CH |  | -0.28 | -1.60 | -1.50 | -1.30 |  | 0.000 |
| **DNA replication, restriction, modification, recombination and repair** | | |  |  |  |  |  |  |  |  |  |  |  |
| PFREUD_04260 |  |  |  | DeaD/DeaH box helicase |  | DNA |  | 0.06 | 1.01 | 0.86 | 1.25 |  | 0.019 |
| PFREUD_11830 |  | *dnaE1* |  | DNA polymerase III alpha subunit |  | DNA |  | -0.49 | -1.56 | -1.52 | -1.14 |  | 0.000 |
| PFREUD_13460 |  |  |  | Superfamily II RNA helicase |  | DNA |  | 0.01 | 1.93 | 1.97 | 1.30 |  | 0.000 |
| PFREUD_20810 |  | *dnaB* |  | Replicative DNA helicase |  | DNA |  | -0.70 | -2.82 | -2.83 | -2.06 |  | 0.000 |
| PFREUD_21140 |  |  |  | Helicase |  | DNA |  | -0.26 | -0.84 | -1.30 | -1.28 |  | 0.006 |
| PFREUD_01240 |  | *hsdS* |  | Type I restriction enzyme EcoR124II specificity protein |  | DNA |  | -0.45 | -1.28 | -1.39 | -1.07 |  | 0.005 |
| PFREUD_01250 |  |  |  | Methylase_S, type I restriction enzyme |  | DNA |  | -0.66 | -2.10 | -1.77 | -1.86 |  | 0.028 |
| PFREUD_06390 |  | *mutT* |  | 8-dihydro-8-oxoguanine-triphosphatase |  | DNA |  | -0.09 | 1.79 | 1.77 | 1.01 |  | 0.037 |
| PFREUD_15300 |  | *uvrB* |  | UvrABC system protein B |  | DNA |  | -0.44 | -1.31 | -0.98 | -1.24 |  | 0.010 |
| PFREUD_17480 |  | *tatD* |  | DNase |  | DNA |  | -0.82 | -1.90 | -1.88 | -1.87 |  | 0.000 |
| PFREUD_20130 |  |  |  | Endonuclease |  | DNA |  | -0.11 | 1.02 | 0.88 | 0.44 |  | 0.020 |
| PFREUD_02730 |  | *naeIV* |  | NaeI very short patch repair endonuclease |  | DNA |  | -0.59 | -1.24 | -1.22 | -1.05 |  | 0.007 |
| PFREUD_10560 |  |  |  | Nuclease of the RecB family |  | DNA |  | -0.14 | -1.02 | -0.92 | -1.28 |  | 0.004 |
| PFREUD_11720 |  | *ruvC* |  | Crossover junction endodeoxyribonuclease |  | DNA |  | -0.70 | -1.66 | -1.39 | -1.35 |  | 0.002 |
| PFREUD_14250 |  |  |  | Putative endonuclease |  | DNA |  | -0.31 | -0.46 | 0.66 | 1.23 |  | 0.032 |
| PFREUD_14440 |  | *mutM3* |  | Formamidopyrimidine-DNA glycosylase |  | DNA |  | -0.23 | -1.42 | -1.18 | -1.02 |  | 0.007 |
| PFREUD_23010 |  | *nfo* |  | Endonuclease IV |  | DNA |  | 0.01 | 3.98 | 3.53 | 2.51 |  | 0.001 |
| **Membrane bioenergetics: electron transport chain and ATP synthase** | | |  |  |  |  |  |  |  |  |  |  |  |
| PFREUD_00420 |  |  |  | Nitroreductase |  | E |  | 0.46 | 1.50 | 0.87 | 0.77 |  | 0.001 |
| PFREUD_01470 |  |  |  | NADPH:quinone reductase and related Zn-dependent oxidoreductases |  | E |  | 0.71 | 1.46 | 1.16 | 0.57 |  | 0.008 |
| PFREUD_01720 |  | *cydA* |  | Cytochrome d ubiquinol oxidase subunit I |  | E |  | 0.74 | -1.67 | -1.69 | -1.75 |  | 0.002 |
| PFREUD_01730 |  | *cydB* |  | Cytochrome d ubiquinol oxidase, subunit II |  | E |  | 0.68 | -1.10 | -1.12 | -1.30 |  | 0.002 |
| PFREUD_01830 |  |  |  | FAD-dependent pyridine nucleotide-disulphide oxidoreductase:4Fe-4S ferredoxin, iron-sulfur binding:Aromatic-ring hydroxylase |  | E |  | 0.82 | -1.63 | -1.19 | -0.37 |  | 0.021 |
| PFREUD_04670 |  |  |  | BadF/BadG/BcrA/BcrD ATPase family protein |  | E |  | 0.53 | 1.76 | 1.60 | 1.21 |  | 0.022 |
| PFREUD_05140 |  |  |  | Electron transfer oxidoreductase |  | E |  | 0.78 | -0.65 | -1.13 | -1.47 |  | 0.004 |
| PFREUD_05160 |  | *nuoA* |  | NADH-quinone oxidoreductase chain |  | E |  | 0.20 | -1.50 | -1.35 | -1.21 |  | 0.001 |
| PFREUD_05170 |  | *nuoB* |  | NADH-quinone oxidoreductase chain B |  | E |  | 0.34 | -2.00 | -1.92 | -1.81 |  | 0.000 |
| PFREUD_05180 |  | *nuoC* |  | NADH-quinone oxidoreductase chain C |  | E |  | 0.01 | -3.59 | -3.20 | -3.15 |  | 0.000 |
| PFREUD_05190 |  | *nuoD* |  | NADH-quinone oxidoreductase chain D |  | E |  | 0.30 | -3.96 | -3.29 | -3.83 |  | 0.002 |
| PFREUD_05200 |  | *nuoE* |  | NADH-quinone oxidoreductase chain E |  | E |  | 0.26 | -2.71 | -2.44 | -2.83 |  | 0.000 |
| PFREUD_05210 |  | *nuoF* |  | NADH-quinone oxidoreductase chain F |  | E |  | -0.09 | -2.37 | -2.03 | -2.27 |  | 0.000 |
| PFREUD_05220 |  | *nuoG* |  | NADH-quinone oxidoreductase chain G |  | E |  | -0.26 | -1.74 | -1.66 | -2.26 |  | 0.010 |
| PFREUD_05250 |  | *nuoJ* |  | NADH-quinone oxidoreductase chain J |  | E |  | -0.30 | -1.31 | -1.43 | -1.82 |  | 0.030 |
| PFREUD_05260 |  | *nuoK* |  | NADH dehydrogenase I chain K |  | E |  | -0.21 | -1.97 | -2.25 | -2.10 |  | 0.002 |
| PFREUD_05270 |  | *nuoL* |  | NADH dehydrogenase chain L |  | E |  | -0.70 | -1.80 | -2.00 | -2.94 |  | 0.023 |
| PFREUD_05280 |  | *nuoM* |  | NADH dehydrogenase I chain M |  | E |  | -0.66 | -1.46 | -1.45 | -2.44 |  | 0.015 |
| PFREUD_06700 |  |  |  | NADPH:quinone reductase related Zn-dependent oxidoreductase |  | E |  | 0.78 | 2.85 | 2.39 | 1.66 |  | 0.003 |
| PFREUD_09240 |  | *sdhC1* |  | Succinate dehydrogenase subunit C |  | E |  | -0.09 | -2.07 | -2.08 | -2.18 |  | 0.001 |
| PFREUD_09250 |  | *sdhA* |  | Succinate dehydrogenase, subunit A |  | E |  | -0.21 | -2.78 | -2.44 | -3.24 |  | 0.012 |
| PFREUD_10430 |  | *atpB* |  | ATP synthase A chain |  | E |  | 0.16 | -2.87 | -2.54 | -2.80 |  | 0.001 |
| PFREUD_10440 |  | *atpE* |  | ATP synthase C chain |  | E |  | -0.33 | -2.38 | -2.21 | -2.22 |  | 0.000 |
| PFREUD_10450 |  | *atpF* |  | ATP synthase B chain |  | E |  | -0.24 | -2.00 | -2.08 | -2.36 |  | 0.000 |
| PFREUD_10460 |  | *atpH* |  | ATP synthase delta chain |  | E |  | -0.48 | -3.05 | -2.73 | -2.55 |  | 0.000 |
| PFREUD_10470 |  | *atpA* |  | ATP synthase subunit alpha |  | E |  | -0.77 | -3.61 | -3.36 | -3.87 |  | 0.000 |
| PFREUD_10480 |  | *atpG* |  | ATP synthase gamma chain |  | E |  | -0.39 | -3.63 | -3.28 | -3.66 |  | 0.002 |
| PFREUD_10490 |  | *atpD* |  | ATP synthase subunit beta |  | E |  | -0.74 | -2.44 | -2.33 | -2.39 |  | 0.000 |
| PFREUD_10500 |  | *atpC* |  | ATP synthase epsilon chain |  | E |  | -0.81 | -2.64 | -2.72 | -2.84 |  | 0.005 |
| PFREUD_14310 |  | *sdhA3* |  | Succinate dehydrogenase flavoprotein subunit |  | E |  | 0.52 | -2.77 | -2.89 | -2.94 |  | 0.011 |
| PFREUD_14320 |  | *sdhC2* |  | Succinate dehydrogenase cytochrome B-558 subunit |  | E |  | 0.77 | -2.07 | -2.08 | -1.36 |  | 0.002 |
| PFREUD_14960 |  |  |  | Dioxygenase |  | E |  | -1.16 | 0.38 | -0.02 | -2.29 |  | 0.031 |
| PFREUD_16650 |  |  |  | FAD dependent oxidoreductase |  | E |  | 0.51 | 2.38 | 2.40 | 3.21 |  | 0.001 |
| PFREUD_16930 |  | *ychF* |  | GTPase YchF |  | E |  | -0.44 | -1.61 | -1.38 | -1.24 |  | 0.000 |
| **Lipid metabolism** | | |  |  |  |  |  |  |  |  |  |  |  |
| PFREUD_08060 |  |  |  | Long-chain fatty-acid CoA ligase |  | L |  | 0.12 | -1.48 | -1.10 | -1.82 |  | 0.041 |
| PFREUD_08940 |  | *uppS2* |  | Undecaprenyl diphosphate synthase |  | L |  | 0.23 | -2.17 | -1.89 | -1.52 |  | 0.000 |
| PFREUD_09410 |  | *fabF* |  | Beta-ketoacyl-ACP synthase |  | L |  | -1.94 | -3.53 | -3.58 | -3.81 |  | 0.000 |
| PFREUD_09420 |  | *acpP* |  | Acyl carrier protein |  | L |  | -2.01 | -3.79 | -3.80 | -3.85 |  | 0.000 |
| PFREUD_09430 |  | *fabH* |  | 3-oxoacyl-(Acyl-carrier-protein) synthase III |  | L |  | -2.74 | -4.53 | -4.35 | -4.51 |  | 0.000 |
| PFREUD_09440 |  | *fabD* |  | Malonyl CoA-acyl carrier protein transacylase |  | L |  | -2.28 | -3.08 | -3.12 | -2.93 |  | 0.000 |
| PFREUD_12420 |  | *pgs2* |  | CDP-diacylglycerol--glycerol-3-phosphate 3-phosphatidyl-transferase |  | L |  | -0.89 | -1.25 | -0.84 | -0.96 |  | 0.005 |
| PFREUD_16490 |  | *glxK/garK* |  | Glycerate kinase GlxK/GarK |  | L |  | 0.73 | 2.05 | 1.60 | 1.82 |  | 0.008 |
| PFREUD_17010 |  | *uppS* |  | Undecaprenyl pyrophosphate synthase |  | L |  | 0.32 | 2.12 | 1.84 | 1.14 |  | 0.013 |
| PFREUD_23000 |  | *PPA2234* |  | Putative long-chain-fatty-acid--CoA ligase/synthetase |  | L |  | -0.28 | 4.36 | 3.84 | 3.85 |  | 0.000 |
| **Miscellaneous** | | |  |  |  |  |  |  |  |  |  |  |  |
| PFREUD_01550 |  | *vanZ* |  | VanZ |  | Mi |  | 0.58 | 1.73 | 1.62 | 1.84 |  | 0.047 |
| PFREUD_03760 |  |  |  | Hemerythrin HHE cation binding region |  | Mi |  | -1.03 | -0.57 | -0.76 | -0.70 |  | 0.048 |
| PFREUD_04310 |  |  |  | Beta-lactamase |  | Mi |  | 0.34 | 1.21 | 0.99 | 0.97 |  | 0.001 |
| PFREUD_05410 |  | *ahpC* |  | Peroxiredoxin/Alkyl hydroperoxide reductase subunit C |  | Mi |  | 0.14 | -0.42 | -1.17 | -2.05 |  | 0.050 |
| PFREUD_05420 |  | *ahpF* |  | Alkyl hydroperoxide reductase subunit F |  | Mi |  | -0.01 | -1.69 | -1.65 | -2.01 |  | 0.007 |
| PFREUD_12650 |  | *yghU* |  | Glutathione S-transferase |  | Mi |  | 0.25 | -1.86 | -1.92 | -1.83 |  | 0.000 |
| PFREUD_12660 |  | *yghU* |  | Glutathione S-transferase |  | Mi |  | 0.09 | -1.13 | -1.03 | -1.09 |  | 0.016 |
| PFREUD_15280 |  |  |  | Beta-lactamase-like |  | Mi |  | -1.00 | -1.26 | -0.99 | -1.18 |  | 0.008 |
| PFREUD_15290 |  | *terC* |  | Membrane protein TerC |  | Mi |  | 1.02 | -0.74 | -0.89 | -0.64 |  | 0.018 |
| PFREUD_01210 |  |  |  | ADP-ribose-1-monophosphate processing activity |  | Mi |  | 0.75 | 1.50 | 1.06 | 0.71 |  | 0.002 |
| PFREUD_01440 |  | *npdA* |  | Silent information regulator protein Sir2 /NAD-dependent deacetylase |  | Mi |  | -0.03 | 1.41 | 0.66 | 0.06 |  | 0.023 |
| PFREUD_02150 |  |  |  | Methyltransferase |  | Mi |  | -0.47 | 2.25 | 2.02 | 0.92 |  | 0.001 |
| PFREUD_02260 |  |  |  | Aldo/keto reductase |  | Mi |  | 0.19 | 1.03 | 0.72 | 0.38 |  | 0.007 |
| PFREUD_04730 |  |  |  | Kinase |  | Mi |  | -0.67 | -1.32 | -1.18 | -1.14 |  | 0.003 |
| PFREUD_06190 |  |  |  | Methyltransferase |  | Mi |  | -1.27 | -1.92 | -1.94 | -2.30 |  | 0.000 |
| PFREUD_07870 |  |  |  | Metal dependent phosphohydrolase |  | Mi |  | 0.45 | 1.22 | 0.98 | 0.95 |  | 0.005 |
| PFREUD_08320 |  |  |  | Thiredoxine like membrane protein |  | Mi |  | 0.19 | -2.20 | -2.19 | -1.40 |  | 0.000 |
| PFREUD_09370 |  |  |  | Von Willebrand factor, type A |  | Mi |  | -0.25 | -1.32 | -1.22 | -1.05 |  | 0.001 |
| PFREUD_09910 |  |  |  | Metal dependant hydrolase |  | Mi |  | 0.03 | -0.86 | -0.81 | -1.02 |  | 0.001 |
| PFREUD_11320 |  |  |  | HAD-superfamily hydrolase |  | Mi |  | -0.50 | -1.04 | -0.99 | -0.80 |  | 0.013 |
| PFREUD_12430 |  |  |  | HIT family protein |  | Mi |  | -0.72 | -1.87 | -1.66 | -1.32 |  | 0.000 |
| PFREUD_13110 |  |  |  | Metal dependent phosphohydrolase |  | Mi |  | 0.85 | 1.26 | 1.10 | 0.78 |  | 0.041 |
| PFREUD_13320 |  |  |  | Membrane protease |  | Mi |  | 0.34 | 0.81 | 1.02 | 0.86 |  | 0.004 |
| PFREUD_15190 |  |  |  | ATPase |  | Mi |  | -0.01 | 1.02 | 1.00 | 0.43 |  | 0.049 |
| PFREUD_15710 |  |  |  | Zn-dependent hydrolase, beta-lactamase fold |  | Mi |  | 0.31 | 1.78 | 1.66 | 1.20 |  | 0.005 |
| PFREUD_17460 |  | *ispE* |  | 4-diphosphocytidyl-2-C-methyl-D-erythritol kinase |  | Mi |  | 0.04 | 1.23 | 1.21 | 0.78 |  | 0.003 |
| PFREUD_18940 |  |  |  | Fe-S oxidoreductase |  | Mi |  | -1.20 | -1.94 | -1.83 | -1.95 |  | 0.000 |
| PFREUD_19920 |  |  |  | GCN5-related N-acetyltransferase |  | Mi |  | 0.07 | 1.51 | 1.13 | 0.49 |  | 0.008 |
| PFREUD_20050 |  |  |  | Pyridine nucleotide-disulphide oxidoreductase |  | Mi |  | 0.23 | 2.73 | 2.24 | 1.23 |  | 0.013 |
| PFREUD_20260 |  |  |  | Lsr2-like protein |  | Mi |  | -0.17 | 1.15 | 0.03 | -0.18 |  | 0.002 |
| PFREUD_22510 |  |  |  | Amidohydrolase 2 |  | Mi |  | 0.18 | 0.40 | 0.63 | 1.12 |  | 0.006 |
| PFREUD_23280 |  |  |  | Dehydrogenase/reductase |  | Mi |  | 0.35 | 1.06 | 0.84 | 0.78 |  | 0.019 |
| **Nucleotides and nucleic acids: metabolism, transport, biding** | | |  |  |  |  |  |  |  |  |  |  |  |
| PFREUD_04770 |  | *purF* |  | Amidophosphoribosyltransferase |  | Nt |  | -0.50 | -1.00 | -1.09 | -1.07 |  | 0.044 |
| PFREUD_05980 |  | *adk* |  | Adenylate kinase |  | Nt |  | -0.62 | -3.63 | -3.18 | -3.17 |  | 0.000 |
| PFREUD_11480 |  | *carA* |  | Carbamoyl-phosphate synthase small chain |  | Nt |  | 0.11 | -1.89 | -1.89 | -1.89 |  | 0.001 |
| PFREUD_21820 |  | *hit* |  | Histidine triad (HIT) protein |  | Nt |  | 0.26 | 1.37 | 0.90 | 0.35 |  | 0.002 |
| PFREUD_00050 |  | *sqdB* |  | UDP-sulfoquinovose synthase |  | Nt |  | -0.12 | -0.79 | -1.22 | -0.92 |  | 0.004 |
| PFREUD_04780 |  | *purM* |  | Phosphoribosylformylglycinamidine cyclo-ligase |  | Nt |  | -0.65 | -1.01 | -1.26 | -1.45 |  | 0.013 |
| PFREUD_06480 |  | *guaB1* |  | Inosine-5 -monophosphate dehydrogenase |  | Nt |  | 0.10 | -1.14 | -1.32 | -1.27 |  | 0.002 |
| PFREUD_06680 |  | *guaA* |  | GMP synthase |  | Nt |  | -0.44 | -1.04 | -1.07 | -1.52 |  | 0.011 |
| PFREUD_06820 |  | *purN* |  | 5 -phosphoribosylglycinamide formyltransferase |  | Nt |  | -0.42 | -1.01 | -1.24 | -1.00 |  | 0.002 |
| PFREUD_06830 |  | *purH* |  | Phosphoribosylaminoimidazolecarboxamide formyltransferase |  | Nt |  | -0.46 | -2.55 | -2.30 | -2.46 |  | 0.000 |
| PFREUD_07600 |  |  |  | NTPase/HAM1 |  | Nt |  | -0.31 | -1.12 | -0.89 | -1.17 |  | 0.028 |
| PFREUD_07610 |  | *thyA* |  | Thymidylate synthase |  | Nt |  | -1.08 | -1.92 | -1.50 | -1.50 |  | 0.030 |
| PFREUD_08080 |  | *thiD2* |  | Hydroxymethylpyrimidine/phosphomethylpyrimidine kinase |  | Nt |  | -0.13 | -1.64 | -1.14 | -1.23 |  | 0.002 |
| PFREUD_08360 |  | *ndk* |  | Nucleoside-diphosphate kinase |  | Nt |  | -0.16 | -1.06 | -1.12 | -1.23 |  | 0.002 |
| PFREUD_11430 |  | *gmk* |  | GMP kinase |  | Nt |  | 2.52 | -4.18 | -3.90 | -3.36 |  | 0.000 |
| PFREUD_12550 |  | *rnd* |  | Ribonuclease D |  | Nt |  | -0.12 | -1.38 | -1.34 | -1.56 |  | 0.003 |
| PFREUD_13640 |  | *nudF* |  | ADP-ribose pyrophosphatase |  | Nt |  | -0.69 | -1.58 | -1.41 | -1.58 |  | 0.006 |
| PFREUD_13650 |  | *pyrG* |  | CTP synthase |  | Nt |  | -1.22 | -3.71 | -3.12 | -3.50 |  | 0.004 |
| PFREUD_17400 |  | *prs* |  | Ribose-phosphate pyrophosphokinase |  | Nt |  | -0.44 | -1.56 | -1.49 | -1.92 |  | 0.032 |
| PFREUD_18700 |  |  |  | Amino acid permease |  | Nt |  | 0.48 | -1.07 | -1.25 | -1.30 |  | 0.017 |
| PFREUD_19200 |  | *pyrE* |  | Orotate phosphoribosyltransferase |  | Nt |  | -0.19 | -0.85 | -0.82 | -1.07 |  | 0.024 |
| PFREUD_19710 |  | *mesJ* |  | CMP/dCMP deaminase, zinc-binding |  | Nt |  | 0.12 | 1.17 | 1.29 | 1.03 |  | 0.005 |
| PFREUD_15030 |  | *pdxT* |  | Glutamine amidotransferase subunit pdxT |  | Nt |  | -0.20 | -1.62 | -1.80 | -2.05 |  | 0.000 |
| **Traduction process: ribosomal proteins, Aminoacyl tRNA synthetases, translation initiation and elongation proteins** | | |  |  |  |  |  |  |  |  |  |  |  |
| PFREUD_05530 |  | *rplK* |  | 50S ribosomal protein L11 |  | P |  | -0.67 | -1.88 | -1.14 | -1.31 |  | 0.004 |
| PFREUD_05620 |  | *rpsL* |  | 30S ribosomal protein S12 |  | P |  | -0.61 | -1.95 | -1.86 | -1.88 |  | 0.004 |
| PFREUD_05630 |  | *rpsG* |  | 30S ribosomal protein S7 |  | P |  | -0.46 | -2.19 | -1.80 | -1.48 |  | 0.003 |
| PFREUD_05760 |  | *rplV* |  | 50S ribosomal protein L22 |  | P |  | -1.59 | -1.79 | -1.60 | -1.92 |  | 0.032 |
| PFREUD_05780 |  | *rplP* |  | 50S ribosomal protein L16 |  | P |  | -1.48 | -1.44 | -1.29 | -1.43 |  | 0.012 |
| PFREUD_05820 |  | *rplX* |  | 50S ribosomal protein L24 |  | P |  | -1.14 | -1.57 | -1.47 | -1.54 |  | 0.022 |
| PFREUD_05840 |  | *rpsN1, rpsZ* |  | 30S ribosomal protein S14 type Z |  | P |  | -1.40 | -2.25 | -1.95 | -2.11 |  | 0.028 |
| PFREUD_05850 |  | *rpsH* |  | 30S ribosomal protein S8 |  | P |  | -1.23 | -1.51 | -1.53 | -1.54 |  | 0.029 |
| PFREUD_06040 |  | *rpsM* |  | 30S ribosomal protein S13 |  | P |  | -0.54 | -2.43 | -1.96 | -2.12 |  | 0.047 |
| PFREUD_06050 |  | *rpsK* |  | 30S ribosomal protein S11 |  | P |  | -0.68 | -2.92 | -2.12 | -1.92 |  | 0.004 |
| PFREUD_06060 |  | *rpsD* |  | 30S ribosomal protein S4 |  | P |  | -0.82 | -3.14 | -2.65 | -2.50 |  | 0.007 |
| PFREUD_06350 |  | *rimI* |  | Ribosomal-protein-alanine acetyltransferase |  | P |  | -0.87 | -1.68 | -1.68 | -1.55 |  | 0.000 |
| PFREUD_08680 |  | *rpsT* |  | 30S Ribosomal protein S20p |  | P |  | 0.47 | -0.90 | -0.90 | -1.03 |  | 0.002 |
| PFREUD_14350 |  | *rimM* |  | 16S rRNA processing protein |  | P |  | -0.49 | -1.88 | -1.21 | -1.67 |  | 0.033 |
| PFREUD_14370 |  | *rpsP* |  | 30S ribosomal protein S16 |  | P |  | -0.29 | -1.78 | -1.86 | -2.06 |  | 0.014 |
| PFREUD_14780 |  | *rpsB* |  | 30S ribosomal protein S2 |  | P |  | -0.80 | -1.86 | -1.54 | -2.59 |  | 0.049 |
| PFREUD_15330 |  | *rpsA* |  | 30S ribosomal protein S1 |  | P |  | -0.37 | -1.26 | -1.61 | -2.12 |  | 0.045 |
| PFREUD_00990 |  | *aatA* |  | Leucyl/phenylalanyl-tRNA-protein transferase |  | P |  | 0.54 | 1.48 | 0.86 | 0.85 |  | 0.003 |
| PFREUD_01060 |  | *aatB* |  | Leucyl/phenylalanyl-tRNA-protein transferase |  | P |  | 0.32 | 1.42 | 1.09 | 1.30 |  | 0.006 |
| PFREUD_06960 |  | *metG* |  | Methionyl-tRNA synthetase |  | P |  | -0.09 | -1.41 | -1.21 | -1.18 |  | 0.011 |
| PFREUD_08310 |  | *valS* |  | Valyl-tRNA synthetase |  | P |  | -0.22 | -1.44 | -1.23 | -1.68 |  | 0.027 |
| PFREUD_09190 |  | *glyQS* |  | Glycyl-tRNA synthetase |  | P |  | 0.27 | -1.06 | -0.81 | -0.26 |  | 0.014 |
| PFREUD_11310 |  |  |  | tRNA (adenine-N1-)-methyltransferase |  | P |  | -0.68 | -1.79 | -1.58 | -1.63 |  | 0.000 |
| PFREUD_11560 |  | *alaS* |  | Alanyl-tRNA synthetase (Alanine--tRNA ligase) |  | P |  | -0.61 | -1.26 | -1.04 | -1.14 |  | 0.013 |
| PFREUD_11590 |  | *aspS* |  | Aspartyl-tRNA synthetase |  | P |  | -0.16 | -1.03 | -0.91 | -1.08 |  | 0.024 |
| PFREUD_11940 |  | *glnS* |  | Glutaminyl-tRNA synthetase |  | P |  | 0.08 | -1.02 | -0.82 | -0.69 |  | 0.002 |
| PFREUD_12440 |  | *thrS* |  | Threonyl-tRNA synthetase |  | P |  | -0.39 | -1.30 | -1.05 | -1.01 |  | 0.003 |
| PFREUD_12680 |  |  |  | tRNA (uracil-5-)-methyltransferase |  | P |  | -0.86 | -2.20 | -1.84 | -1.89 |  | 0.002 |
| PFREUD_14690 |  | *proS* |  | Prolyl-tRNA synthetase |  | P |  | -0.46 | -1.56 | -1.35 | -1.35 |  | 0.000 |
| PFREUD_09680 |  |  |  | Ribosomal S30AE, sigma 54 modulation protein |  | P |  | 1.20 | 2.64 | 1.75 | 0.88 |  | 0.015 |
| PFREUD_05640 |  | *fusA* |  | Elongation factor G (EF-G) |  | P |  | -1.05 | -4.18 | -3.40 | -2.70 |  | 0.000 |
| PFREUD_05650 |  | *tuf* |  | Elongation factor Tu |  | P |  | -0.14 | -2.31 | -2.36 | -2.28 |  | 0.001 |
| PFREUD_06230 |  |  |  | GTP phosphohydrolase |  | P |  | 1.09 | 1.48 | 1.19 | 1.10 |  | 0.003 |
| PFREUD_13200 |  | *miaB2* |  | 2-methylthioadenine synthetase MiaB protein |  | P |  | 0.30 | 1.03 | 1.22 | 1.07 |  | 0.045 |
| PFREUD_10360 |  | *prfA* |  | Peptide chain release factor 1 |  | P |  | 0.63 | -1.87 | -1.58 | -1.80 |  | 0.001 |
| PFREUD_10750 |  | *gatC* |  | Glutamyl-tRNA(Gln) amidotransferase subunit C |  | P |  | 0.03 | -1.22 | -1.07 | -1.28 |  | 0.003 |
| PFREUD_10760 |  | *gatA* |  | Glutamyl-tRNA(Gln) amidotransferase subunit A |  | P |  | -0.25 | -1.09 | -1.08 | -1.43 |  | 0.003 |
| PFREUD_10770 |  | *gatB* |  | Aspartyl/glutamyl-tRNA(Asn/Gln) amidotransferase subunit B |  | P |  | -0.11 | -1.70 | -1.83 | -1.84 |  | 0.011 |
| **Protein degradation** | | |  |  |  |  |  |  |  |  |  |  |  |
| PFREUD_00340 |  |  |  | Amidohydrolase |  | PD |  | -0.60 | -1.01 | -1.15 | -0.95 |  | 0.020 |
| PFREUD_05990 |  | *map* |  | Methionine aminopeptidase |  | PD |  | -0.36 | -2.79 | -2.81 | -2.59 |  | 0.000 |
| PFREUD_06380 |  | *pepC* |  | Aminopeptidase C |  | PD |  | 0.26 | 1.73 | 2.01 | 1.85 |  | 0.001 |
| PFREUD_09940 |  |  |  | Secreted protease with a PDZ domain |  | PD |  | 0.80 | -1.47 | -1.61 | -1.40 |  | 0.000 |
| PFREUD_09990 |  | *pip1* |  | Proline iminopeptidase |  | PD |  | 1.09 | 3.07 | 2.71 | 1.76 |  | 0.003 |
| PFREUD_14810 |  | *def* |  | Polypeptide deformylase |  | PD |  | 0.27 | -2.39 | -2.11 | -1.94 |  | 0.000 |
| PFREUD_16430 |  |  |  | Membrane protease subunits |  | PD |  | -0.69 | 1.20 | 0.76 | 0.87 |  | 0.002 |
| **Phosphate metabolism** | | |  |  |  |  |  |  |  |  |  |  |  |
| PFREUD_08840 |  | *phoH* |  | Phosphate starvation-inducible protein PhoH-like protein |  | Ph |  | -0.14 | 1.90 | 2.11 | 1.62 |  | 0.024 |
| PFREUD_09830 |  |  |  | NUDIX hydrolase |  | Ph |  | 0.46 | 0.95 | 1.04 | 0.46 |  | 0.043 |
| PFREUD_23500 |  | *ppa* |  | Inorganic pyrophosphatase |  | Ph |  | 0.56 | 3.12 | 1.75 | 1.26 |  | 0.003 |
| **Protein modification and folding** | | |  |  |  |  |  |  |  |  |  |  |  |
| PFREUD_01070 |  | *pkaA* |  | Serine/threonine protein kinase |  | PM |  | -0.50 | -0.86 | -0.77 | -1.02 |  | 0.012 |
| PFREUD_01430 |  | *tpx* |  | Thiol peroxidase |  | PM |  | -0.28 | -0.99 | -1.59 | -1.49 |  | 0.006 |
| PFREUD_08330 |  | *dsbG* |  | DSBA oxidoreductase |  | PM |  | 0.05 | -2.40 | -2.19 | -1.40 |  | 0.000 |
| PFREUD_09630 |  | *smpB* |  | SmpB SsrA-binding protein |  | PM |  | -0.42 | -2.10 | -1.61 | -1.91 |  | 0.028 |
| PFREUD_10370 |  | *hemK* |  | Methylase |  | PM |  | -0.17 | -1.92 | -1.60 | -1.51 |  | 0.000 |
| PFREUD_10740 |  |  |  | Amino acid-binding ACT |  | PM |  | 0.44 | 1.15 | 1.27 | 0.76 |  | 0.039 |
| PFREUD_11140 |  |  |  | AAA ATPase, central region |  | PM |  | 0.33 | 3.08 | 2.69 | 1.89 |  | 0.008 |
| PFREUD_11290 |  | *prcA* |  | 20S proteasome alpha-subunit |  | PM |  | -0.41 | -1.82 | -1.63 | -1.52 |  | 0.003 |
| PFREUD_11300 |  | *pcrB* |  | 20S proteasome beta-subunit |  | PM |  | -0.16 | -1.33 | -1.11 | -0.77 |  | 0.003 |
| PFREUD_14280 |  | *lepB* |  | Signal peptidase I |  | PM |  | -0.26 | -1.33 | -1.38 | -1.33 |  | 0.040 |
| PFREUD_15410 |  | *LspA* |  | Lipoprotein signal peptidase, signal peptidase II |  | PM |  | 0.18 | -1.58 | -1.31 | -1.13 |  | 0.007 |
| PFREUD_19550 |  | *trxA6* |  | Thioredoxin |  | PM |  | -0.34 | -1.04 | -0.81 | -0.54 |  | 0.041 |
| PFREUD_24100 |  | *trxB* |  | Thioredoxin reductase |  | PM |  | -0.06 | -1.10 | -1.28 | -1.74 |  | 0.004 |
| PFREUD_24110 |  | *trxA1* |  | Thioredoxin |  | PM |  | 0.29 | -1.22 | -1.74 | -1.97 |  | 0.001 |
| PFREUD_04630 |  | *dnaK2* |  | Chaperone protein dnaK 2 |  | PM |  | -0.09 | -2.39 | -2.49 | -2.98 |  | 0.004 |
| PFREUD_04640 |  | *grpE2* |  | Protein GrpE 2 |  | PM |  | -0.05 | -2.42 | -2.34 | -2.59 |  | 0.000 |
| PFREUD_04650 |  | *dnaJ2* |  | Chaperone protein dnaJ 2 |  | PM |  | -0.46 | -1.00 | -1.37 | -1.71 |  | 0.029 |
| PFREUD_06460 |  | *groS1* | | 10 kDa chaperonin 1 |  | PM |  | -1,23 | -4.98 | -4.93 | -5.15 |  | 0.000 |
| PFREUD_06470 |  | *groEL1* |  | 60 kDa chaperonin 1 |  | PM |  | -0.77 | -3.38 | -3.73 | -3.93 |  | 0.001 |
| PFREUD_08210 |  | *tig* |  | Trigger factor (TF) |  | PM |  | -0.96 | -4.01 | -3.74 | -4.24 |  | 0.003 |
| PFREUD_09500 |  | *hsp20 2* |  | Heat shock protein 20 2 |  | PM |  | 0.74 | 0.02 | -0.42 | -1.33 |  | 0.038 |
| PFREUD_17820 |  | *dnaJ1* |  | Chaperone protein dnaJ 1 |  | PM |  | -0.30 | -2.43 | -2.17 | -2.01 |  | 0.001 |
| PFREUD_17830 |  | *grpE1* |  | Protein GrpE 1 |  | PM |  | 0.10 | -3.02 | -2.42 | -2.24 |  | 0.000 |
| PFREUD_17840 |  | *dnaK1* |  | Chaperone protein dnaK 1 |  | PM |  | 0.93 | -2.00 | -1.79 | -2.13 |  | 0.006 |
| PFREUD_17920 |  | *clpB 2* |  | Chaperone clpB 2 |  | PM |  | -0.46 | -2.86 | -2.82 | -2.68 |  | 0.000 |
| PFREUD_18470 |  | *groL2* | | 60 kDa chaperonin 2 |  | PM |  | -1,10 | -3.18 | -3.54 | -3.89 |  | 0.000 |
| PFREUD_22780 |  | *hsp20 1* |  | Heat shock protein 20 1 |  | PM |  | 0.77 | 2.85 | 2.71 | 2.50 |  | 0.001 |
| **Protein secretion** | | |  |  |  |  |  |  |  |  |  |  |  |
| PFREUD_05510 |  | *secE* |  | SecE/Sec61-gamma subunit of protein translocation complex |  | S |  | -0.49 | -1.33 | -1.54 | -1.45 |  | 0.038 |
| PFREUD_05970 |  | *secY* |  | Preprotein translocase SecY subunit |  | S |  | 0.13 | -1.56 | -1.54 | -1.29 |  | 0.002 |
| PFREUD_11670 |  | *secF* |  | Protein-export membrane protein secF |  | S |  | -0.30 | -1.92 | -1.59 | -2.06 |  | 0.037 |
| PFREUD_11680 |  | *secD* |  | Protein-export membrane protein secD |  | S |  | -0.11 | -1.67 | -1.41 | -1.77 |  | 0.006 |
| **Sensors: signal transduction** | | |  |  |  |  |  |  |  |  |  |  |  |
| PFREUD_06720 |  |  |  | Two-component system sensor kinase |  | ST |  | 0.35 | 4.06 | 3.71 | 3.24 |  | 0.000 |
| PFREUD_00810 |  |  |  | Sensor protein, ATPase-like:Histidine kinase |  | ST |  | -0.29 | -1.30 | -1.30 | -0.54 |  | 0.016 |
| PFREUD_21970 |  |  |  | Two component sensor kinase |  | ST |  | 0.12 | 1.20 | 1.05 | 1.34 |  | 0.019 |
| **Transport and biding of inorganic ions, proteins, peptides and lipoproteins** | | |  |  |  |  |  |  |  |  |  |  |  |
| PFREUD_05930 |  | *bopA* |  | Solute binding protein of the ABC transport system |  | T |  | -0.12 | -2.08 | -2.14 | -2.34 |  | 0.003 |
| PFREUD_04570 |  | *cbiQ2* |  | Cobalt transport protein CbiQ |  | T |  | 0.20 | -0.73 | -1.05 | -0.96 |  | 0.031 |
| PFREUD_04580 |  | *cbiN* |  | Cobalt transport protein CbiN |  | T |  | 0.00 | -1.98 | -2.26 | -2.10 |  | 0.001 |
| PFREUD_04590 |  | *cbiM* |  | Cobalt transport protein CbiM |  | T |  | 0.02 | -2.99 | -2.94 | -3.36 |  | 0.000 |
| PFREUD_07430 |  |  |  | Cation transport protein |  | T |  | -0.40 | -1.04 | -0.90 | -0.77 |  | 0.049 |
| PFREUD_09160 |  |  |  | IM protein of metallic cation ABC transporter |  | T |  | -0.21 | 1.88 | 1.49 | 0.44 |  | 0.006 |
| PFREUD_09170 |  | *mntB* |  | Manganese/zinc transport system ABC transporter ATP-binding protein |  | T |  | -0.24 | 2.99 | 2.35 | 0.33 |  | 0.002 |
| PFREUD_19490 |  |  |  | Cation transporting p-type ATPase 3.A.3.5.4 |  | T |  | 0.86 | 2.70 | 2.62 | 1.63 |  | 0.006 |
| PFREUD_19650 |  | *feoB* |  | Ferrous iron uptake protein B 9.A.8.1.x |  | T |  | -0.84 | 1.00 | 1.53 | 1.08 |  | 0.004 |
| PFREUD_19660 |  | *feoA* |  | Ferrous iron uptake protein A 9.a.8.1.x |  | T |  | -0.49 | 2.38 | 2.05 | 1.02 |  | 0.002 |
| PFREUD_19980 |  | *pstB* |  | Phosphate import ATP-binding protein pstB |  | T |  | -0.64 | -1.93 | -1.82 | -1.70 |  | 0.004 |
| PFREUD_20400 |  | *corA* |  | CorA, Mg2+ and Co2+ transporters |  | T |  | -0.90 | -1.41 | -1.08 | -0.50 |  | 0.024 |
| PFREUD_20420 |  |  |  | Ammonium transporter |  | T |  | 1.71 | -0.88 | -0.55 | -1.82 |  | 0.002 |
| PFREUD_22800 |  | *kup* |  | Low affinity potassium uptake system protein 2.A.72.1.1 |  | T |  | 0.18 | -2.50 | -2.31 | -1.99 |  | 0.000 |
| PFREUD_23180 |  |  |  | Transmembrane ATP-binding protein ABC transporter |  | T |  | -0.35 | 1.89 | 1.68 | -0.06 |  | 0.023 |
| PFREUD_23220 |  | *sseB* |  | Thiosulfate sulfurtransferase |  | T |  | 0.24 | 2.66 | 2.09 | 1.13 |  | 0.001 |
| PFREUD_21430 |  | *citT* |  | Divalent anion:Na+ symporter 2.A.47.3.x |  | T |  | 1.08 | 3.70 | 2.98 | 1.93 |  | 0.000 |
| PFREUD_00330 |  |  |  | Transporter |  | T |  | -0.77 | -1.46 | -1.32 | -0.85 |  | 0.026 |
| PFREUD_01270 |  |  |  | ABC 2 protein of drug ABC transporter |  | T |  | 0.22 | 1.84 | 1.45 | 1.65 |  | 0.004 |
| PFREUD_01280 |  |  |  | IM protein of drug ABC transporter |  | T |  | 0.28 | 1.55 | 1.14 | 1.24 |  | 0.008 |
| PFREUD_01380 |  | *ptsG/ptsM* |  | PTS system mannose-specific EIIBCA component |  | T |  | 0.43 | 1.11 | 0.87 | 1.41 |  | 0.048 |
| PFREUD_02090 |  |  |  | ABC transporter, trans-membrane region |  | T |  | -0.85 | -1.02 | -0.97 | -0.80 |  | 0.005 |
| PFREUD_02100 |  |  |  | IM-ABC protein of drug, protein or lipid ABC transporter |  | T |  | -0.97 | -1.34 | -1.32 | -1.10 |  | 0.005 |
| PFREUD_02590 |  |  |  | BP protein of siderophore ABC transporter |  | T |  | -1.28 | 0.55 | 0.50 | -0.05 |  | 0.002 |
| PFREUD_03570 |  | *PPA1043* |  | Anaerobic C4-dicarboxylate transporter |  | T |  | 2.02 | 2.75 | 2.75 | 2.37 |  | 0.000 |
| PFREUD_05060 |  |  |  | Membrane efflux protein MFS |  | T |  | -0.23 | -1.78 | -1.68 | -1.44 |  | 0.001 |
| PFREUD_05960 |  |  |  | IM protein of oligopeptide ABC transporter |  | T |  | -0.07 | -2.00 | -1.98 | -1.75 |  | 0.000 |
| PFREUD_06900 |  |  |  | Hypothetical protein |  | T |  | -0.55 | -1.47 | -1.36 | -1.34 |  | 0.003 |
| PFREUD_07970 |  |  |  | ABC2 protein of ABC transporter |  | T |  | -0.49 | -2.32 | -2.22 | -2.07 |  | 0.001 |
| PFREUD_08830 |  |  |  | Transporter, MFS superfamily protein |  | T |  | -0.13 | 0.58 | 1.16 | 1.04 |  | 0.003 |
| PFREUD_09180 |  |  |  | BP protein of metallic cation (Zn^2+^/Mn^2+^) ABC transporter |  | T |  | -0.08 | 2.57 | 1.50 | 0.06 |  | 0.001 |
| PFREUD_09590 |  | *ftsE* |  | ABC transporter, ATP-binding protein |  | T |  | -0.74 | -3.13 | -2.79 | -1.80 |  | 0.000 |
| PFREUD_09690 |  |  |  | Pre-protein translocase SecA subunit |  | T |  | 0.00 | -1.56 | -1.46 | -1.68 |  | 0.024 |
| PFREUD_11230 |  |  |  | Protein of ABC transporter |  | T |  | -0.10 | -1.95 | -1.85 | -2.55 |  | 0.001 |
| PFREUD_11240 |  |  |  | Protein of ABC transporteur |  | T |  | -0.15 | -2.03 | -1.71 | -1.82 |  | 0.000 |
| PFREUD_11960 |  |  |  | BP protein of monosaccharide ABC transporter |  | T |  | 0.06 | -3.65 | -2.98 | -3.48 |  | 0.000 |
| PFREUD_14830 |  |  |  | Transporter, sodium/bile acid symporter family |  | T |  | 0.21 | -1.62 | -1.46 | -1.24 |  | 0.000 |
| PFREUD_14910 |  |  |  | ABC2 protein of ABC transporter |  | T |  | -1.17 | -2.16 | -2.09 | -1.73 |  | 0.003 |
| PFREUD_14950 |  | *sufC* |  | ABC-type transport system involved in Fe-S cluster assembly, ATPase component, SufC |  | T |  | -1.07 | 0.16 | 0.04 | -1.78 |  | 0.016 |
| PFREUD_14970 |  | *sufD* |  | FeS assembly protein SufD |  | T |  | -1.19 | 0.14 | -0.17 | -2.42 |  | 0.018 |
| PFREUD_16480 |  |  |  | Major facilitator superfamily drug:H+ antiporter 2.A.1.3.x |  | T |  | 4.83 | 2.30 | 2.39 | 2.12 |  | 0.000 |
| PFREUD_19480 |  |  |  | Phosphocarrier protein, HPr family 8.A.8.1.1 |  | T |  | 0.68 | 2.57 | 2.20 | 1.04 |  | 0.039 |
| PFREUD_22940 |  |  |  | Polar amino acid ABC transporter, binding protein component |  | T |  | -0.25 | -0.57 | -0.77 | -1.07 |  | 0.029 |
| PFREUD_23420 |  |  |  | Extracellular solute-binding protein precursor |  | T |  | 0.13 | 1.00 | 0.72 | 0.74 |  | 0.025 |
| PFREUD_24030 |  | *mscs* |  | MscS transporter, small conductance mechanosensitive ion channel |  | T |  | 0.30 | 2.92 | 1.84 | 2.36 |  | 0.001 |
| **Transcription initiation, regulation, elogation and terminaison, and RNA modification** | | |  |  |  |  |  |  |  |  |  |  |  |
| PFREUD_15580 |  | *mraW* |  | S-adenosyl-L-methionine-dependent methyltransferase |  | TS |  | -0.53 | 1.51 | 2.83 | 3.33 |  | 0.000 |
| PFREUD_06510 |  |  |  | Transcription initiation factor |  | TS |  | 0.62 | -2.39 | -2.11 | -2.40 |  | 0.001 |
| PFREUD_10200 |  | *rpoE* |  | RNA polymerase sigma -70 factor, sigma-E factor |  | TS |  | 1.44 | -0.02 | -0.36 | -1.44 |  | 0.033 |
| PFREUD_00270 |  | *lacI* |  | Transcriptional regulator, LacI family protein |  | TS |  | 0.60 | 2.61 | 2.05 | 1.59 |  | 0.000 |
| PFREUD_00310 |  |  |  | Two-component system response regulator |  | TS |  | 0.25 | 1.10 | 1.07 | 1.38 |  | 0.029 |
| PFREUD_01400 |  |  |  | Response regulator, two-component system |  | TS |  | 0.14 | 1.70 | 1.35 | 1.10 |  | 0.002 |
| PFREUD_02060 |  | *mar* |  | Transcriptional regulators MarR |  | TS |  | -0.06 | 0.82 | 1.41 | 1.59 |  | 0.037 |
| PFREUD_02110 |  |  |  | Transcription regulator |  | TS |  | -0.80 | -1.22 | -1.26 | -1.15 |  | 0.008 |
| PFREUD_02390 |  |  |  | Transcriptional regulator |  | TS |  | 0.17 | 2.11 | 1.93 | 1.34 |  | 0.007 |
| PFREUD_04600 |  | *gntR* |  | Transcription factor |  | TS |  | -0.16 | 1.31 | 0.86 | -0.15 |  | 0.000 |
| PFREUD_08140 |  | *tetR5* |  | Transcriptional regulator TetR |  | TS |  | -0.50 | 2.27 | 2.42 | 1.03 |  | 0.007 |
| PFREUD_08960 |  | *pocR* |  | Transcriptional regulator |  | TS |  | 0.79 | 1.03 | 0.92 | 1.10 |  | 0.000 |
| PFREUD_09660 |  |  |  | Two-component system response regulator |  | TS |  | -0.61 | -1.47 | -0.81 | -0.59 |  | 0.040 |
| PFREUD_10380 |  |  |  | Sua5/YciO/YrdC/YwlC |  | TS |  | -0.20 | -0.72 | -1.12 | -2.00 |  | 0.020 |
| PFREUD_10980 |  |  |  | AsnC-family transcriptional regulatory protein |  | TS |  | -0.29 | -1.75 | -1.53 | -1.56 |  | 0.000 |
| PFREUD_11220 |  | *tetR6* |  | Regulatory protein TetR |  | TS |  | 0.01 | -1.33 | -1.33 | -1.60 |  | 0.006 |
| PFREUD_13290 |  | *iclR* |  | IclR transcriptional regulator |  | TS |  | 0.00 | 1.72 | 1.79 | 0.33 |  | 0.048 |
| PFREUD_14460 |  | *rnc* |  | Ribonuclease III |  | TS |  | -0.16 | -1.35 | -1.08 | -1.07 |  | 0.032 |
| PFREUD_14990 |  | *arsR4* |  | Transcriptional regulator, ArsR family |  | TS |  | -0.69 | 1.38 | 0.48 | -0.89 |  | 0.002 |
| PFREUD_15840 |  |  |  | Response regulator receiver protein |  | TS |  | 0.18 | -3.47 | -3.37 | -3.17 |  | 0.000 |
| PFREUD_15890 |  | *pspA* |  | Phage shock protein A |  | TS |  | 0.43 | 1.86 | 1.73 | 0.96 |  | 0.046 |
| PFREUD_16520 |  | *lysR1* |  | Transcriptional regulator, LysR family protein |  | TS |  | 0.47 | 2.31 | 2.16 | 3.03 |  | 0.003 |
| PFREUD_17650 |  | *tetR8* |  | Regulatory protein TetR |  | TS |  | -0.05 | 1.22 | 0.96 | 0.64 |  | 0.002 |
| PFREUD_17810 |  | *hspR1* |  | Heat shock protein transcriptional repressor HspR1 |  | TS |  | -0.74 | -2.81 | -2.54 | -2.38 |  | 0.000 |
| PFREUD_18150 |  | *dtxR* |  | Iron-dependent repressor |  | TS |  | 1.04 | 2.51 | 1.92 | 1.22 |  | 0.017 |
| PFREUD_18770 |  | *carD* |  | Transcriptional regulator CarD |  | TS |  | 0.25 | 5.25 | 4.44 | 3.23 |  | 0.003 |
| PFREUD_18780 |  | *regX* |  | Response regulator receiver |  | TS |  | 0.20 | 3.80 | 3.00 | 2.00 |  | 0.001 |
| PFREUD_19050 |  |  |  | GntR-family protein transcriptional regulator |  | TS |  | -0.20 | -0.80 | -1.03 | -1.15 |  | 0.038 |
| PFREUD_21980 |  |  |  | Two component transcriptional regulator, LuxR family |  | TS |  | 0.15 | 1.31 | 1.04 | 0.49 |  | 0.017 |
| PFREUD_22770 |  |  |  | Transcriptional regulator |  | TS |  | 0.36 | 2.50 | 1.69 | 0.86 |  | 0.022 |
| PFREUD_22900 |  |  |  | Transcriptional regulator PadR family |  | TS |  | -0.58 | 3.61 | 3.34 | 2.40 |  | 0.001 |
| PFREUD_23210 |  | *lacI4* |  | Transcriptional regulator, LacI family |  | TS |  | 0.34 | 1.63 | 1.49 | 1.61 |  | 0.001 |
| PFREUD_23960 |  | *tetR1* |  | Transcriptional regulator, TetR family |  | TS |  | 0.10 | 1.83 | 1.69 | 0.73 |  | 0.047 |
| PFREUD_05600 |  | *rpoB* |  | DNA-directed RNA polymerase beta chain |  | TS |  | -0.49 | -1.29 | -0.82 | -0.25 |  | 0.007 |
| PFREUD_06070 |  | *rpoA* |  | DNA-directed RNA polymerase alpha chain |  | TS |  | -1.20 | -3.96 | -3.40 | -3.69 |  | 0.022 |
| PFREUD_05520 |  | *nusG* |  | Transcription antitermination protein NusG |  | TS |  | -1.24 | -3.41 | -2.89 | -1.98 |  | 0.000 |
| PFREUD_11500 |  | *nusB* |  | Transcription antitermination factor |  | TS |  | -0.40 | -1.82 | -1.61 | -1.49 |  | 0.009 |
| PFREUD_01390 |  |  |  | Exonuclease of the beta-lactamase fold involved in RNA processing |  | TS |  | 0.65 | 3.65 | 3.58 | 2.92 |  | 0.000 |
| PFREUD_09270 |  | *dus* |  | TIM-barrel enzyme, dihydrouridine synthase |  | TS |  | -0.32 | -2.72 | -2.33 | -2.30 |  | 0.000 |
| PFREUD_14340 |  | *trmD* |  | tRNA (guanine-N1-)-methyltransferase |  | TS |  | -0.51 | -1.90 | -1.60 | -1.54 |  | 0.001 |
| PFREUD_19170 |  | *spoU3* |  | tRNA/rRNA methyltransferase |  | TS |  | -0.54 | -1.54 | -1.21 | -1.15 |  | 0.004 |
| PFREUD_24040 |  | *pcnB* |  | tRNA nucleotidyltransferase PcnB |  | TS |  | -0.52 | -1.87 | -1.49 | -1.51 |  | 0.001 |
| PFREUD_24210 |  | *rnpA* |  | Ribonuclease P protein component |  | TS |  | 0.07 | -2.72 | -2.76 | -2.73 |  | 0.001 |
| **Other and hypothetical proteins** | | |  |  |  |  |  |  |  |  |  |  |  |
| PFREUD_21640 |  | *mmcV/cysD* |  | Sulfate adenylyltransferase subunit 2 |  |  |  | -1.19 | -1.64 | -1.34 | -1.04 |  | 0.019 |
| PFREUD_15860 |  |  |  | HesB protein |  |  |  | -0.42 | -2.35 | -1.96 | -1.78 |  | 0.006 |
| PFREUD_15970 |  | *glnA1* |  | Glutamine synthetase |  |  |  | 1.35 | -1.86 | -1.45 | -2.20 |  | 0.035 |
| PFREUD_04210 |  | *int* |  | Phage integrase |  |  |  | 0.02 | -1.37 | -1.42 | -1.05 |  | 0.000 |
| PFREUD_21010 |  |  |  | Abortive infection bacteriophage resistance protein |  |  |  | 0.04 | 0.46 | 0.80 | 1.00 |  | 0.008 |
| PFREUD_03030 |  |  |  | Hypothetical protein |  |  |  | 0.69 | 1.82 | 1.57 | 1.25 |  | 0.001 |
| PFREUD_00170 |  |  |  | Hypothetical protein |  |  |  | 0.05 | 1.66 | 1.45 | 2.00 |  | 0.024 |
| PFREUD_00190 |  |  |  | Hypothetical protein |  |  |  | 0.08 | 1.32 | 0.99 | 0.56 |  | 0.016 |
| PFREUD_00200 |  |  |  | Hypothetical protein |  |  |  | -0.18 | 1.37 | 1.32 | 1.00 |  | 0.004 |
| PFREUD_00460 |  |  |  | Hypothetical trans-membrane protein |  |  |  | -0.41 | -1.67 | -1.61 | -1.13 |  | 0.004 |
| PFREUD_00910 |  |  |  | Hypothetical membrane protein |  |  |  | -0.47 | -1.22 | -1.21 | -0.95 |  | 0.001 |
| PFREUD_01100 |  |  |  | Hypothetical protein |  |  |  | 0.48 | 3.66 | 3.06 | 2.29 |  | 0.001 |
| PFREUD_01490 |  |  |  | Membrane protein without function |  |  |  | 0.11 | 1.49 | 0.71 | 0.14 |  | 0.010 |
| PFREUD_01920 |  |  |  | Hypothetical protein |  |  |  | -0.19 | -0.99 | -1.01 | -0.87 |  | 0.001 |
| PFREUD_02140 |  |  |  | Hypothetical protein |  |  |  | -0.19 | 2.75 | 2.25 | 1.05 |  | 0.001 |
| PFREUD_02220 |  |  |  | Hypothetical protein |  |  |  | 0.70 | 2.78 | 2.45 | 1.69 |  | 0.003 |
| PFREUD_02270 |  |  |  | Hypothetical protein |  |  |  | -0.28 | -1.40 | -0.98 | -0.83 |  | 0.007 |
| PFREUD_02290 |  |  |  | Hypothetical secreted protein |  |  |  | 0.50 | 2.33 | 2.38 | 1.73 |  | 0.001 |
| PFREUD_03020 |  |  |  | Hypothetical protein |  |  |  | 0.00 | -1.27 | -1.31 | -0.76 |  | 0.008 |
| PFREUD_03120 |  |  |  | Hypothetical protein |  |  |  | -0.23 | -2.34 | -2.31 | -2.61 |  | 0.000 |
| PFREUD_03130 |  |  |  | Hypothetical membrane protein |  |  |  | -0.65 | -1.19 | -1.35 | -0.82 |  | 0.028 |
| PFREUD_04700 |  |  |  | Hypothetical protein |  |  |  | -0.83 | -1.35 | -1.26 | -1.24 |  | 0.014 |
| PFREUD_05000 |  | *pf1420* |  | Hypothetical protein |  |  |  | 0.29 | -1.01 | -0.74 | -1.46 |  | 0.024 |
| PFREUD_05010 |  | *pf2416* |  | Hypothetical protein |  |  |  | -0.29 | -1.39 | -1.40 | -1.65 |  | 0.003 |
| PFREUD_05360 |  |  |  | Hypothetical membrane protein |  |  |  | 0.12 | 1.35 | 0.79 | -0.08 |  | 0.040 |
| PFREUD_06880 |  |  |  | Hypothetical protein |  |  |  | -0.18 | -2.03 | -1.75 | -1.95 |  | 0.000 |
| PFREUD_07380 |  |  |  | Hypothetical protein |  |  |  | 0.21 | 1.32 | 1.08 | 0.79 |  | 0.013 |
| PFREUD_07410 |  |  |  | Hypothetical protein |  |  |  | 0.16 | -1.74 | -2.13 | -2.15 |  | 0.007 |
| PFREUD_07800 |  |  |  | Hypothetical protein |  |  |  | -0.04 | -0.70 | -0.79 | -1.19 |  | 0.050 |
| PFREUD_07950 |  |  |  | Hypothetical protein |  |  |  | -0.11 | -1.07 | -1.07 | -1.05 |  | 0.004 |
| PFREUD_08070 |  | *pf2652* |  | Hypothetical protein |  |  |  | -0.20 | -1.60 | -1.03 | -0.83 |  | 0.029 |
| PFREUD_08340 |  |  |  | Hypothetical membrane protein |  |  |  | -0.25 | -3.08 | -3.03 | -3.24 |  | 0.000 |
| PFREUD_09480 |  |  |  | Hypothetical protein |  |  |  | 0.46 | -0.75 | -1.57 | -2.18 |  | 0.018 |
| PFREUD_09640 |  |  |  | Hypothetical trans-membrane protein |  |  |  | 0.04 | 2.06 | 1.82 | 1.61 |  | 0.000 |
| PFREUD_09960 |  |  |  | Hypothetical secreted and membrane protein |  |  |  | -0.01 | -1.13 | -1.09 | -0.74 |  | 0.001 |
| PFREUD_10510 |  |  |  | Hypothetical secreted protein |  |  |  | -1.01 | -2.33 | -2.58 | -3.10 |  | 0.008 |
| PFREUD_10570 |  |  |  | Hypothetical protein |  |  |  | 0.19 | -2.42 | -2.12 | -2.38 |  | 0.002 |
| PFREUD_11990 |  |  |  | Hypothetical protein |  |  |  | 1.21 | -0.52 | -0.68 | -0.90 |  | 0.005 |
| PFREUD_12730 |  |  |  | Hypothetical protein |  |  |  | 0.38 | -1.70 | -1.47 | -1.87 |  | 0.007 |
| PFREUD_15180 |  |  |  | Hypothetical protein |  |  |  | 0.14 | 1.06 | 1.06 | 0.74 |  | 0.013 |
| PFREUD_15610 |  |  |  | Hypothetical membrane protein |  |  |  | 0.81 | 3.87 | 2.96 | 1.65 |  | 0.002 |
| PFREUD_15780 |  |  |  | Hypothetical protein |  |  |  | 0.46 | 3.93 | 3.97 | 3.70 |  | 0.000 |
| PFREUD_15810 |  |  |  | Hypothetical protein |  |  |  | 0.20 | 1.05 | 1.23 | 1.89 |  | 0.004 |
| PFREUD_16200 |  |  |  | Hypothetical protein |  |  |  | 0.35 | 2.85 | 1.90 | 0.42 |  | 0.024 |
| PFREUD_16210 |  |  |  | Hypothetical membrane protein |  |  |  | 0.21 | 2.19 | 1.16 | -0.03 |  | 0.012 |
| PFREUD_16260 |  |  |  | Hypothetical secreted protein |  |  |  | -0.94 | -2.72 | -2.58 | -2.46 |  | 0.000 |
| PFREUD_17000 |  |  |  | Hypothetical membrane protein |  |  |  | 0.35 | 4.16 | 3.68 | 2.97 |  | 0.000 |
| PFREUD_17070 |  |  |  | Hypothetical protein |  |  |  | 0.22 | 2.95 | 2.48 | 1.63 |  | 0.003 |
| PFREUD_18540 |  |  |  | Hypothetical protein |  |  |  | -0.06 | 1.10 | 0.80 | 0.63 |  | 0.003 |
| PFREUD_19180 |  | *dedD* |  | Conserved membrane protein |  |  |  | 0.02 | -1.10 | -1.20 | -1.27 |  | 0.029 |
| PFREUD_19190 |  | *lemA* |  | Trans-membrane protein |  |  |  | 0.15 | 1.33 | 0.94 | 0.88 |  | 0.040 |
| PFREUD_20140 |  |  |  | Hypothetical protein |  |  |  | 0.26 | 1.06 | 0.30 | -0.65 |  | 0.006 |
| PFREUD_20770 |  |  |  | Hypothetical protein |  |  |  | -0.71 | -2.15 | -2.33 | -2.34 |  | 0.000 |
| PFREUD_20800 |  |  |  | Hypothetical protein |  |  |  | 0.39 | -2.50 | -2.35 | -2.28 |  | 0.001 |
| PFREUD_20890 |  |  |  | Hypothetical protein |  |  |  | 0.02 | 0.95 | 1.05 | 0.89 |  | 0.001 |
| PFREUD_21070 |  |  |  | Hypothetical protein |  |  |  | 0.38 | 1.12 | 1.05 | 1.61 |  | 0.013 |
| PFREUD_21700 |  |  |  | Hypothetical fusion protein |  |  |  | -0.04 | 1.34 | 1.26 | 0.94 |  | 0.002 |
| PFREUD_22320 |  |  |  | Hypothetical protein |  |  |  | -0.18 | 1.25 | 1.34 | 0.42 |  | 0.030 |
| PFREUD_22660 |  |  |  | Hypothetical protein |  |  |  | 0.01 | 3.11 | 2.65 | 0.76 |  | 0.010 |
| PFREUD_22710 |  |  |  | Hypothetical protein |  |  |  | 0.12 | 2.42 | 2.53 | 2.76 |  | 0.000 |
| PFREUD_22760 |  |  |  | Hypothetical protein |  |  |  | 0.35 | 1.63 | 2.14 | 2.22 |  | 0.005 |
| PFREUD_23680 |  |  |  | Hypothetical protein |  |  |  | 0.24 | -0.81 | -1.28 | -1.11 |  | 0.001 |
| PFREUD_24200 |  |  |  | Hypothetical protein |  |  |  | 0.23 | -3.33 | -3.12 | -2.70 |  | 0.000 |
| PFREUD_00720 |  |  |  | Hypothetical protein |  |  |  | 0.53 | 2.38 | 1.91 | 2.36 |  | 0.000 |
| PFREUD_00930 |  |  |  | Hypothetical protein |  |  |  | 0.35 | 2.22 | 1.98 | 1.74 |  | 0.000 |
| PFREUD_01110 |  |  |  | Hypothetical protein |  |  |  | 0.94 | 2.28 | 1.86 | 1.69 |  | 0.003 |
| PFREUD_01900 |  |  |  | Hypothetical secreted protein |  |  |  | 0.17 | -0.66 | -0.78 | -1.55 |  | 0.004 |
| PFREUD_02020 |  |  |  | Hypothetical protein |  |  |  | 0.13 | 2.31 | 1.93 | 1.43 |  | 0.001 |
| PFREUD_02900 |  |  |  | Hypothetical protein |  |  |  | 0.34 | 1.78 | 1.97 | 1.79 |  | 0.000 |
| PFREUD_02910 |  |  |  | Hypothetical protein |  |  |  | 1.38 | 2.71 | 2.53 | 2.13 |  | 0.002 |
| PFREUD_03150 |  |  |  | Hypothetical protein |  |  |  | 0.69 | 1.36 | 0.86 | 0.96 |  | 0.019 |
| PFREUD_03500 |  |  |  | Hypothetical protein |  |  |  | 0.36 | 1.61 | 1.72 | 1.58 |  | 0.000 |
| PFREUD_03520 |  |  |  | Hypothetical protein |  |  |  | 0.11 | 0.88 | 1.61 | 1.55 |  | 0.013 |
| PFREUD_03530 |  |  |  | Hypothetical protein |  |  |  | 0.20 | 1.36 | 1.89 | 2.12 |  | 0.001 |
| PFREUD_03540 |  |  |  | Hypothetical protein |  |  |  | 0.24 | 1.37 | 2.08 | 2.26 |  | 0.000 |
| PFREUD_03550 |  |  |  | Hypothetical protein |  |  |  | 0.12 | 0.39 | 1.07 | 1.23 |  | 0.018 |
| PFREUD_03580 |  |  |  | Hypothetical protein |  |  |  | 0.36 | 1.01 | 1.03 | 1.62 |  | 0.039 |
| PFREUD_03950 |  |  |  | Hypothetical protein |  |  |  | -0.18 | -1.37 | -1.38 | -1.19 |  | 0.000 |
| PFREUD_03960 |  |  |  | Hypothetical protein |  |  |  | -0.18 | -2.49 | -2.46 | -1.92 |  | 0.000 |
| PFREUD_03970 |  |  |  | Hypothetical protein |  |  |  | -0.02 | -2.89 | -2.71 | -1.90 |  | 0.000 |
| PFREUD_04100 |  |  |  | Hypothetical protein |  |  |  | -0.13 | 2.89 | 2.74 | 2.07 |  | 0.000 |
| PFREUD_04120 |  |  |  | protein of unknown function |  |  |  | -0.02 | -1.09 | -1.29 | -1.15 |  | 0.015 |
| PFREUD_04130 |  |  |  | Hypothetical protein |  |  |  | -0.37 | -1.78 | -1.85 | -1.72 |  | 0.001 |
| PFREUD_04280 |  |  |  | Hypothetical protein |  |  |  | -0.45 | -1.91 | -1.85 | -2.09 |  | 0.001 |
| PFREUD_04710 |  |  |  | Hypothetical protein |  |  |  | -1.05 | -2.33 | -2.07 | -2.07 |  | 0.002 |
| PFREUD_04980 |  |  |  | Hypothetical protein |  |  |  | 0.16 | -0.91 | -1.07 | -1.31 |  | 0.025 |
| PFREUD_06170 |  |  |  | Hypothetical protein |  |  |  | -0.12 | -1.71 | -1.66 | -1.81 |  | 0.000 |
| PFREUD_06180 |  |  |  | Hypothetical protein |  |  |  | -0.03 | -3.64 | -2.83 | -2.66 |  | 0.000 |
| PFREUD_06740 |  |  |  | Hypothetical protein |  |  |  | 0.43 | 3.23 | 2.15 | 0.56 |  | 0.039 |
| PFREUD_07670 |  |  |  | Hypothetical protein |  |  |  | 1.45 | 1.93 | 2.25 | 2.29 |  | 0.000 |
| PFREUD_07740 |  |  |  | Hypothetical protein |  |  |  | 0.21 | -1.07 | -1.09 | -1.20 |  | 0.017 |
| PFREUD_08560 |  |  |  | Hypothetical protein |  |  |  | 0.51 | 1.09 | 0.83 | 1.00 |  | 0.018 |
| PFREUD_08890 |  |  |  | Hypothetical protein |  |  |  | 0.83 | 2.11 | 1.06 | -0.04 |  | 0.033 |
| PFREUD_09610 |  |  |  | Hypothetical protein |  |  |  | -0.63 | -1.58 | -1.51 | -1.31 |  | 0.000 |
| PFREUD_10100 |  |  |  | Hypothetical protein |  |  |  | -0.95 | -1.71 | -1.75 | -1.83 |  | 0.000 |
| PFREUD_10410 |  |  |  | Hypothetical protein |  |  |  | 0.85 | 0.54 | 0.80 | 1.16 |  | 0.013 |
| PFREUD_10680 |  |  |  | Hypothetical protein |  |  |  | 1.11 | -2.69 | -2.36 | -2.50 |  | 0.000 |
| PFREUD_10690 |  |  |  | Hypothetical secreted protein |  |  |  | 1.03 | -2.62 | -2.11 | -2.02 |  | 0.000 |
| PFREUD_11490 |  |  |  | Hypothetical protein |  |  |  | -0.58 | -2.56 | -2.21 | -2.09 |  | 0.003 |
| PFREUD_13370 |  |  |  | Hypothetical protein |  |  |  | 1.09 | -2.25 | -2.36 | -2.13 |  | 0.000 |
| PFREUD_13380 |  |  |  | Hypothetical secreted protein |  |  |  | 1.30 | -2.55 | -2.64 | -2.39 |  | 0.000 |
| PFREUD_13500 |  |  |  | Hypothetical protein |  |  |  | 0.00 | -1.15 | -1.09 | -1.17 |  | 0.015 |
| PFREUD_13760 |  |  |  | Hypothetical protein |  |  |  | -0.24 | 0.03 | 0.88 | 2.00 |  | 0.019 |
| PFREUD_14030 |  |  |  | Hypothetical protein |  |  |  | -0.24 | -1.20 | -0.78 | -0.79 |  | 0.013 |
| PFREUD_15020 |  |  |  | Hypothetical membrane protein |  |  |  | -0.05 | -1.02 | -0.68 | -0.67 |  | 0.002 |
| PFREUD_16770 |  |  |  | Hypothetical protein |  |  |  | -0.38 | -1.46 | -1.41 | -1.38 |  | 0.000 |
| PFREUD_16850 |  |  |  | Hypothetical protein |  |  |  | 0.43 | 0.93 | 0.92 | 1.31 |  | 0.013 |
| PFREUD_17160 |  |  |  | Hypothetical protein |  |  |  | 1.67 | 2.62 | 1.33 | 0.82 |  | 0.015 |
| PFREUD_17870 |  |  |  | Hypothetical protein |  |  |  | 0.12 | -1.46 | -1.13 | -0.58 |  | 0.015 |
| PFREUD_17880 |  |  |  | Hypothetical protein |  |  |  | -0.22 | -1.01 | -0.83 | -0.50 |  | 0.007 |
| PFREUD_19720 |  |  |  | Hypothetical protein |  |  |  | 0.24 | 2.12 | 1.86 | 0.91 |  | 0.038 |
| PFREUD_19750 |  |  |  | Hypothetical protein |  |  |  | 0.38 | 1.08 | 0.57 | 0.25 |  | 0.026 |
| PFREUD_20450 |  |  |  | Hypothetical protein |  |  |  | 0.22 | 1.18 | 1.05 | 1.20 |  | 0.002 |
| PFREUD_20760 |  |  |  | Hypothetical protein |  |  |  | 0.02 | -1.39 | -1.36 | -1.10 |  | 0.001 |
| PFREUD_20880 |  |  |  | Hypothetical protein |  |  |  | -0.48 | -1.13 | -1.04 | -0.97 |  | 0.011 |
| PFREUD_20960 |  |  |  | Hypothetical protein |  |  |  | -0.72 | -1.42 | -1.31 | -1.48 |  | 0.001 |
| PFREUD_21370 |  |  |  | Hypothetical protein |  |  |  | 0.05 | 0.03 | 0.17 | 1.09 |  | 0.004 |
| PFREUD_22150 |  |  |  | Hypothetical protein |  |  |  | 0.21 | 2.16 | 2.09 | 1.93 |  | 0.019 |
| PFREUD_22170 |  |  |  | Hypothetical protein |  |  |  | -0.13 | -1.69 | -1.89 | -1.59 |  | 0.000 |
| PFREUD_22180 |  |  |  | Hypothetical protein |  |  |  | -0.83 | -2.03 | -1.84 | -1.61 |  | 0.000 |
| PFREUD_22310 |  |  |  | Hypothetical protein |  |  |  | -0.04 | 1.38 | 1.19 | 0.98 |  | 0.001 |
| PFREUD_22580 |  |  |  | Hypothetical protein |  |  |  | 0.46 | 1.17 | 1.19 | 1.78 |  | 0.007 |
| PFREUD_22740 |  |  |  | Hypothetical protein |  |  |  | 0.75 | 1.98 | 1.93 | 2.71 |  | 0.026 |
| PFREUD_23260 |  |  |  | Hypothetical protein |  |  |  | -0.27 | -1.45 | -1.25 | -1.54 |  | 0.003 |
| PFREUD_23650 |  |  |  | Hypothetical protein |  |  |  | -0.32 | -1.93 | -1.82 | -1.82 |  | 0.001 |
| PFREUD_23750 |  |  |  | Hypothetical protein |  |  |  | 0.23 | 1.20 | 0.68 | -0.42 |  | 0.047 |
| PFREUD_23880 |  |  |  | Hypothetical protein |  |  |  | 0.94 | 2.46 | 2.10 | 1.02 |  | 0.019 |
| PFREUD_24130 |  |  |  | Hypothetical protein |  |  |  | 0.05 | -0.81 | -1.30 | -1.35 |  | 0.016 |
| PFREUD_24220 |  |  |  | Hypothetical protein |  |  |  | 0.07 | -1.95 | -1.93 | -2.12 |  | 0.002 |
| PFREUD_00070 |  |  |  | ABC transporter |  |  |  | -0.02 | -1.29 | -1.26 | -1.08 |  | 0.018 |
| PFREUD_01670 |  |  |  | Acetyltransferase |  |  |  | 0.36 | 2.18 | 2.62 | 2.41 |  | 0.003 |
| PFREUD_03100 |  | *kduD* |  | 2-deoxy-D-gluconate 3-dehydrogenase |  |  |  | 0.13 | 0.72 | 0.97 | 1.36 |  | 0.001 |
| PFREUD_04950 |  | *KradDRAFT_4229* |  | Zinc-containing alcohol dehydrogenase superfamily |  |  |  | 0.27 | 1.53 | 1.20 | 0.96 |  | 0.008 |
| PFREUD_11260 |  | *engA* |  | GTP binding protein |  |  |  | 0.39 | 1.67 | 1.36 | 0.79 |  | 0.024 |
| PFREUD_11440 |  | *mihF* |  | Integration host factor MihF |  |  |  | 2.32 | -3.68 | -3.34 | -3.38 |  | 0.000 |
| PFREUD_14980 |  | *sufB* |  | FeS assembly protein SufB |  |  |  | -1.21 | -0.06 | -0.47 | -3.12 |  | 0.006 |
| PFREUD_24180 |  | *jag* |  | Single-stranded nucleic acid binding R3H |  |  |  | 0.00 | -1.66 | -1.23 | -1.23 |  | 0.003 |
