# Supplementary material for: A Temporal -omic Study of Propionibacterium freudenreichii CIRM-BIA1T Adaptation Strategies in Conditions Mimicking Cheese Ripening in the Cold
Source: PLoS One. 2012 Jan 13;7(1):e29083. doi: 10.1371/journal.pone.0029083 (PMC3258244; doi:10.1371/journal.pone.0029083)
Supplement: Table S3 — Description of the metabolic function of genes grouped in each category. (DOC) [file pone.0029083.s005.doc]

**Table S3**: Description of the metabolic function of genes grouped in each category.

| **Code** | **Gene category** | **Metabolic function** |
| --- | --- | --- |
| A | Adaptation to atypical conditions | Adaptation to atypical conditions |
| AA | Transport and metabolism of amino acids | Transport/binding of amino acids |
|  |  | Metabolism of amino acids and related molecules |
| C | Metabolism of coenzymes and prosthetic groups | Metabolism of coenzymes and prosthetic groups |
| CD | Cell division | Cell division |
| CE | Cell envelop | Cell wall |
|  |  | Cell envelope and cellular processes |
| CH | Transport and metabolism of carbohydrates | Transport/binding of carbohydrates |
|  |  | Specific carbohydrate metabolic pathway |
|  |  | Main glycolytic pathways |
|  |  | TCA cycle |
|  |  | Metabolism of carbohydrates and related molecules |
| DNA | DNA metabolism | DNA replication |
|  |  | DNA restriction and modification (and repair) |
|  |  | DNA recombination and repair |
| E | Energy metabolism | Membrane bioenergetics (electron transport chain and ATP synthase) |
| L | Lipid metabolism | Metabolism of lipids |
| Mi | Miscellaneous | Detoxification |
|  |  | Miscellaneous |
| Nt | Transport and metabolism of nucleotides | Transport/binding of nucleosides, nucleotides, purines and pyrimidines |
|  |  | Metabolism of nucleotides and nucleic acids |
|  |  | Metabolism of coenzymes and prosthetic groups |
| P | Protein synthesis | Ribosomal proteins |
|  |  | Aminoacyl-tRNA synthetases |
|  |  | Translation initiation |
|  |  | Translation elongation |
|  |  | Nonribosomal protein synthesis |
|  |  | Protein synthesis |
| PD | Protein degradation | Protein degradation |
| Ph | Metabolism of phosphate | Metabolism of phosphate |
| PM | Protein modification and folding | Protein modification |
|  |  | Protein folding |
| PS | Protein secretion | Protein secretion |
| ST | Signal transduction | Sensors (signal transduction) |
| T | Transport of peptides and inorganic ions | Transport/binding of proteins peptides |
|  |  | Transport/binding of inorganic ions |
|  |  | Transport/binding of carbohydrates |
|  |  | Transport/binding proteins and lipoproteins |
| TS | Transcription | Transcription initiation |
|  |  | Transcription regulation |
|  |  | Transcription elongation |
|  |  | Transcription termination |
|  |  | RNA modification |
