# Supplementary material for: A Temporal -omic Study of Propionibacterium freudenreichii CIRM-BIA1T Adaptation Strategies in Conditions Mimicking Cheese Ripening in the Cold
Source: PLoS One. 2012 Jan 13;7(1):e29083. doi: 10.1371/journal.pone.0029083 (PMC3258244; doi:10.1371/journal.pone.0029083)
Supplement: Table S4 — Proteins from P. freudenreichii CIRM-BIA1T strain identified by tandem mass spectrometry. (DOC) [file pone.0029083.s006.doc]

**Table S4**: Proteins from *P. freudenreichii* CIRM-BIA1^T^ strain identified by tandem mass spectrometry.

| **Protein** | |  | **Gene** | | |  | **MS** | | | | | |
| --- | --- | --- | --- | --- | --- | --- | --- | --- | --- | --- | --- | --- |
| **Spot No.^a^** | **Description** |  | **Name** | **Locus tag** | **Category** |  | **Mascot score** | **Sequence coverage (%)** | **No. of peptide identified** | **emPAI^b^** | **Exp.(MS^c^) pI** | **Exp.(MS^d^) MW (kDa)** |
| 1 | 30S ribosomal protein S1 |  | *rps*A | PFREUD_15330 | P |  | 1756.68 | 55.58 | 23 | 3.19 | 4.39(4.61) | 66(53.58) |
|  | Chaperone protein dnaK 2 |  | *dna*K2 | PFREUD_04630 | PM |  | 1450.22 | 36.86 | 19 | 1.86 | 4.39(4.66) | 66(67.19) |
| 2 | NADH-quinone oxidoreductase chain D |  | *nuo*D | PFREUD_05190 | E |  | 1087.63 | 49.02 | 14 | 1.60 | 4.64(4.84) | 60.26(50.28) |
|  | Phosphate acetyltransferase |  | *pta* | PFREUD_00700 | CH |  | 564.46 | 22.35 | 8 | 0.62 | 4.64(4.75) | 60.26(52.84) |
|  | Aldehyde dehydrogenase |  | *gab* | PFREUD_15340 | CH |  | 206.48 | 5.74 | 2 | 0.13 | 4.64(4.86) | 60.26(52.24) |
|  | Pyruvate phosphate dikinase |  | *ppdk* | PFREUD_03230 | CH |  | 160.13 | 4.63 | 2 | 0.07 | 4.64(4.81) | 60.26(95.82) |
| 3 | Elongation factor Tu |  | *tuf* | PFREUD_05650 | P |  | 941.26 | 40.40 | 11 | 1.78 | 4.89(5.05) | 56.49(43.65) |
|  | Diaminopimelate decarboxylase |  | *lys*A | PFREUD_10300 | AA |  | 137.24 | 5.16 | 2 | 0.11 | 4.89(5.93) | 56.49(58.96) |
| 4 | Glyceraldehyde-3-phosphate dehydrogenase |  | *gap* | PFREUD_15130 | CH |  | 1308.73 | 61.38 | 17 | 3.86 | 5.14(5.26) | 48.44(36.09) |
| 5 | cysteine synthase 2 |  | *cys*2 | PFREUD_16420 | AA |  | 1343.72 | 70.81 | 16 | 4.47 | 5.07(5.14) | 42.63(33.62) |
|  | Fructose-bisphosphate aldolase class II |  | *fba*1 | PFREUD_19150 | CH |  | 659.69 | 37.94 | 9 | 1.37 | 5.07(5.21) | 42.63(36.85) |
|  | Fructose-bisphosphate aldolase class I |  | *fba*2 | PFREUD_23890 | CH |  | 422.44 | 29.83 | 6 | 0.80 | 5.07(5.02) | 42.63(32.31) |
|  | Carboxylic ester hydrolase |  | *pf3004* | PFREUD_09440 | L |  | 178.73 | 10.06 | 2 | 0.21 | 5.07(5.24) | 42.63(32.95) |
|  | Glyceraldehyde-3-phosphate dehydrogenase |  | *gap* | PFREUD_15130 | CH |  | 161.54 | 9.28 | 2 | 0.19 | 5.07(5.26) | 42.63(36.09) |
| 6 | Hypothetical protein |  |  | PFREUD_18630 |  |  | 880.48 | 59.17 | 12 | 4.08 | 4.43(4.7) | 34.59(23.17) |
|  | Electron transfer flavoprotein |  | *fix*A | PFREUD_02480 | E |  | 667.45 | 55.38 | 7 | 1.36 | 4.43(4.72) | 34.59(25.71) |
|  | Two-component system response regulator |  |  | PFREUD_15370 | TS |  | 391.42 | 40.58 | 6 | 1.30 | 4.43(4.63) | 34.59(22.59) |
|  | groEL1 |  | *gro*EL1 | PFREUD_06470 | PM |  | 377.21 | 15.47 | 5 | 0.33 | 4.43(4.72) | 34.59(56.13) |
|  | NADH-quinone oxidoreductase chain E |  |  | PFREUD_05200 | E |  | 116.33 | 11.93 | 2 | 0.27 | 4.43(4.87) | 34.59(25.92) |
| 7 | Heat shock protein 20 1 |  | *hsp*20 1 | PFREUD_22780 | PM |  | 747.62 | 68.55 | 8 | 3.20 | 4.49(4.73) | 20.27(17.23) |
|  | Heat shock protein 20 2 |  | *hsp*20 2 | PFREUD_09500 | PM |  | 253.57 | 26.67 | 3 | 0.74 | 4.49(4.92) | 20.27(16.83) |
| 8 | 50S ribosomal protein L7/L12 |  | *rpl*L | PFREUD_05580 | P |  | 680.77 | 77.86 | 9 | 10.64 | 4.35(4.46) | 19.18(13.66) |
|  | Heat shock protein 20 2 |  | *hsp*20 2 | PFREUD_09500 | PM |  | 263.02 | 33.33 | 3 | 0.74 | 4.35(4.92) | 19.18(16.83) |
|  | Heat shock protein 20 1 |  | *hsp*20 1 | PFREUD_22780 | PM |  | 208.75 | 22.01 | 2 | 0.43 | 4.35(4.73) | 19.18(17.23) |
| 9 | groS1 |  | *gro*S1 | PFREUD_06460 | PM |  | 395.11 | 56.12 | 5 | 3.13 | 4.86(4.95) | 15.49(10.59) |
|  | Transcarboxylase, 1.3S subunit |  | *bccp* | PFREUD_18840 | CH |  | 203.08 | 34.96 | 3 | 1.09 | 4.86(5.4) | 15.49(12.36) |
| 10 | Alanyl-tRNA synthetase |  | *ala*S | PFREUD_11560 | P |  | 866.89 | 18.66 | 11 | 0.44 | 5.21(5.23) | >95(97.04) |
|  | Alpha-glucan phosphorylase |  | *glg*P | PFREUD_10670 | CH |  | 724.27 | 17.17 | 9 | 0.36 | 5.21(5.18) | >95(94.62) |
| 11 | chaperone clpC |  | *clp*C | PFREUD_20250 | PM |  | 864.00 | 19.81 | 13 | 0.56 | 5.11(5.19) | >95(94.29) |
|  | Putative DNA polymerase I |  | *pol*A | PFREUD_15360 | DNA |  | 637.69 | 14.46 | 9 | 0.34 | 5.11(5.04) | >95(98.57) |
|  | Pyruvate synthase |  | *nif*J1 | PFREUD_01840 | E |  | 96.37 | 2.47 | 2 | 0.05 | 5.11(5.22) | >95(136.58) |
| 12 | Pyruvate phosphate dikinase |  | *ppdK* | PFREUD_03230 | CH |  | 3157.06 | 56.95 | 36 | 3.69 | 4.72(4.81) | >95(95.82) |
| 13 | Chaperone clpB 1 |  | *clpB* 1 | PFREUD_19250 | PM |  | 2514.31 | 42.54 | 31 | 2.46 | 5.01(5.07) | >95(93.46) |
|  | Pyruvate phosphate dikinase |  | *ppdk* | PFREUD_03230 | CH |  | 181.54 | 4.63 | 2 | 0.07 | 5.01(4.81) | >95(95.82) |
| 14 | Chaperone clpB 2 |  | *clpB* 2 | PFREUD_17920 | PM |  | 2248.11 | 37.56 | 30 | 2.31 | 5.38(5.33) | 97(94.32) |
| 15 | Transketolase |  | *tkt* | PFREUD_22360 | CH |  | 1055.86 | 28.20 | 14 | 1.00 | 4.88(4.96) | 80.97(74.06) |
|  | Aspartyl-tRNA synthetase |  | *asp*S | PFREUD_11590 | P |  | 109.14 | 6.99 | 2 | 0.10 | 4.88(4.95) | 80.97(67.34) |
| 16 | Methylmalonyl-CoA mutase large subunit |  | *mut*B | PFREUD_07650 | CH |  | 1330.95 | 35.71 | 17 | 1.14 | 5.27(5.21) | 78.83(80.19) |
| 17 | Phosphoribosyltransferase |  | *blu*B | PFREUD_06370 | C |  | 1273.69 | 31.31 | 15 | 1.39 | 4.89(5.06) | 71.88(66.37) |
| 18 | Methylmalonyl-CoA mutase small subunit |  | *mut*A | PFREUD_07660 | CH |  | 1257.19 | 33.86 | 17 | 1.29 | 4.97(5) | 65.29(69.63) |
| 19 | Fatty-acyl-CoA synthase |  | *acs* | PFREUD_23780 | L |  | 454.86 | 17.30 | 7 | 0.44 | 5.04(5.08) | 64.07(61.01) |
| 20 | Methionyl-tRNA synthetase |  | *met*G | PFREUD_06960 | P |  | 472.38 | 17.89 | 7 | 0.51 | 4.71(4.68) | 63.88(62.61) |
|  | Alpha-1,4-glucosidase |  | *agl*A | PFREUD_23740 | CH |  | 542.28 | 17.76 | 6 | 0.36 | 4.71(4.88) | 63.88(63.36) |
| 21 | Inosine-5 -monophosphate dehydrogenase |  | *gua*B1 | PFREUD_06480 | Nt |  | 1438.85 | 44.86 | 18 | 2.92 | 5.76(5.49) | 63.88(53.88) |
| 22 | ATP synthase subunit alpha |  | *atp*A | PFREUD_10470 | E |  | 938.21 | 36.27 | 13 | 0.97 | 5.03(5.06) | 63.5(61.34) |
|  | UDP-N-acetylglucosamine pyrophosphorylase |  | *glm*U | PFREUD_17410 | Nt |  | 673.28 | 31.26 | 10 | 1.02 | 5.03(5.11) | 63.5(54.49) |
| 23 | UDP-N-acetylglucosamine pyrophosphorylase |  | *glm*U | PFREUD_17410 | Nt |  | 512.62 | 18.83 | 7 | 0.51 | 5.11(5.11) | 63.41(54.49) |
| 24 | GMP synthase |  | *gua*A | PFREUD_06680 | Nt |  | 763.27 | 27.85 | 10 | 0.78 | 4.75(4.89) | 63.22(55.6) |
|  | ATP synthase subunit alpha |  | *atp*A | PFREUD_10470 | E |  | 416.91 | 18.66 | 7 | 0.44 | 4.75(5.06) | 63.22(61.34) |
|  | Glutamine synthetase |  | *gln*A1 | PFREUD_15970 |  |  | 215.94 | 11.06 | 4 | 0.27 | 4.75(4.84) | 63.22(54.12) |
|  | Gluconate kinase |  | *gnt*K | PFREUD_01040 | CH |  | 154.96 | 5.87 | 2 | 0.12 | 4.75(4.9) | 63.22(55.03) |
| 25 | Transcarboxylase 12S subunit |  | *mmd*A | PFREUD_18870 | CH |  | 713.57 | 18.00 | 10 | 0.63 | 5.66(5.09) | 62.42(65.89) |
|  | FAD-dependent pyridine nucleotide-disulphide oxidoreductase |  |  | PFREUD_01830 | E |  | 636.36 | 21.73 | 9 | 0.62 | 5.66(5.45) | 62.42(59.84) |
|  | Transcarboxylase 5S subunit |  |  | PFREUD_18870 | CH |  | 99.20 | 6.34 | 2 | 0.12 | 5.66(5.5) | 62.42(55.61) |
| 26 | Thiamine pyrophosphate enzyme |  |  | PFREUD_04430 | C |  | 1598.25 | 42.05 | 20 | 1.92 | 5.02(5.06) | 61.67(62.89) |
| 27 | Glucose-6-phosphate isomerase |  | *pgi* | PFREUD_04290 | CH |  | 757.56 | 27.32 | 9 | 0.77 | 5.19(5.16) | 61.34(61.84) |
|  | Formate-tetrahydrofolate ligase |  | *fhs* | PFREUD_04720 | CH |  | 597.37 | 21.15 | 9 | 0.72 | 5.19(5.15) | 61.34(59.1) |
|  | Pyruvate kinase 1 |  | *pyk*1 | PFREUD_15390 | CH |  | 442.72 | 16.23 | 6 | 0.51 | 5.19(5.17) | 61.34(54.01) |
| 28 | Catalase |  | *kat*A | PFREUD_23800 | A |  | 963.47 | 41.49 | 12 | 1.31 | 5.05(5.05) | 61.15(53.57) |
| 29 | Phosphoglucomutase |  | *pgm*1 | PFREUD_10610 | CH |  | 1179.18 | 35.73 | 16 | 1.54 | 4.86(5.03) | 60.82(58.58) |
|  | Methylmalonic acid semialdehyde dehydrogenase |  | *iol*A | PFREUD_19100 | CH |  | 314.81 | 12.77 | 4 | 0.27 | 4.86(5) | 60.82(52.79) |
| 30 | Methylmalonic acid semialdehyde dehydrogenase |  | *iol*A | PFREUD_19100 | CH |  | 1116.77 | 32.73 | 14 | 1.48 | 5.02(5) | 60.77(52.79) |
| 31 | Transcarboxylase 5S subunit |  |  | PFREUD_18870 | CH |  | 1059.31 | 34.46 | 15 | 2.17 | 5.51(5.38) | 60.59(55.58) |
|  | Phosphoribosylaminoimidazolecarboxamide formyltransferase |  | *pur*H | PFREUD_06830 | Nt |  | 181.47 | 10.25 | 3 | 0.19 | 5.51(5.42) | 60.59(54.52) |
|  | FAD-dependent pyridine nucleotide-disulphide oxidoreductase |  |  | PFREUD_01830 | E |  | 107.47 | 4.60 | 2 | 0.11 | 5.51(5.45) | 60.59(59.84) |
| 32 | Coenzyme A transferase |  | *cat* | PFREUD_03110 | CH |  | 1027.44 | 37.87 | 14 | 1.51 | 5.82(5.53) | 59.88(55.65) |
| 33 | Aspartate ammonia-lyase |  | *asp*A2 | PFREUD_16330 | AA |  | 730.01 | 26.94 | 12 | 1.19 | 5.1(5.12) | 59.46(53.07) |
| 34 | Dihydrolipoyl dehydrogenase |  | *ldp* | PFREUD_10890 | CH |  | 757.07 | 36.48 | 11 | 1.31 | 5.06(5.08) | 58.18(49.59) |
| 35 | Fumarate hydratase, class-II |  | *fum*C | PFREUD_16300 | CH |  | 1152.92 | 42.83 | 17 | 2.72 | 5.47(5.34) | 57.62(51.15) |
| 36 | Alanine dehydrogenase |  | *ald* | PFREUD_00370 | AA |  | 1057.20 | 46.63 | 14 | 2.65 | 4.73(4.81) | 56.91(39.38) |
| 37 | Enolase 1 |  | *eno*1 | PFREUD_17320 | CH |  | 1444.14 | 52.45 | 16 | 2.75 | 4.34(4.47) | 55.69(45.92) |
| 38 | Anaerobic glycerol-3-phosphate dehydrogenase subunit B |  | *glp*B | PFREUD_12980 | CH |  | 898.79 | 39.45 | 13 | 1.47 | 5.35(5.27) | 55.36(45.88) |
|  | Coenzyme F420-dependent N5,N10-methylene tetrahydromethanopterin reductase |  | *mer* | PFREUD_05920 | E |  | 154.35 | 13.82 | 3 | 0.24 | 5.35(5.24) | 55.36(44.62) |
| 39 | Hypothetical protein |  |  | PFREUD_03120 |  |  | 813.01 | 40.40 | 10 | 1.60 | 4.8(4.87) | 53.1(43.38) |
| 40 | Dihydroorotate dehydrogenase |  |  | PFREUD_01850 | Nt |  | 761.13 | 37.14 | 10 | 1.77 | 4.63(4.76) | 50.98(40.72) |
|  | inositol-1-phosphate synthase |  |  | PFREUD_19880 | CH |  | 323.88 | 14.76 | 4 | 0.38 | 4.63(4.77) | 50.98(39.19) |
| 41 | Aspartate-semialdehyde dehydrogenase |  | *asd* | PFREUD_20100 | AA |  | 863.76 | 43.31 | 10 | 2.09 | 5.28(5.16) | 50.23(36.57) |
| 42 | Inosine-5 -monophosphate dehydrogenase |  | *gua*B2 | PFREUD_06490 | Nt |  | 1229.44 | 50.95 | 15 | 3.63 | 5.83(5.61) | 49.19(39.48) |
| 43 | Ketol-acid reductoisomerase |  | *ilv*C | PFREUD_13390 | AA |  | 784.24 | 39.07 | 10 | 2.04 | 4.99(5.01) | 46.79(37.16) |
|  | Branched-chain amino acid aminotransferase |  | *ilv*E | PFREUD_13350 | AA |  | 316.70 | 20.22 | 5 | 0.49 | 4.99(5.02) | 46.79(39.81) |
|  | Hypothetical protein |  |  | PFREUD_11600 |  |  | 121.28 | 6.44 | 2 | 0.18 | 4.99(5.13) | 46.79(39.4) |
| 44 | Hypothetical protein |  |  | PFREUD_15430 |  |  | 1301.53 | 47.29 | 15 | 4.23 | 4.55(4.78) | 46.55(36.51) |
| 45 | dolichyl-phosphate beta-D-mannosyltransferase |  | *dpm* | PFREUD_10950 | CE |  | 596.38 | 38.95 | 8 | 1.71 | 5.2(5.34) | 36.76(31.66) |
|  | NADH-quinone oxidoreductase chain C |  | *nuo*C | PFREUD_05180 | E |  | 439.29 | 26.27 | 7 | 1.16 | 5.2(5.36) | 36.76(28.68) |
| 46 | NADH-quinone oxidoreductase chain C |  | *nuo*C | PFREUD_05180 | E |  | 846.84 | 51.76 | 12 | 4.21 | 5.38(5.36) | 36.02(28.68) |
| 47 | Iron/Manganese superoxide dismutase |  | *sod*A | PFREUD_06110 | Mi |  | 313.22 | 19.31 | 4 | 0.73 | 5.33(5.28) | 26.98(22.83) |
| 48 | Methylmalonyl-CoA epimerase |  |  | PFREUD_10590 | CH |  | 512.04 | 64.19 | 7 | 3.40 | 5.55(5.4) | 24.16(16.71) |
| 49 | Hypothetical protein |  |  | PFREUD_11600 |  |  | 970.28 | 45.10 | 14 | 2.65 | 5.13(5.13) | 46.08(39.4) |
| 50 | DNA-directed RNA polymerase alpha chain |  | *rpo*A | PFREUD_06070 | TS |  | 1065.91 | 52.80 | 13 | 3.32 | 4.2(4.35) | 55.31(36.95) |
|  | Enolase 1 |  | *eno*1 | PFREUD_17320 | CH |  | 181.61 | 6.53 | 2 | 0.15 | 4.2(4.47) | 55.31(45.92) |

^a^Spot number (see Fig.3).

^b^Exponentially Modified Protein Abundance Index.

^c^Theoretical isoelectric point as determined from the predicted protein sequence.

^d^Theoretical molecular weight as determined from the predicted protein sequence.
